# Transcriptome Analysis of Gene Families Involved in Chemosensory Function in *Spodoptera littoralis* (Lepidoptera: Noctuidae)

**DOI:** 10.1186/s12864-019-5815-x

**Published:** 2019-05-28

**Authors:** William B. Walker, Amit Roy, Peter Anderson, Fredrik Schlyter, Bill S. Hansson, Mattias C. Larsson

**Affiliations:** 1 Department of Plant Protection Biology, Swedish University of Agricultural Sciences, Sundsvägen 14, 230 53 Alnarp, Sweden; 2 Faculty of Forestry and Wood Sciences, EXTEMIT-K, Czech University of Life Sciences, Kamýcká 1176, Prague 6, 165 21 Suchdol, Czech Republic; 3 Department of Evolutionary Neuroethology, Max Planck Institute for Chemical Ecology, 07745 Jena, Germany

**Keywords:** Transcriptomics, insect, Lepidoptera, *Spodoptera littoralis*, chemosensory, olfaction, gustation, antennae, brain, proboscis

## Abstract

**Background:**

Deciphering the molecular mechanisms mediating the chemical senses, taste, and smell has been of vital importance for understanding the nature of how insects interact with their chemical environment. Several gene families are implicated in the uptake, recognition, and termination of chemical signaling, including binding proteins, chemosensory receptors and degrading enzymes. The cotton leafworm, *Spodoptera littoralis*, is a phytophagous pest and current focal species for insect chemical ecology and neuroethology.

**Results:**

We produced male and female Illumina-based transcriptomes from chemosensory and non-chemosensory tissues of *S. littoralis,* including the antennae, proboscis, brain and body carcass. We have annotated 306 gene transcripts from eight gene families with known chemosensory function, including 114 novel candidate genes. Odorant receptors responsive to floral compounds are expressed in the proboscis and may play a role in guiding proboscis probing behavior. In both males and females, expression of gene transcripts with known chemosensory function, including odorant receptors and pheromone-binding proteins, has been observed in brain tissue, suggesting internal, non-sensory function for these genes.

**Conclusions:**

A well-curated set of annotated gene transcripts with putative chemosensory function is provided. This will serve as a resource for future chemosensory and transcriptomic studies in *S. littoralis* and closely related species. Collectively, our results expand current understanding of the expression patterns of genes with putative chemosensory function in insect sensory and non-sensory tissues. When coupled with functional data, such as the deorphanization of odorant receptors, the gene expression data can facilitate hypothesis generation, serving as a substrate for future studies.

**Electronic supplementary material:**

The online version of this article (10.1186/s12864-019-5815-x) contains supplementary material, which is available to authorized users.

## Background

The chemical senses, including the olfactory and gustatory modalities of smell and taste, play a critical role throughout the life history of most insects [1]. For phytophagous insects, including a great number of agricultural pest species, host plant determination and quality assessment, which is mediated by detection of host volatile blends by the insect’s olfactory system, are critical factors for successful oviposition and larval fitness [2, 3]. Furthermore, gustatory information about the host plant, detected by contact chemoreceptors, can mediate the decision to eat or not, and to oviposit or not [4].

At the molecular level the processes of chemosensory detection, which include uptake, reception, and inactivation of stimulus molecules [5], are mediated by a diversity of genes from several functionally interrelated gene families: odorant-binding proteins (OBPs) and chemosensory proteins (CSPs) during stimulus uptake; odorant receptors (ORs), ionotropic receptors (IRs) and gustatory receptors (GRs) during chemosensory stimulus reception; odorant-degrading enzymes (ODEs) including antennal-expressed carboxylesterase (CXEs) and cytochrome P450s (CYPs) during enzymatic degradation of the odorant molecules [5, 6]. These genes have been characterized primarily for their role in chemosensory processes. However, expression patterns of these genes in non-sensory tissues suggest the potential for diverse biological functions. For example, CSPs are expressed in all insect tissues, and some have clearly been shown to have non-chemosensory functions [7, 8]. The expression of a *Drosophila melanogaster* GR in the brain has been linked to internal sugar monitoring [9], and the characterization of ORs in the sperm of mosquitos [10] resulted in the proposal of a novel function for insect ORs in sperm chemotaxis.

The molecular mechanisms of insect olfaction are mostly understood through research in *D. melanogaster*. However, a recent report demonstrating polycistronic co-expression of four to six ORs in individual olfactory sensory neuron subtypes in a mosquito [11] highlights the need for more information from non-model species, including moths. The Egyptian Cotton Leafworm, *Spodoptera littoralis* (Lepidoptera, Noctuidae) is a phytophagous pest insect indigenous to Africa and the Middle East [12]. In recent decades, *S littoralis* has obtained status as an invasive species of continental Europe [13]. Accordingly, *S. littoralis* has lately been the subject of intensive focused research, specifically with regards to questions of chemical ecology and for providing a foundation for novel pest control strategies.

It has been demonstrated that the antennae of *S. littoralis* respond to a broad range of ecologically relevant pheromonal as well as host plant volatiles [14–16]. The molecular underpinnings of olfactory detection in *S. littoralis* have recently been investigated, culminating in the identification of a repertoire of ORs that are activated by many of the same odorants shown previously to activate olfactory sensory neurons in this species [17]. Modulatory interactions between pheromone and volatile host plant odors have been suggested to impact olfactory sensitivity in the antennae of *S. littoralis* [18]. Interestingly, such olfactory sensitivity and olfactory-guided behaviours are also modulated by age, mating status, and experience [19–23]. Furthermore, oviposition behavior has been shown to be mediated by olfactory cues [24].

In *S. littoralis*, initial reports have characterized chemosensory gene expression in male [25] and female [26] antennal transcriptomes derived from expressed sequence tag libraries. A third report examined chemosensory gene expression in male and female antennae and maxillary palps as well as in larval antennae, using next-generation RNA-sequencing (RNA-seq) methodologies [27]. These three studies were mostly qualitative, providing descriptive annotations on an array of candidate genes from OR, GR, IR, OBP, and CSP gene families. Additional reports have described antennal-expressed putative ODEs from the CXE [28] and CYP [29] gene families.

In this report, we have expanded on these studies by performing in-depth qualitative as well as preliminary quantitative analyses of chemosensory gene expression in male and female *S. littoralis* antennae. Furthermore, we provide a comprehensive first-look at chemosensory gene expression in the proboscis of *S. littoralis*. We have also aimed to enhance the availability of adult *S. littoralis* transcriptomics resources in general and have thus sequenced transcripts from the brain and whole body minus head (henceforth body carcass) tissues. In sum, our study includes the analyses of male and female RNA-Seq transcriptomes, both derived from antennae, proboscis, brain and body carcass tissues. We have expanded the existing scientific knowledge on the number of putative chemosensory genes from most of the gene families with chemosensory function, as well as the completeness of coding sequence information for many of the previously identified but incomplete chemosensory transcripts. The expression of putative chemosensory receptor genes in brain tissue of both male and female moths suggests a potential novel function for these genes as monitors of internal chemical signaling. Finally, we report olfactory receptor genes that may mediate proboscis probing behaviors in moths.

## Results

### Transcriptome Overview

*De novo* transcriptomes were derived from nine tissue samples each for both male and female *S. littoralis.* After quality control processing of the raw sequencing reads, input for the male and female transcriptomes consisted of 240.8 and 236.4 million read pairs, respectively (Additional file 1). Subsequent to cd-hit-est redundancy removal, there were 1.24 x 10^5^ and 1.26 x 10^5^ sequences in the male and female transcriptomes respectively. Within the male transcriptome, there were 8.70 x 10^4^ component-level clusters, with 7.11 x 10^4^ of these containing only one sequence; within the female transcriptome, there were 8.82 x 10^4^ component clusters, with 7.22 x 10^4^ of these containing only one sequence (Additional file 2). BUSCO analysis of both transcriptomes with the Arthropoda database of single-copy orthologues, resulted in hits for 99.72-100% of queried sequences, with 95.97-96.06% identified as complete, indicating satisfactory completeness of the two transcriptomes.

### Annotation of Genes from Chemosensory Gene Families

An update to the repertoire of genes from previously described gene families with putative chemosensory function is reported here, with focus on novel genes identified in the OR, GR, IR, OBP, CSP, CXE/CCE, and CYP families. Additionally, updates have been made to previously-described incomplete genes belonging to these families. All novel genes and updates to previously annotated genes have been deposited in the Genbank Transcriptome Shotgun Assembly database, and information on these genes is provided (Additional file 3). Gene transcript nomenclature for novel genes has been coordinated with efforts to annotate chemosensory genes from the *S. frugiperda* genome project [30]. A comparison of peak expression abundance estimates for each gene family by tissue type and sex are presented to highlight similar trends for the different gene families discussed (Additional file 4).

### Odorant Receptors

A total of 60 odorant receptors were identified (Additional file 5). In the male transcriptome, transcripts for all ORs were identified except SlitOR48, and complete open reading frames (ORFs) were predicted for 48 ORs, based upon the presence of stop codons, predicted start codons and blast-based alignment to other sequences. In the female library, transcripts for all ORs were identified, and complete ORFs were predicted for 35 ORs.

For *S. littoralis,* 47 putative OR transcripts, including the OR co-receptor (ORCO) have previously been reported [25–27]. OR1-OR36 were cloned for functional studies [17]. For these ORs, consensus sequences identified in the transcriptomes here display 98-100% identity to the cloned OR sequences at the nucleotide level. OR38-OR47 were previously presented as incomplete fragments [27]. Here, we provide updated sequence information for OR38-OR46, with complete ORFs for OR38-OR45. Previously, OR47 was characterized as encoding 144 amino acids [27]; this transcript was not found in our female transcriptome, and in our male transcriptome, the ORF was only found as an incomplete fragment bracketed by in-frame stop codons. Based on this, and findings from the *S. frugiperda* genome [30], the previously annotated OR47 sequence has been replaced by a novel gene that displays homology to the previous fragment. Including OR47, a total of 14 novel ORs have been identified, and have been named in sequence, through OR60.

**Fig. 1:**
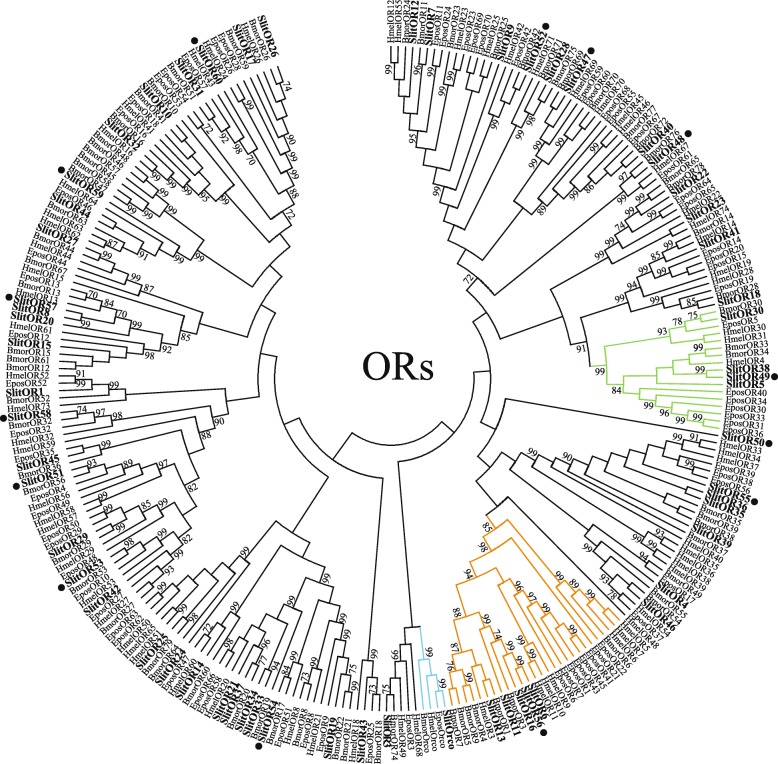
Unrooted Maximum likelihood phylogenetic tree of candidate ORs from *S. littoralis* and other Lepidoptera. The tree was built from an alignment of OR sequences from *S. littoralis* (Slit) *B. mori* (Bmor), *E. postvittana* (Epos) and *H. melpomene* (Hmel). Branches of the Orco clade are colored light blue; branches of the moth “pheromone receptor” clade are colored orange; branches of the secondary clade with sex-biased receptors are colored green; *S. littoralis* ORs are indicated with a larger bold font, and novel *S. littoralis* ORs are marked with a “•”. Node support was assessed with 600 bootstrap replicates and values greater than 70% are shown

Considering current and previous studies overall, complete ORFs are predicted for 58 of the 60 ORs. A phylogenetic tree indicating evolutionary relationships between *S. littoralis* ORs and a selection of those from other Lepidoptera with sequenced genomes is shown (Fig. 1).

**Fig. 2:**
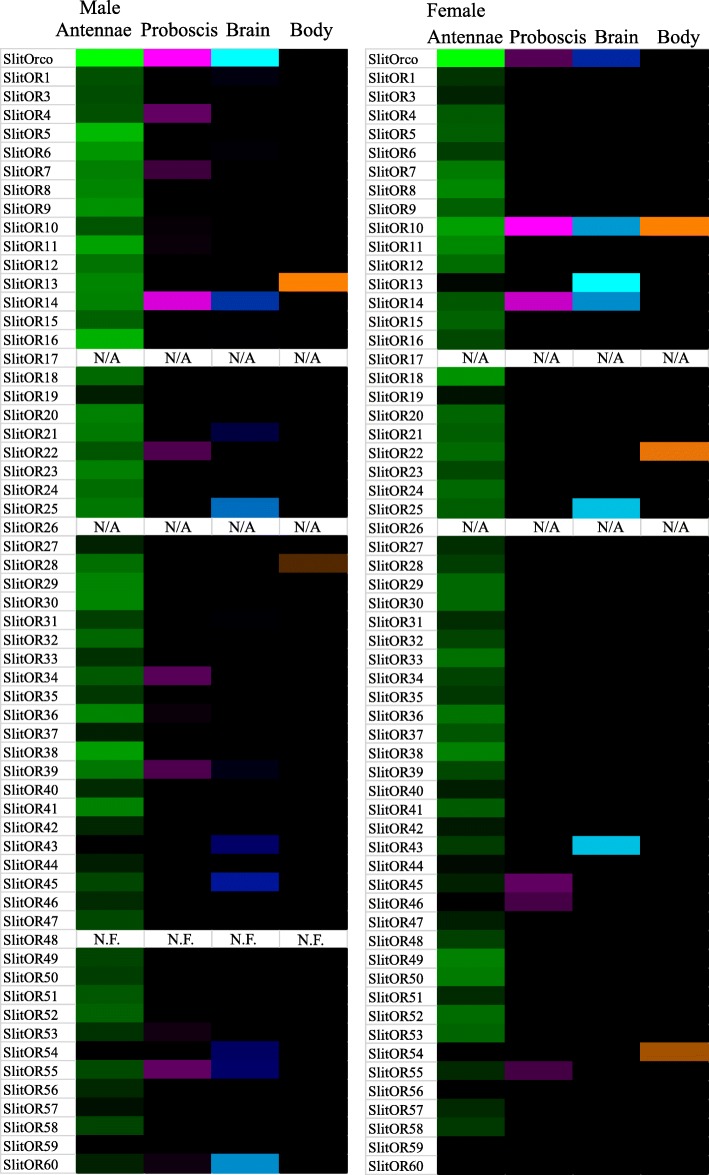
Heat-plot of relative expression values for SlitORs. Estimation of abundance values determined by read mapping. Black indicates low/no expression, dark colors indicate low/moderate expression, bright colors indicate moderate/high expression. Color plots represent binary log of FPKM plus one for each gene (See Additional file 6 for raw data). Color scales for each tissue type are independent of other tissue types. “N.F.” indicates that gene transcripts were not found in respective transcriptome. “N/A” indicates that unique gene model could not be resolved for gene transcripts in respective transcriptome due to co-assembly of highly similar gene models. Range of values for Male Antenna: 0.04 – 9.90; Male Brain: 0 – 2.88; Male Body: 0 – 1.57; Male Proboscis: 0 – 1.52; Female Antennae: 0 – 8.45; Female Brain: 0.00 – 2.25; Female Body: 0 – 1.55; Female Proboscis: 0 – 1.69

In virgin male antennae, estimated OR transcript abundance levels indicate that OR5 (71.5 Fragnents per Kiloboase per Million reads (FPKM)), OR16 (58.6 FPKM) and OR11 (34.4 FPKM) were the most abundantly expressed tuning ORs, of which the latter two cluster within the Lepidoptera pheromone receptor (PR) subfamily [31]. In virgin female antennae, OR10 (32.0 FPKM), OR18 (24.1 FPKM) and OR11 (19.0 FPKM) were the most abundantly expressed tuning ORs. Consistent with other reports, the OR co-receptor, Orco, was expressed at relatively higher levels compared to tuning ORs in both virgin male and female antennae (Fig. 2, Additional file 6).

**Fig. 3:**
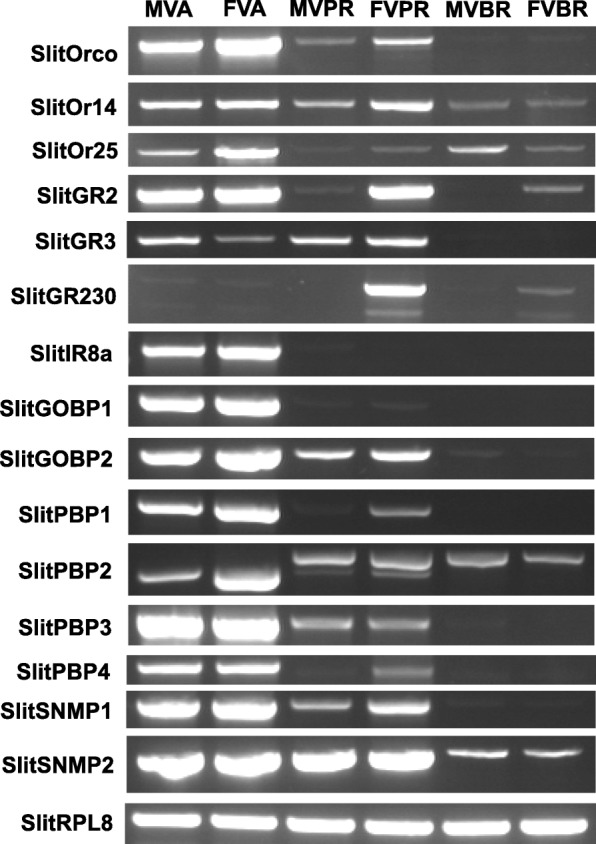
Expression profiles of selected chemosensory genes. Reverse transcription PCR (RT-PCR) assays were performed using gene specific primer pairs and cDNAs from different adult tissue: MVA – male virgin antennae, FVA – female virgin antennae, MVPR – male virgin proboscis, FVPR, female virgin proboscis, MVBR – male virgin brain, FVBR – female virgin brain. PCR products were analyzed on agarose gels pre-stained with Gel Red dye. Ribosomal protein, SlitRPL8, was used as a positive control for all samples

In other tissues, limited relatively low-level OR transcript expression was observed in both male and female samples. In the proboscis of both male and female moths, SlitOrco and SlitOR14 showed consistent expression patterns. In virgin female and male brain, SlitOrco, SlitOR14, and SlitOR25 displayed consistent expression patterns with FPKM values higher than one. (Additional file 6). RT-PCR assay of independent samples confirmed expression of both SlitOR14 and SlitOR25 in proboscis and brain tissues (Fig. 3).

#### Gustatory Receptors

A total of 17 predicted GRs have been annotated (Additional file 7); prior to this report, a total of six SlitGRs (SlitGR1-GR6) had been reported [26, 27]. In both the male and female transcriptomes, the previously identified SlitGR1 was identified in transcripts encoded as an incomplete ORF bracketed by in-frame stop codons and as such has been removed from consideration as a GR, and replaced by a novel transcript. To provide greater consistency with the nomenclature of GRs in *S. frugiperda* [30], previously annotated SlitGR4, SlitGR5, and SlitGR6 have been renamed as SlitGR10, SlitGR12, and SlitGR4, respectively, while SlitGR2 and SlitGR3 maintain the same nomenclature. Except for SlitGR10, all previously identified GRs were also found in our transcriptomes.

Twelve novel candidate GRs are described here, with complete ORFs predicted for five of these. The previously identified and incomplete, SlitGR12 has been RACE cloned, with a predicted complete ORF sequenced; at the nucleotide level, the cloned SlitGR12 shares 99% identity to transcripts identified here in the male and female transcriptomes.

**Fig. 4:**
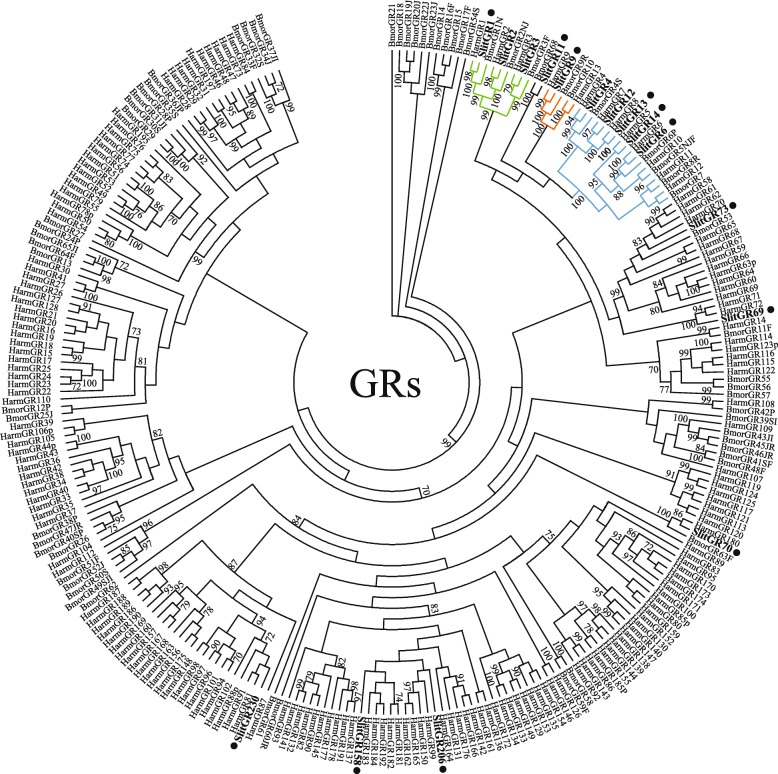
Maximum likelihood phylogenetic tree of candidate SlitGR sequences with other lepidopteran GR sequences. Unrooted. Includes sequences from *S. littoralis* (Slit), *Helicoverpa armigera* (Harm) and *Bombyx mori* (Bmor). Branches containing putative carbon dioxide receptors are colored green; branches containing putative sugar-compound receptors are colored blue; branches containing putative fructose receptors are colored orange; branches containing putative bitter-compound receptors are colored black; *S. littoralis* GRs are indicated with a larger bold font, and novel *S. littoralis* GRs are marked with a “•”. Node support was assessed with 600 bootstrap replicates and values greater than 70% are shown

In combining results from current and previous findings, complete ORFs are predicted for 8 of the 17 described SlitGRs. A phylogenetic tree indicating evolutionary relationships between *S. littoralis* GRs and a selection of those from other Lepidoptera with sequenced genomes is shown (Fig. 4).

In both virgin male and female antenna, transcripts encoding putative carbon dioxide (CO2) receptors, SlitGR2 and SlitGR3, represent the most highly expressed GRs (Additional files 6 and 8). RT-PCR assay also confirmed the expression of these two genes in the proboscis (Fig. 3).

In both male and female proboscis, transcript expression was observed consistently for a set of seven receptors. In both cases, putative sugar-compound receptors (SlitGR6, GR12, GR13, and GR14) were among the most highly expressed GRs in the proboscis. Notably, a single putative bitter-compound receptor, SlitGR230, was observed to be expressed across all tissue types. Expression of this gene was confirmed in antennae, proboscis, and brain via RT-PCR assay (Fig. 3).

#### Ionotropic Receptors

**Fig. 5:**
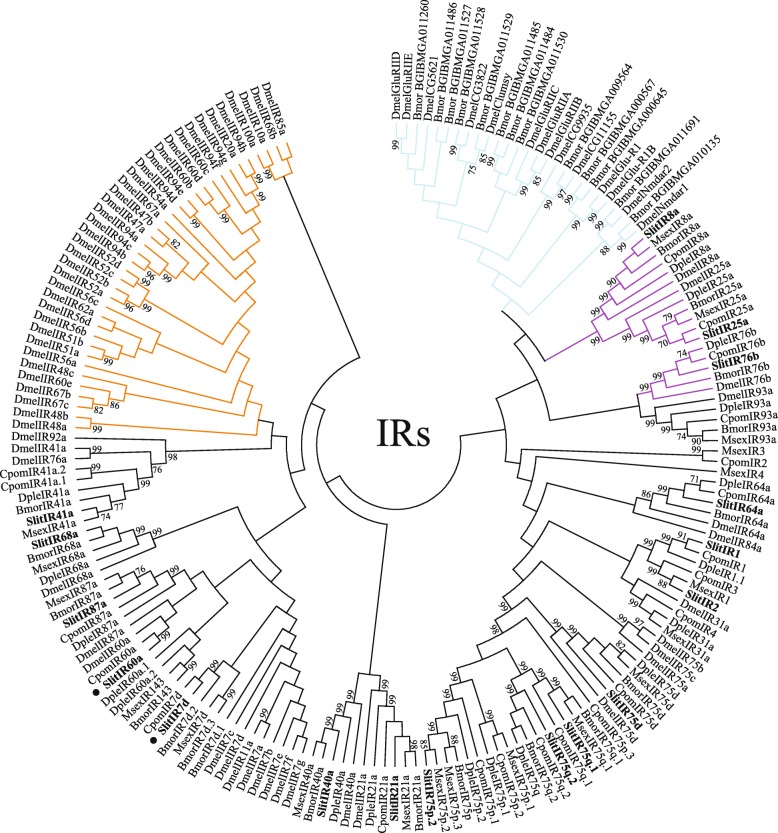
Maximum likelihood phylogenetic tree of candidate SlitIR sequences with other insect IR and iGluR sequences. Unrooted. Includes sequences from *S. littoralis* (Slit), *D. plexipus* (Dple), *Manduca sexta* (Msex), *Cydia pomonella* (Cpom), *Drosophila melanogaster* (Dmel) and *Bombyx mori* (Bmor). Branches containing putative ionotropic glutamate receptors (iGluRs) are colored light blue; branches containing putative IR co-receptors are colored purple; branches containing divergent IRs are colored orange; branches containing putative antennal IRs are colored black. *S. littoralis* IRs are indicated with a larger bold font, and novel *S. littoralis* ORs are marked with a “•”. Node support was assessed with 600 bootstrap replicates and values greater than 70% are shown

A total of 17 predicted SlitIRs are annotated here (Additional file 9), with gene transcripts for all 17 identified in both the male and female transcriptomes. Complete ORFs are predicted for eight of ten IRs previously reported as incomplete ORFs [27, 32]. In both the male and female transcriptomes, complete ORFs are predicted for 10 of the 17 IRs. Two new putative SlitIRs have been identified, SlitIR7d and SlitIR60a. Finally, previously reported gene transcripts for SlitIR2, SlitIR3 and SlitIR4 have been collapsed to a single gene transcript, SlitIR2; in both male and female transcripts, unigenes have been identified that comprise all three of these previously annotated SlitIRs. In sum, complete ORFs are predicted for all 17 of the putative SlitIRs described herein. A phylogenetic tree indicating evolutionary relationships between *S. littoralis* IRs and a selection of those from *D. melanogaster* and other Lepidoptera is shown (Fig. 5).

**Fig. 6:**
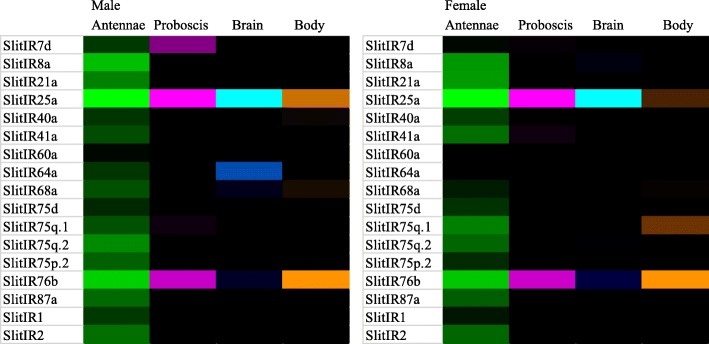
Heat-plot of relative expression values for SlitIRs. Estimation of abundance values determined by read mapping. Black indicates low/no expression, dark colors indicate low/moderate expression, bright colors indicate moderate/high expression. Color plots represent binary log of FPKM plus one for each gene (See Additional file 6 for raw data). Color scales for each tissue type are independent of other tissue types. Range of values for Male Antenna: 1.18 – 7.39; Male Brain: 0 – 2.28; Male Body: 0 – 2.88; Male Proboscis: 0 – 4.53; Female Antennae: 0.10 – 7.11; Female Brain: 0.00 – 2.15; Female Body: 0 – 1.39; Female Proboscis: 0 – 4.57

The putative IR co-receptors, SlitIR8a, SlitIR25a, and SlitIR76b, are the most abundantly expressed IR transcripts in the antennae of both virgin male and female *S. littoralis;* expression of Slit IR8a was only observed in the antennae (Figs. 3 and 6) Among IRs with a predicted role in odorant detection, SlitIR75q.2, SlitIR21a and SlitIR87a are the most abundantly expressed in both male and female antennae (Fig. 6, Additional file 6).

In the male and female proboscis, only SlitIR25a and SlitIR76b transcripts display FPKM abundance estimates consistently greater than one. In the brain of both virgin males and females, only the co-receptor Slit IR25a displays FPKM expression values greater than one.

#### Odorant-Binding Proteins

A total of 49 predicted OBPs have been identified across the male and female transcriptomes, including 16 novel gene transcripts (Additional file 10). All previously described OBPs [26, 27] were identified except for SlitOBP6, and complete ORF predictions have been made for nine of the ten remaining OBPs that were previously reported as incomplete [26, 27]. Previously annotated SlitOBP8 and SlitOBP19 have been removed from consideration as OBPs, due to greater degree of resemblance to juvenile hormone binding proteins; a novel sequence has been assigned as SlitOBP8. Due to similarity across sequences and the identification of only one unigene in both transcriptomes, the previously annotated SlitOBP7 and SlitOBP21 have been collapsed to a single gene transcript (SlitOBP21). In sum, complete ORFs are now predicted for 44 of the 49 described OBPs. A phylogenetic tree indicating evolutionary relationships between *S. littoralis* OBPs and a selection of those from other Lepidoptera with sequenced genomes is shown (Additional file 11).

**Fig. 7:**
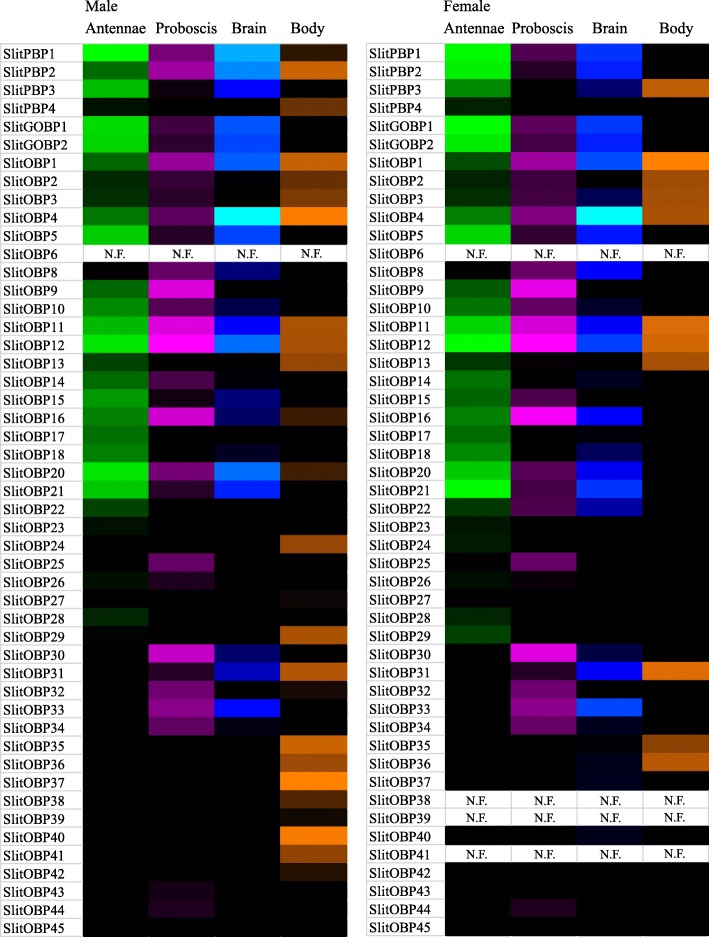
Heat-plot of relative expression values for SlitOBPs. Estimation of abundance values determined by read mapping. Black indicates low/no expression, dark colors indicate low/moderate expression, bright colors indicate moderate/high expression. Color plots represent binary log of FPKM plus one for each gene (See Additional file 6 for raw data). Color scales for each tissue type are independent of other tissue types. “N.F.” indicates that gene transcripts were not found in respective transcriptome. Range of values for Male Antenna: 0 – 16.14; Male Brain: 0 – 10.74; Male Body: 0 – 9.05; Male Proboscis: 0 – 11.71; Female Antennae: 0 – 14.28; Female Brain: 0 – 12.58; Female Body: 0 – 6.17; Female Proboscis: 0 – 11.04

OBPs displayed broad and diverse expression patterns in *S. littoralis* (Fig. 7, Additional file 6)*.* In both male and female antennae, the top five most abundantly expressed OBPs were the same, and consisted of SlitPBP1, SlitGOBP1, SlitGOBP2, SlitOBP12, and SlitOBP20, in varying rank order depending on the sex. In both male and female proboscis, the top five most abundantly expressed OBPs consisted of SlitOBP12, which was the most abundant in males and females, as well as SlitOBP9, SlitOBP11, SlitOBP16, and SlitOBP30, in varying rank order depending upon the sex. In both male and female brain, SlitOBP4 was the most abundantly expressed OBP. Further assay of GOBP and PBP gene expression, via RT-PCR, confirmed expression of these genes in the proboscis and also SlitPBP2 expression in the brain (Fig. 3).

#### Chemosensory Proteins

A total of 21 CSPs were identified including one novel sequence (Additional file 12). The previously reported SlitCSP3 was not identified in either transcriptome; due to its high similarity to SlitCSP4 and lack of identification here, it has been excluded from further annotation as a CSP. Otherwise, all gene transcripts were found in both male and female transcriptomes, with the exception that SlitCSP21 was not identified in females. Complete ORFs are predicted for the five CSPs previously reported as incomplete (SlitCSP10, 15, 16, 20, 21). In sum, complete ORFs are predicted for all CSPs across both transcriptomes. A phylogenetic tree indicating evolutionary relationships between *S. littoralis* CSPs and a selection of those from other Lepidoptera with sequenced genomes is shown (Additional file 13).

**Fig. 8:**
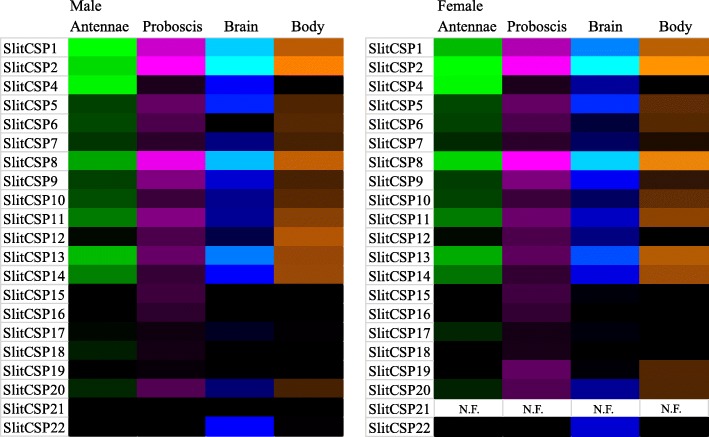
Heat-plot of relative expression values for SlitCSPs. Estimation of abundance values determined by read mapping. Black indicates low/no expression, dark colors indicate low/moderate expression, bright colors indicate moderate/high expression. Color plots represent binary log of FPKM plus one for each gene (See Additional file 6 for raw data). Color scales for each tissue type are independent of other tissue types. “N.F.” indicates that gene transcripts were not found in respective transcriptome. Range of values for Male Antenna: 0 – 12.62; Male Brain: 0 – 9.93; Male Body: 0 – 12.45; Male Proboscis: 0 – 15.05; Female Antennae: 0 – 12.61; Female Brain: 0 – 11.14; Female Body: 0 – 9.78; Female Proboscis: 0.45 – 15.72

Expression abundance estimates for SlitCSPs covered a broad range in all tissues examined (Fig. 8, Additional file 6). In all tissues examined, SlitCSP1, SlitCSP2, and SlitCSP8 were among the top five most abundant CSP transcripts and were collectively ranked as the top three, in varying order, in both male and female proboscis, brain and body carcass tissues.

#### Carboxyl/Choline Esterase Proteins

A total of 56 CCE genes are reported, including 30 previously described CXE genes [28, 33, 34], and 26 novel CCE transcripts (Additional file 14). Signal Peptide motifs were identified for 15 of the novel CCEs, and for 34 of the 56 gene transcripts characterized here, and previously [28, 33]. To provide greater consistency with annotations of the *S. frugiperda* genome consortium [30], as well as other noctuids [35], novel gene transcripts have been annotated as CCEs as opposed to CXEs. One exception is CXE8b, which has been named following CXE8 as it is a transcript variant of the same gene, sharing a common N terminal exon. Complete ORFs are predicted in our transcriptomes for three of six CXEs previously reported as incomplete (CXE18, 20, 30).

In sum, complete ORFs are predicted for 45 of the 56 gene transcripts. A phylogenetic tree indicating evolutionary relationships between *S. littoralis* CXE/CCEs and a selection of those from other Lepidoptera is shown (Additional file 15).

CXE/CCEs display robust expression patterns in the tissues examined with ranges of abundance estimation similar to other gene families described in this report (Additional file 16). In male and female antennae, proboscis and body carcass, but not the brain, SlitCXE2 was among the top three most abundantly expressed CXEs/CCEs. With the exception of SlitCXE25, all previously described antennal CXEs displayed FPKM abundance estimates greater than one in both male and female antennae.

#### Cytochrome P450 proteins

A total of 84 CYP gene transcripts have been identified across both the male and female transcriptomes, including all 41 previously annotated transcripts [29, 36], and 43 novel transcripts (Additional file 17). Complete ORFs are predicted for all SlitCYPs previously predicted as incomplete (SlitCYP301A1, 304F4, 315A1, 341B3, 354A9, 9A51, 9A52). Novel sequences have been named according to the P450 Gene Family Nomenclature Committee (Dr. D. Nelson, University of Tennessee Health Science Center, Memphis, TN, USA). In sum, complete ORFs are predicted for 69 SlitCYP gene transcripts. A phylogenetic tree indicates evolutionary relationships between *S. littoralis* CYPs and those from *D. melanogaster* and *B. mori* (Additional file 18).

CYPs display robust and diverse expression patterns in the tissues examined with ranges of abundance estimation similar to other gene families described in this report (Additional files 6 and 19).

#### SNMP proteins

Both of the previously reported SlitSNMPs, SlitSNMP1, and SlitSNMP2 [25, 26] were identified in both the male and female transcriptomes (Additional file 20). Both gene transcripts were previously reported with complete ORF predictions, and complete ORFs matching those predictions were identified here; SlitSNMP1 and SlitSNMP2 ORFs identified in the transcripts here display 97.81 and 99.39 percent identity to the previously reported sequences at the nucleotide level.

SlitSNMP1 and SlitSNMP2 present similar expression patterns in both males and females (Additional file 6). SlitSNMP1 displayed relatively higher expression in antennae (3.85 x 10^2^ FPKM in male, 1.53 x 10^2^ in female) and relatively lower expression in proboscis (1.2 FPKM in male, 1.05 FPKM in female) and brain (2.48 FPKM in male, 0.8 FPKM in female). SlitSNMP2 displayed relatively moderate to high expression in antenna (1.9 x 10^3^ FPKM in male, 1.33 x 10^3^ FPKM in female), proboscis (1.17 x 10^2^ FPKM in male, 1.49 x 10^2^ FPKM in female) and brain (15.5 FPKM in male, 12.1 FPKM in female). RT-PCR assays of SlitSNMP1 and SlitSNMP2 expression in antennae, proboscis, and brain are consistent with these observations (Fig. 3).

## Discussion

We used Illumina-based RNA Sequencing methodology to provide a expanded picture of the expression patterns of several *S. littoralis* gene families involved in chemosensory processes, namely ORs, GRs, IRs, OBPs, CSPs, CXE/CCEs, CYPs, and SNMPs. Furthermore, we report the first expression abundance estimates for members of these gene families in this species. In total, 306 gene transcripts have been annotated, including 114 novel sequences. Of the 192 previously annotated genes from these families, all were present in the transcriptomes of this study except for one GR and one OBP, while eight previously reported sequences from these families were flagged as mis-annotations for various reasons. Of 54 previously annotated genes from these families that were reported with incomplete ORFs, complete ORFs have now been predicted for 85% of them (n=46). Likewise, complete ORFs are predicted for 86% (n=264) of all of the gene transcripts characterized in this study. Nearly all of the annotated transcripts were identified in the male (n=290) and female (n=293) transcriptomes, providing high confidence in the accuracy of the sequence information for the transcripts being studied.

### ORs

Sixty ORs are now annotated for *S. littoralis.* Recently, several reports on genomic studies of the odorant receptors in lepidopteran species have provided a better range approximation on the number of ORs per species within this insect order: 95 ORs in *P. xylostella* [37, 38], 70 ORs in *E. postvittana* [39], 71 ORs in *B. mori* [40, 41] , 73 ORs in *M. sexta* [42, 43], 64 ORs in *D. plexippus* [38, 44] and 74 ORs in *H. melpomene* [45]. These values suggest that we have likely identified close to the full repertoire of ORs in this species.

A novel OR, SlitOR56, has been identified that clusters phylogenetically with other *S. littoralis* PRs [46, 47]. In males of other moth species, it has been observed that putative PRs may be the most highly expressed ORs in the antennae [48]. However, we observed that two receptors that cluster outside of the PR sub-family, SlitOR5 and SlitOR38, were among the most highly expressed ORs in the male antennae. These findings are similar to those in another report showing relatively highest expression of non-PR subfamily ORs in *E. postvitanna* male antennae (namely EposOR30/OR34) [39]. Interestingly, SlitOR5/OR38 and EposOR30/OR34 cluster together in the same subfamily, along with ORs that display sex-biased expression from *B. mori* (BmorOR30)[40] and *C. pomonella* (CpomOR30/OR31/OR41) [48]. High expression and/or sex-biased expression of ORs from this sub-family hint at essential roles for these genes in sexual communication; to date, however, efforts to functionally characterize the response profiles of ORs from this clade have not been reported.

Consistent with a previous report [25], we have identified OR expression in the proboscis of male as well as female *S. littoralis*, with RNA-Seq and PCR confirmation of expression of SlitOrco, SlitOR14, and SlitOR25. SlitOR14 has been determined to be strongly responsive to phenylacetaldehyde [17], which is known to be a floral released volatile compound [49, 50]. Based on our observations, it is hypothesized that olfactory detection of floral volatiles at close range may influence feeding behavior; furthermore, we can now implicate a candidate receptor governing the recently described proboscis probing behavior in the hawkmoth, *Manduca sexta* [51]; SlitOR14 is orthologous to MsexOR67 [42], and the two receptors may be governing similar behaviors in each species. Given that SlitOR14 is also expressed in the antennae, this OR may also contribute to mediating foraging behaviour, as it has also been shown that moths have an innate preference for floral volatiles [20, 52].

We additionally observed expression of several SlitORs in male and female brain, including SlitOrco, SlitOR14, and SlitOR25. Similar observations in this species were previously reported [[25], see Fig. 6]. As with SlitOR14, SlitOR25 responds to phenolic aromatic compounds, with acetophenone identified as the best-known ligand [17]. It has previously been reported that insect GRs are expressed in the brain, namely a fructose receptor [9] that is involved in internal nutrient sensing. It may similarly be hypothesized that ORs expressed in insect brain could function in the internal detection of neural substrates that have chemical structures similar to the aromatic compounds that these ORs detect from the environment when expressed in the antennae. A recent report indicates expression of ORs in male and female brain of the mosquito, *Aedes aegypti* [53], suggesting that expression of ORs in the brain may be broadly conserved across insect orders.

### GRs

A total of 17 putative GRs have now been annotated in *S. littoralis,* including the first description of putative bitter-compound GRs in this species. This number is far lower than what has been described in other Lepidoptera from genome analyses. Typically, 45-70 GRs are predicted (see *M. sexta* [42] and *H. melpomene* [45]), but even more (up to 200) in polyphagous species (see *Helicoverpa armigera*, [54] and *S. frugiperda* [30]). The dearth of GRs identified in this report may be reflective of the fact that we did not examine specific tissues where GRs are expected to be enriched, such as larval legs and mouthparts or adult legs and ovipositors.

Similar to *B. mori* [55], three putative CO2 receptors have been identified. Carbon dioxide has been shown to be a prominent floral cue used by moths to detect floral food source [56], with CO2 detector neurons well characterized on the moth labial palp appendage [57]. While we did not examine gene expression in the labial palps, our expression data on GR expression in the proboscis shows two of the three putative CO_2_ GRs to be expressed in both males and females, namely, SlitGR2 and SlitGR3. In the antennae of both males and females, SlitGR2 and SlitGR3 are the most highly expressed GRs; this expression pattern is consistent with a previous report showing their expression in the antennae of males and females [27]. In light of these results and a recent report on *Spodoptera exigua*, comparing expression levels of putative CO_2_ receptors in antennae, proboscis and labial palps [58], further work is required to define the molecular mechanisms and functional role of CO2 detection in *S. littoralis*, and indeed, other Lepidoptera.

Similar to other moths, a total of five putative sugar-compound receptors have been identified; in this highly conserved GR sub-family, five receptors were identified in *B. mori* [40], and *P. xylostella* [37, 38] and six were identified in *H. melpomene* [45], while 11 were identified in *D. plexippus* [41, 44].

Putative sugar-detecting GR transcripts were detected in male and female antennae and proboscis of *S. littoralis*. A role for the insect antenna in contact chemoreception is well understood [59]. A recent report has described *S. littoralis* antennal sensitivity to sugars sucrose, glucose and fructose [60]; here we provide a more detailed blueprint of the molecular bases for the detection of sugars by the antennae of *S. littoralis.*

While we did not detect the previously reported fructose sub-family receptor, SlitGR10 (formerly SlitGR4) in our transcriptomes, we did identify a novel gene transcript that clusters with other receptors in this clade, SlitGR9. Expression of this receptor was detected in male and female antennae and brain tissues, but not in proboscis and body carcass. A *B. mori* orthologue (BmorGR9), which is 63% identical to SlitGR9, has been shown to be responsive to fructose [61], suggesting that SlitGR9, if it maintains similar function, may be responsible for antennal fructose detection [60]. Similarly, SlitGR9 may also mediate internal nutrient sensing in the brain, as has been shown for the brain-expressed *D. melanogaster* fructose-clade receptor DmelGR43a [9].

A recent report characterized contact chemosensory sensilla on *S. littoralis* ovipositors [62], demonstrating that this sensilla type is innervated by neurons that are receptive to sugar (sucrose and fructose) and bitter compounds. In our female body sample, low expression of the fructose sub-family receptor, SlitGR9, was detected. However, further evaluation of the expression of receptors specifically in ovipositor tissue is required to assess the molecular mechanisms underlying gustatory function in this tissue.

A total of seven putative bitter-compound receptor GRs have been identified in this report, representing the first identification of putative GRs from this clade in *S. littoralis*.

However, recent studies have suggested an expansion of the bitter-compound GR clade in related polyphagous moths [30, 54], supported by both transcriptomic and genomic data. Since *S. littoralis* is highly polyphagous, one would hypothesize a large number of so-called bitter-compound GRs to be expressed in this species, though it remains to be seen if that will be true.

The putative bitter-compound GRs identified here displayed different expression patterns across all tissues examined in both male and females, with a single receptor candidate, SlitGR230, having been detected in antennal, proboscis and brain tissues. A recent report on GRs in the noctuid moth, *H. armigera*, described novel classes of intronless insect GRs, including functionally characterized receptors with shorter ORFs (ca. 200-350 amino acids) [54]. Similar to SlitGR230, two GRs from this report displayed broad tissue expression.

Five of these seven SlitGR candidates clustered in different bitter-compound receptor sub-families, with only one set of two genes clustering together as potential paralogues. Species-specific expansions of different putative bitter-compound receptor lineages have been noted as a more prominent feature of GRs in Lepidoptera compared to most OR sub-families [38, 41]; in *S. littoralis*, however, identification of insufficient numbers of putative bitter-compound GRs precludes characterization of this phenomenon.

### IRs

A total of 17 IRs were identified in this study, building on the gene transcripts previously identified [27, 32], and comparable to the 20-21 antennal IRs identified in *S. exigua* [58]*, C. pomonella* [48] and *M. sexta* [42]. Similar to previous reports [27, 32] all identified SlitIRs displayed adult male and female antennal expression here, consistent with their categorization as antennal IRs. The putative IR co-receptors displayed the highest expression estimates in both male and female antenna, similar to findings in other studies [39, 48, 58] and consistent with their hypothesized role as co-receptors with broader expression patterns compared to putative tuning IRs [63, 64]. In the tissues examined, the putative IR co-receptors displayed broad expression patterns, with SlitIR8a expressed in antennae, and SlitIR25a and SlitIR76b expressed in all tissues, similar to previous findings [32]. On the contrary, among the putative tuning IRs, FPKM expression values above one outside of the antennae were not consistently observed.

### OBPs and CSPs

In *S. littoralis,* both OBPs and CSPs display broad and diverse expression patterns in all tissues examined here, suggesting diverse roles for these proteins related to chemosensory and non-chemosensory processes. Indeed, these findings are consistent with other reports on expression patterns of both OBPs and CSPs [65–67]. Furthermore, it has been demonstrated that CSPs can function in processes as diverse as chemosensory ligand binding of semiochemicals, such as pheromones, and tissue development and regeneration (summarized in [68]).

Among all of the gene families examined in this report, annotated OBPs claimed the highest percentage share of total summed FPKM expression values in both male and female antennae, at 17.7% in males and 11.0% in females. Previous reports have similarly shown that OBPs are among the most abundantly expressed genes in insect antennal tissues from moth [67] to mosquito [69].

The pheromone-binding protein (PBP) sub-family of OBPs contains proteins known to bind moth pheromone components and facilitate PR activation [70, 71]. Consistent with a prominent role for the moth male antennae in the detection of female-produced sex pheromone, SlitPBP1 is observed to be the most highly expressed OBP in male antennae. SlitPBP1 was also observed to be the second most highly expressed OBP in the female antennae. We confirm that putative, orphan pheromone receptors SlitOR11 and SlitOR16 are expressed in the female antennae [27], and female antennae have been shown, physiologically, to detect female-produced pheromone components [14, 15]. Intriguingly, SlitPBP1, 2 & 3, which are also expressed in larval antennal tissue [27, 72], also display varying expression patterns in male and female proboscis and brain tissues, suggesting broader physiological roles than their “pheromone binding” designation implies. Indeed, it has been previously shown that PBP proteins from the silkworm moth, *B. mori* can bind and interact with non-pheromonal compounds [73].

Compared to OBPs, CSPs claimed the highest percentage share of total summed FPKM expression values, among the gene families studied here, in the proboscis, with 9.1% in females and 5.2% in males. It has recently been reported that two CSPs from the butterfly, *Vanessa gonerilla* comprise greater than 50% of the total content of the larval mandibular gland proteome [74]. Similarly here, two CSPs, SlitCSP2 and SlitCSP8 comprise 87.6% and 80.0% of the total summed CSP FPKM expression values in the proboscis of females and males respectively. Interestingly, SlitCSP2 and SlitCSP8 are among the top five most abundantly expressed CSPs in all tissues examined suggesting critical functional roles for these proteins beyond chemosensory processes.

### CXEs and P450s

Similar to the OBPs and CSPs, the CXE/CCE and CYPs displayed diverse expression patterns in all tissues examined in both males and females. Among possible ODE families, we focused on these gene families to provide an extension of previous studies on antennal expressed genes that may function as ODEs; indeed, it has been reported that some SlitCXEs bind to and modify odorants [75, 76]; SlitCXE7 was previously reported to have functional activity on both pheromone and plant volatile compounds with antennal-specific expression patterns in larval and adult *S. littoralis* [75].

To that end, all previously identified antennal expressed CXEs were found to be expressed in both male and female antennae in this report. Of the 26 novel SlitCCEs identified, 12 displayed antennal expression in males, females or both, bringing the total number of antennal expressed esterases to 42.

## Conclusions

The curation of a broad set of putative chemosensory genes in *S. littoralis* will serve as a useful resource for future transcriptome and genome annotation efforts in *S. littoralis* and closely related insects. A thorough analysis of the expression patterns of known and putative chemosensory genes in male and female chemosensory tissues provides a framework for a better understanding of the molecular mechanisms of olfaction and gustation in *S. littoralis*. Furthermore, the data presented here, when compared to gene expression studies of other insects, may provide evolutionary insights with regards to conserved and divergent molecular function and physiological/behavioral processes. The observation of gene expression of known chemosensory genes in non-sensory tissues, such as the brain, suggests novel functions for these genes in non-chemosensory contexts. When coupled with functional data, such as the deorphanization of ORs, the gene expression data can facilitate hypothesis generation, serving as a substrate for future studies. For example, the expression in the proboscis of an OR that detects a floral odorant suggests that this OR may underlie floral/nectar feeding behavior as observed in a closely related moth.

## Methods

### Insect Rearing

Cotton leafworm (*S. littoralis*) was obtained from cotton fields (El-Shatby, Egypt) in 2010 and reared on a standard semi-artificial potato-based diet; the colony has been refreshed with new wild-collected individuals approximately every 6 months.

All insects were maintained under a 16L:8D photoperiod, at 23 +/- 1 °C and 50–60% relative humidity, adults had access to water and sugar solution. 3 day-old male and female moths were used for dissections and RNA extractions.

### Tissue Dissections/RNA Extractions

For RNA-Seq samples,, antennae, brain, and proboscis were dissected from 50-60 male or female moths per sample. For RT-PCR samples, antennae, brain, and proboscis were dissected from 30 male or female virgin moths per sample. All tissue samples were dissected into 500 microliters of Trizol reagent (Life Technologies, Carlsbad, CA, USA) on ice. Dissections were made from 3-4 hours into the scotophase in a dark environment with dimmed background lighting. Antennae and proboscis were separated from the head of living organisms. For brain dissections, after removal of antennae and proboscis, the head was removed into 1X Phosphate Buffer Saline (PBS) and brain tissue was removed from the head capsule. For each body carcass sample, three individuals were used directly after removal of the head. After dissections, all samples were snap frozen on dry ice or liquid nitrogen and stored at -80°C until all samples were ready for processing.

Total RNA was extracted and purified with a combined approach of Trizol-based extraction followed by RNeasy Mini spin column purification (Qiagen, Venlo, Netherlands), as previously described [48]. RNA was eluted with 40 microliters of supplied RNase Free water and immediately assayed for quality and concentration with a Nanodrop 1000 spectrophotometer (Thermo Scientific, Waltham, MA, USA). Quality and quantity parameters for each sample are provided in Additional file 1.

### RNA Sequencing

Pure total RNA samples from both males and females (three antennal, three brain, two body carcass, and one proboscis) were prepared and sequenced commercially at the Beijing Genomics Institute (BGI) sequencing facility in Hong Kong (BGI Hong Kong Co.) using standard protocol (Additional file 21, Section 1). Through Illumina HiSeqTM 2000 sequencing, paired-end reads (90PE) were generated and saved in FASTQ format [77]. Low quality reads that did not meet any of the following criteria were removed with proprietary BGI software: reads with sequenced adaptors reads with greater than 5% unknown nucleotides and reads that have greater than 50% of nucleotide bases with PHRED quality scores [78, 79] less than 10.

### Bioinformatic Pipelines – Pre-Assembly, Assembly, Post Assembly

Initial quality control measures for obtained raw reads were undertaken prior to assembly (Additional file 21, Section 2). Trimmed, filtered reads were assembled, *de novo,* into two transcriptomes, one compiled from all male sample FASTQ files and the other compiled from all female sample FASTQ files. Transcriptome assemblies were carried out with Trinity software (release version 2013-02-05) [80]. The Trinity Perl script was executed with default parameters. In order to facilitate unambiguous read mapping of individual sample reads back to unique locations on the assembled transcriptome sequences for downstream quantitative analyses, the software cd-hit-est (version 4.5.4-2011-03-07) was used to identify and remove redundant sequences that share 98% or greater identity with other sequences [81]. The male and female transcriptome Trinity.fasta files were independently used as input, program parameters -c 0.98 -n 8 were specified, resulting in two separate output files. In cases where sequences shared greater than 98% identity but were of different sizes, the largest of the sequences were retained in the fasta file.

To assess the completeness of the transcriptomes, an Arthropoda BUSCO database, consisting of 1066 core genes that are highly conserved single-copy orthologues [82, 83], was used to query the Trinity.fasta transcriptomes, For this process, the gVolante web server (https://gvolante.riken.jp/) was utilized with the following parameters: min_length_of_seq_stats: 1, assembly_type: trans, Program: BUSCO_v2/v3, selected reference_gene_set: Arthropoda.

### Chemosensory Gene Annotation Procedures

Text files were compiled in fasta format for the different chemosensory gene families, with protein sequences included for previously characterized *S. littoralis* genes. OR, GR, OBP, and CSP sequences were taken from the supplementary materials of previous reports [26, 27]. IR [27, 32], CXE [28] and CYP [29] sequences were also obtained.

Blast nucleotide databases were created from The Trinity.fasta files and were queried by the protein sequence fasta files for each of the chemosensory gene families. For this procedure, Blast version 2.2.24+ was used to perform a tblastn query and a minimum e-score threshold of 1e-05 was required for hits; additional parameters included num_descriptions 50 and -num_threads 8; blast output files were generated with output format six [84]. For each of the previously annotated chemosensory genes, the top blast hit transcript cluster was manually extracted from the Trinity.fasta file. Nucleotide sequences were translated into protein sequence with the ExPASy web Translate tool [85], and the protein sequences were aligned to reference annotations with the ClustalOMEGA web tool (http://www.ebi.ac.uk/Tools/msa/clustalo/) [86].

Novel chemosensory genes were identified and annotated in an iterative search process. First, all sequences that were reported in the original blast searches that did not correlate to previously annotated genes were examined. These sequences were used as input for a web-based blastx query in order to verify homology to putative chemosensory genes and identify ORF orientation. In order to reduce the possibility of mis-annotating two uncoupled fragments of the same gene as distinct genes, only sequences with ORFs greater the 50% of the average length of a complete ORF in a given gene family (OR = 406 amino acids (aa), GR= 447 aa, IR= 676 aa, OBP = 178 aa, CSP= 132 aa, CXE= 563 aa, CYP= 510 aa) were included for further analysis. The protein sequences of novel gene candidates identified as described here were incorporated into fasta files for each gene family, and an additional tblastn query was performed against the Trinity.fasta nucleotide databases in order to determine if any further gene candidates would be identified.

### Quantitative Analysis of Chemosensory Gene Expression Levels with RSEM

Read mapping of individual sample reads to the *de novo* generated transcriptomes and subsequent expression level abundance estimations were carried out, as described [87] with the Trinity Perl script align_and_estimate_abundance.pl in the r20140717 release version of Trinity, using version 1.2.12 of RSEM [88], version 0.12.6 of Bowtie [89] and version 0.1.19 of samtools [90]. Subsequent to manual editing of selected clusters of annotated chemosensory genes to control for bioinformatic processing artifacts in the transcriptome (Additional file 21, Section 3), the cd-hit-est-modified Trinity.fasta files were used as reference transcripts input and the trimmed fastq reads described above were used as mapping input. The genes_trans_map file (described in Additional file 21, Section 3) was used as input for determining FPKM values [88] of each subcomponent gene transcript, as a basis for estimation of gene expression abundance levels.

### Phylogenetic Trees of Chemosensory Gene Families

For the qualitative report of gene family transcripts, published sets of genes from different species were used for comparison with our data. *S. littoralis* ORs were compared to sequences from *Bombyx mori* [40, 41], *Epiphyias postvitanna* [39] and *Heliconius melpomene* [45]. *S. littoralis* GRs were compared to sequences from *B. mori* [55], and *Helicoverpa armigera* [54]*. S. littoralis* IRs were compared to sequences from *B. mori* and *D. melanogaster* [64], *Manduca sexta* [42], *Cydia pomonella* [91] and *Danaus plexipus* [41, 44]. *S. littoralis* OBPs were compared to sequences from *B. mori* [65] and *H. melpomene* [45]. *S. littoralis* CSPs were compared to sequences from *B. mori* [66] and *H. melpomene* [45]. *S. littoralis* CXEs were compared to sequences from *B. mori* [92] and *E. postvitanna* [39]. *S. littoralis* P450s were compared to sequences from *B. mori* [93] and *D. melanogaster* [94].

Amino acid sequences for each gene family were aligned using MAFFT online version 7.220 (https://mafft.cbrc.jp/alignment/server/) through the FFT-NS-i iterative refinement method, with JTT200 scoring matrix, “leave gappy regions” set, and other default parameters [95]. Aligned sequences were used to build the phylogenies with MEGA7 software [96] in command line, with the following parameters: Maximum Likelihood Tree Method with the JTT-F’ model, uniform rates, use all sites, nearest neighbor interchange heuristic method, very strong branch swap filter and default automatic NJ/BioNJ initial tree. The bootstrap consensus of each phylogenetic tree was inferred from 600 replicates. Consensus Newick format trees were compiled with MEGA6.06 software [96] and edited with Adobe Illustrator.

### Heatmap Presentation of Gene Expression

Heatmap plots were generated for the binary logarithm of raw FPKM-plus-1 values. These plots were made using the conditional formatting function in Microsoft Excel, with a three-color scale. For each plot, the minimum value was set to number type, with a value of one, and displayed as black; midpoint was set to percentile type, with a value of 75, and displayed as dark color; maximum was set to highest value type, and displayed as bright color. For all gene families, the range was specified for each tissue type independently, such that the color gradient was set based upon the highest FPKM values within each tissue, not across all tissues.

### RT-PCR Assay of Gene Expression

cDNA was generated with input of 1 μg of total RNA using the RevertAid Minus H first strand cDNA synthesis kit (Thermo Fisher Scientific, Waltham, MA, USA) according to the manufacturer's protocol. PCR assays were performed, with the Dream Taq Green master mix system (Thermo Fisher Scientific), on cDNA from single biological samples of virgin male and female antennae, proboscis and brain. Specific primer pairs (Additional file 22) were used for each chemosensory gene and the ribosomal protein, rpL8, was used as a positive control. For all PCR assays, thermocycling conditions were used for 35 cycles of: 30s at 95°C, 30s at 55°C and 1m at 72°C. PCR reactions were loaded on 1.5% agarose gels loaded with Gel Red stain (Biotium Inc., Fremont, CA, USA), and after electrophoresis, were visualized under UV light. Template-free and No-RT negative controls were also included for each primer pair and tissue type, respectively. Additional files are included for uncropped gels with experimental assays and no template controls (Additional file 23) and NORT assays (Additional file 24).

## Additional files

Additional file 1: Metrics on Biological Samples. Includes input RNA concentration, purity, and also number of quality controlled and filtered read pairs per sequenced samples. (XLSX 39 kb)

Additional file 2: Bioinformatic Information for Male and Female Transcriptomes. Contains basic information on the transcriptomes at various stages of processing, including total number of sequences, mean sequence length, N50 and other parameters. (XLSX 35 kb)

Additional file 3: Novel Candidate Chemosensory Gene Informatics. Includes annotation name, ORF size, best blast hit and other parameters. (DOCX 178 kb)

Additional file 4: Estimated Transcript Abundance Peak Values by Gene Family, Tissue and Sex, Fragments per Kilobase per Million (FPKM). (DOCX 15 kb)

Additional file 5: *S. littoralis* OR protein sequences. Contains updated amino acid sequences for all annotated *S. littoralis* odorant receptors. (TXT 24 kb)

Additional file 6: Candidate Chemosensory Gene Abundance Estimates. Contains “FPKM” and “estimated mapped reads” values for candidate chemosensory gene transcripts, sorted by chemosensory gene family, sex and tissue type. (XLSX 47 kb)

Additional file 7: *S. littoralis* GR protein sequences. Contains updated amino acid sequences for all annotated *S. littoralis* gustatory receptors. (TXT 6 kb)

Additional file 8: Heat-plot of relative expression values for *S. littoralis* GRs. Estimation of abundance values determined by read mapping. Black indicates low/no expression, dark colors indicate low/moderate expression, bright colors indicate moderate/high expression. Color plots represent binary log of FPKM plus one for each gene (See Additional file 6 for raw data). Color scales for each tissue type are independent of other tissue types. Range of values for Male Antenna: 0 – 3.28; Male Brain: 0 – 3.37; Male Body: 0 – 3.65; Male Proboscis: 0 – 4.64; Female Antennae: 0 – 3.58; Female Brain: 0 – 3.16; Female Body: 0 – 1.44; Female Proboscis: 0 – 5.97. (TIF 599 kb)

Additional file 9: *S. littoralis* IR protein sequences. Contains updated amino acid sequences for all annotated *S. littoralis* ionotropic receptors. (TXT 11 kb)

Additional file 10: *S. littoralis* OBP protein sequences. Contains updated amino acid sequences for all annotated *S. littoralis* odorant binding proteins. (TXT 9 kb)

Additional file 11: Maximum likelihood cladogram of candidate SlitOBP sequences with other lepidopteran OBP sequences. Unrooted. Includes sequences from *S. littoralis* (Slit), *Heliconius melpomene* (Hmel) and *Bombyx mori* (Bmor). Branches containing “Plus-C” subfamily OBPs are colored green; branches containing “Minus-C” subfamily OBPs are colored blue; branches containing putative pheromone binding proteins (PBPs) and general odorant binding proteins (GOBPs) are colored orange; *S. littoralis* OBPs are indicated with a larger bold font, and novel *S. littoralis* OBPs are marked with a “•”. Node support was assessed with 600 bootstrap replicates and values greater than 70% are shown. (PDF 368 kb)

Additional file 12: *S. littoralis* CSP protein sequences. Contains updated amino acid sequences for all annotated *S. littoralis* chemosensory proteins. (TXT 2 kb)

Additional file 13: Maximum likelihood cladogram of candidate SlitCSP sequences with other lepidopteran CSP sequences. Unrooted. Includes sequences from *S. littoralis* (Slit), *Heliconius melpomene* (Hmel) and *Bombyx mori* (Bmor). *S. littoralis* CSPs are indicated with a larger bold font, and novel *S. littoralis* CSP is marked with a “•”. Node support was assessed with 600 bootstrap replicates and values greater than 70% are shown. (PDF 283 kb)

Additional file 14: *S. littoralis* CXE/CCE protein sequences. Contains updated amino acid sequences for all annotated *S. littoralis* carboxy/cholinesterase proteins. (TXT 29 kb)

Additional file 15: Maximum likelihood cladogram of candidate SlitCXE sequences with other lepidopteran CXE/CCE sequences. Unrooted. Includes sequences from *S. littoralis* (Sl), *E. postvittana* (Epos) and *Bombyx mori* (Bm). *S. littoralis* CXE/CCEs are indicated with a larger bold font, and novel *S. littoralis* CXE/CCEs is marked with a “•”. Node support was assessed with 600 bootstrap replicates and values greater than 70% are shown. (PDF 386 kb)

Additional file 16: Heat-plot of relative expression values for *S. littoralis* CXE/CCEs. Estimation of abundance values determined by read mapping. Black indicates low/no expression, dark colors indicate low/moderate expression, bright colors indicate moderate/high expression. Color plots represent binary log of FPKM plus one for each gene (See Additional file 6 for raw data). Color scales for each tissue type are independent of other tissue types. “N.F.” indicates that gene transcripts were not found in respective transcriptome. “N/A” indicates that unique gene model could not be resolved for gene transcripts in respective transcriptome due to co-assembly of highly similar gene models. Range of values for Male Antenna: 0 – 9.53; Male Brain: 0 – 5.75; Male Body: 0 – 9.17; Male Proboscis: 0 – 8.71; Female Antennae: 0 – 8.82; Female Brain: 0 – 7.06; Female Body: 0 – 8.55; Female Proboscis: 0 – 10.11. (TIF 2410 kb)

Additional file 17: *S. littoralis* CYP protein sequences. Contains updated amino acid sequences for all annotated *S. littoralis* Cytochrome p450 proteins. (TXT 41 kb)

Additional file 18 Maximum likelihood phylogenetic tree of candidate SlitCYPs sequences with other insect CYP sequences. Unrooted. Includes sequences from *S. littoralis* (Sl), *D. melanogaster* (Dmel) and *B. mori* (Bmor). Branches containing mitochondrial clan CYPs are colored red. *S. littoralis* CYPs are indicated with a larger bold font, and novel *S. littoralis* CYPs are marked with a “•”. Node support was assessed with 600 bootstrap replicates and values greater than 70% are shown. (PDF 532 kb)

Additional file 19: Heat-plot of relative expression values for SlitCYPs. Estimation of abundance values determined by read mapping. Black indicates low/no expression, dark colors indicate low/moderate expression, bright colors indicate moderate/high expression. Color plots represent binary log of FPKM plus one for each gene (See Additional file 6 for raw data). Color scales for each tissue type are independent of other tissue types. “N.F.” indicates that gene transcripts were not found in respective transcriptome. “N/A” indicates that unique gene model could not be resolved for gene transcripts in respective transcriptome due to co-assembly of highly similar gene models. Range of values for Male Antenna: 0 – 11.28; Male Brain: 0 – 9.83; Male Body: 0 – 9.73; Male Proboscis: 0 – 11.61; Female Antennae: 0 – 13.14; Female Brain: 0 – 7.87; Female Body: 0 – 8.15; Female Proboscis: 0 – 12.11. (TIF 3197 kb)

Additional file 20: *S. littoralis* SNMP protein sequences. Contains updated amino acid sequences for all annotated *S. littoralis* sensory neuron membrane proteins. (TXT 1 kb)

Additional file 21: supplemental materials and methods. (DOCX 22 kb)

Additional file 22: PCR primer sequences and expected fragment sizes. (XLSX 10 kb)

Additional file 23: uncropped PCR gels, including all experimental assays and no template-controls. For all gels, 1kb Gene Ruler ladder (Thermo Fisher Scientific) was used . MVA – male virgin antennae, FVA – female virgin antennae, MVPR – male virgin proboscis, FVPR, female virgin proboscis, MVBR – male virgin brain, FVBR – female virgin brain. On some gels, part of the gel space was used for experiments unrelated to this manuscript; those sections are indicated as “not applicable.” (PDF 5006 kb)

Additional file 24: uncropped PCR gels, including all no-RT controls. For all gels, 1kb Gene Ruler ladder (Thermo Fisher Scientific) was used. MVA – male virgin antennae, FVA – female virgin antennae, MVPR – male virgin proboscis, FVPR, female virgin proboscis, MVBR – male virgin brain, FVBR – female virgin brain, N/A – not applicable. On some gels, part of the gel space was used for experiments unrelated to this manuscript; those sections are indicated as “not applicable.” (PDF 3777 kb)

## Data Availability

All data generated and analyzed in this study are included in the published article and its additional information files. Transcriptome raw reads sequence data are available through the NCBI Sequence Read Archive, (BioProject: PRJNA312160; SRA Accession Number SRP104658; SRX2750974- SRX2750991 (18 samples). Chemosensory Gene Transcript sequences identified from the male and female *Spodoptera littoralis* transcriptome assemblies are available through NCBI. All sequences are included in a Transcriptome Shotgun Assembly project that has been deposited at DDBJ/EMBL/GenBank. All novel coding protein sequences are available as part of the supporting data.

## Abbreviations

CCE: Carboxyl/Cholinesterase
CO2: Carbon Dioxide
CSP: Chemosensory Protein
CXE: Carboxyl Esterase
CYP: Cytochrome P450
FPKM: Fragments per Kilobase per Million
GR: Gustatory Receptor
IR: Ionotropic Receptor
OBP: Odorant Binding Protein
ODE: Odorant Degrading Enzyme
OR: Odorant Receptor
ORCO: Odorant Receptor Co-Receptor
ORF: Open Reading Frame
PBP: Pheromone Binding Protein
PR: Pheromone Receptor
RNA-Seq: RNA Sequencing
SNMP: Sensory Neuron Membrane Protein

## References

[1] Hansson BS, Stensmyr MC (2011). Evolution of insect olfaction. Neuron.

[2] Schoonhoven LM, van Loon JJA, Dicke M (2005). Insect-plant biology.

[3] Bruce TJ, Pickett JA (2011). Perception of plant volatile blends by herbivorous insects--finding the right mix. Phytochemistry.

[4] Ozaki K, Ryuda M, Yamada A, Utoguchi A, Ishimoto H, Calas D, Marion-Poll F, Tanimura T, Yoshikawa H (2011). A gustatory receptor involved in host plant recognition for oviposition of a swallowtail butterfly. Nat Commun.

[5] Leal WS (2013). Odorant reception in insects: roles of receptors, binding proteins, and degrading enzymes. Annu Rev Entomol.

[6] Suh E, Bohbot J, Zwiebel LJ (2014). Peripheral olfactory signaling in insects. Curr Opin Insect Sci.

[7] Kitabayashi AN, Arai T, Kubo T, Natori S (1998). Molecular cloning of cDNA for p10, a novel protein that increases in the regenerating legs of Periplaneta americana (American cockroach). Insect Biochem Mol Biol.

[8] Wanner KW, Isman MB, Feng Q, Plettner E, Theilmann DA (2005). Developmental expression patterns of four chemosensory protein genes from the Eastern spruce budworm, Chroistoneura fumiferana. Insect Mol Biol.

[9] Miyamoto T, Slone J, Song X, Amrein H (2012). A fructose receptor functions as a nutrient sensor in the Drosophila brain. Cell.

[10] Pitts RJ, Liu C, Zhou X, Malpartida JC, Zwiebel LJ (2014). Odorant receptor-mediated sperm activation in disease vector mosquitoes. Proc Natl Acad Sci U S A.

[11] Karner T, Kellner I, Schultze A, Breer H, Krieger J. Co-expression of six tightly clustered odorant receptor genes in the antennae of the malaria mosquito *Anopheles gambiae*. Front Ecol Evo*l*. 2015;3(26):1–8.

[12] Brown ES, Dewhurst CF (1975). Genus *Spodoptera* (Lepidoptera, Noctuidae) in Africa and near East. Bull Entomol Res.

[13] Lopez-Vaamonde C, Europe DAISIi (2006). Delivering Alien Invasive Species Inventories in Europe: *Spodoptera littoralis*. Fact Sheet.

[14] Anderson P, Hansson BS, Lofqvist J (1995). Plant-Odor-Specific Receptor Neurons on the Antennae of Female and Male *Spodoptera littoralis*. Physiol Entomol.

[15] Binyameen M, Anderson P, Ignell R, Seada MA, Hansson BS, Schlyter F (2012). Spatial organization of antennal olfactory sensory neurons in the female *Spodoptera littoralis* moth: differences in sensitivity and temporal characteristics. Chem Senses.

[16] Ljungberg H, Anderson P, Hansson BS (1993). Physiology and morphology of pheromone-specific sensilla on the antennae of male and female *Spodoptera littoralis* (Lepidoptera, Noctuidae). J Insect Physiol.

[17] de Fouchier A, Walker WB, Montagne N, Steiner C, Binyameen M, Schlyter F, Chertemps T, Maria A, Francois MC, Monsempes C (2017). Functional evolution of Lepidoptera olfactory receptors revealed by deorphanization of a moth repertoire. Nat Commun.

[18] Party V, Hanot C, Said I, Rochat D, Renou M (2009). Plant terpenes affect intensity and temporal parameters of pheromone detection in a moth. Chem Senses.

[19] Martel V, Anderson P, Hansson BS, Schlyter F (2009). Peripheral modulation of olfaction by physiological state in the Egyptian leaf worm *Spodoptera littoralis* (Lepidoptera: Noctuidae). J Insect Physiol.

[20] Saveer AM, Kromann SH, Birgersson G, Bengtsson M, Lindblom T, Balkenius A, Hansson BS, Witzgall P, Becher PG, Ignell R (2012). Floral to green: mating switches moth olfactory coding and preference. Proc Biol Sci.

[21] Proffit M, Khallaf MA, Carrasco D, Larsson MC, Anderson P (2015). ‘Do you remember the first time?’ Host plant preference in a moth is modulated by experiences during larval feeding and adult mating. Ecol Lett.

[22] Thoming G, Larsson MC, Hansson BS, Anderson P (2013). Comparison of plant preference hierarchies of male and female moths and the impact of larval rearing hosts. Ecology.

[23] Lhomme P, Carrasco D, Larsson M, Hansson B, Anderson P. A context-dependent induction of natal habitat preference in a generalist herbivore insect. Behav Ecol. 2017;8:1–8.

[24] Zakir A, Bengtsson M, Sadek MM, Hansson BS, Witzgall P, Anderson P (2013). Specific response to herbivore-induced de novo synthesized plant volatiles provides reliable information for host plant selection in a moth. J Exp Biol.

[25] Legeai F, Malpel S, Montagné N, Monsempes C, Cousserans F, Merlin C, Francois MC, Maibeche-Coisne M, Gavory F, Poulain J (2011). An Expressed Sequence Tag collection from the male antennae of the Noctuid moth *Spodoptera littoralis*: a resource for olfactory and pheromone detection research. BMC Genomics.

[26] Jacquin-Joly E, Legeai F, Montagné N, Monsempes C, Francois MC, Poulain J, Gavory F, Walker WB, Hansson BS, Larsson MC (2012). Candidate chemosensory Genes in Female Antennae of the Noctuid Moth *Spodoptera littoralis*. Int J Biol Sci.

[27] Poivet E, Gallot A, Montagne N, Glaser N, Legeai F, Jacquin-Joly E (2013). A comparison of the olfactory gene repertoires of adults and larvae in the noctuid moth *Spodoptera littoralis*. PloS One.

[28] Durand N, Carot-Sans G, Chertemps T, Montagne N, Jacquin-Joly E, Debernard S, Maibeche-Coisne M (2010). A diversity of putative carboxylesterases are expressed in the antennae of the noctuid moth *Spodoptera littoralis*. Insect Mol Biol.

[29] Pottier MA, Bozzolan F, Chertemps T, Jacquin-Joly E, Lalouette L, Siaussat D, Maibeche-Coisne M (2012). Cytochrome P450s and cytochrome P450 reductase in the olfactory organ of the cotton leafworm *Spodoptera littoralis*. Insect Mol Biol.

[30] Gouin A, Bretaudeau A, Nam K, Gimenez S, Aury JM, Duvic B, Hilliou F, Durand N, Montagne N, Darboux I (2017). Two genomes of highly polyphagous lepidopteran pests (Spodoptera frugiperda, Noctuidae) with different host-plant ranges. Sci Rep.

[31] Zhang DD, Löfstedt C. Moth pheromone receptors: gene sequences, function, and evolution. Front Ecol Evol. 2015;3(105):1–10.

[32] Olivier V, Monsempes C, Francois MC, Poivet E, Jacquin-Joly E (2011). Candidate chemosensory ionotropic receptors in a Lepidoptera. Insect Mol Biol.

[33] Durand N, Chertemps T, Maibeche-Coisne M (2012). Antennal carboxylesterases in a moth, structural and functional diversity. Commun Integr Biol.

[34] Merlin C, Rosell G, Carot-Sans G, Francois MC, Bozzolan F, Pelletier J, Jacquin-Joly E, Guerrero A, Maibeche-Coisne M (2007). Antennal esterase cDNAs from two pest moths, Spodoptera littoralis and Sesamia nonagrioides, potentially involved in odourant degradation. Insect Mol Biol.

[35] Teese MG, Campbell PM, Scott C, Gordon KH, Southon A, Hovan D, Robin C, Russell RJ, Oakeshott JG (2010). Gene identification and proteomic analysis of the esterases of the cotton bollworm, *Helicoverpa armigera*. Insect Biochem Mol Biol.

[36] Iga M, Smagghe G (2010). Identification and expression profile of Halloween genes involved in ecdysteroid biosynthesis in *Spodoptera littoralis*. Peptides.

[37] You M, Yue Z, He W, Yang X, Yang G, Xie M, Zhan D, Baxter SW, Vasseur L, Gurr GM (2013). A heterozygous moth genome provides insights into herbivory and detoxification. Nat Genet.

[38] Engsontia P, Sangket U, Chotigeat W, Satasook C (2014). Molecular evolution of the odorant and gustatory receptor genes in lepidopteran insects: implications for their adaptation and speciation. J Mol Evol.

[39] Corcoran JA, Jordan MD, Thrimawithana AH, Crowhurst RN, Newcomb RD (2015). The Peripheral Olfactory Repertoire of the Lightbrown Apple Moth, *Epiphyas postvittana*. PloS One.

[40] Wanner KW, Anderson AR, Trowell SC, Theilmann DA, Robertson HM, Newcomb RD (2007). Female-biased expression of odourant receptor genes in the adult antennae of the silkworm, *Bombyx mori*. Insect Mol Biol.

[41] Briscoe AD, Macias-Munoz A, Kozak KM, Walters JR, Yuan F, Jamie GA, Martin SH, Dasmahapatra KK, Ferguson LC, Mallet J (2013). Female behaviour drives expression and evolution of gustatory receptors in butterflies. PLoS Genet.

[42] Koenig C, Hirsh A, Bucks S, Klinner C, Vogel H, Shukla A, Mansfield JH, Morton B, Hansson BS, Grosse-Wilde E (2015). A reference gene set for chemosensory receptor genes of *Manduca sexta*. Insect Biochem Mol Biol.

[43] Grosse-Wilde E, Kuebler LS, Bucks S, Vogel H, Wicher D, Hansson BS (2011). Antennal transcriptome of *Manduca sexta*. Proc Natl Acad Sci U S A.

[44] Zhan S, Merlin C, Boore JL, Reppert SM (2011). The monarch butterfly genome yields insights into long-distance migration. Cell.

[45] Dasmahapatra KK, Walters JR, Briscoe AD, Davey JW, Whibley A, Nadeau NJ, Zimin AV, Hughes DST, Ferguson LC, Martin SH (2012). Butterfly genome reveals promiscuous exchange of mimicry adaptations among species. Nature.

[46] de Fouchier A, Sun X, Monsempes C, Mirabeau O, Jacquin-Joly E, Montagné N. Evolution of two receptors detecting the same pheromone compound in crop pest moths of the genus *Spodoptera*. Front Ecol Evol. 2015(95):3:1–11.

[47] Montagné N, Chertemps T, Brigaud I, Francois A, Francois MC, de Fouchier A, Lucas P, Larsson MC, Jacquin-Joly E (2012). Functional characterization of a sex pheromone receptor in the pest moth *Spodoptera littoralis* by heterologous expression in Drosophila. Eur J Neurosci.

[48] Walker WB, Gonzalez F, Garczynski SF, Witzgall P (2016). The chemosensory receptors of codling moth *Cydia pomonella* - expression in larvae and adults. Sci Rep.

[49] Bruce TJ, Cork A (2001). Electrophysiological and behavioral responses of female *Helicoverpa armigera* to compounds identified in flowers of African marigold, *Tagetes erecta*. J Chem Ecol.

[50] Cunningham JP, Moore CJ, Zalucki MP, West SA (2004). Learning, odour preference and flower foraging in moths. J Exp Biol.

[51] Haverkamp A, Yon F, Keesey IW, Missbach C, Koenig C, Hansson BS, Baldwin IT, Knaden M, Kessler D. Hawkmoths evaluate scenting flowers with the tip of their proboscis. eLife. 2016;5:1–12. 10.7554/eLife.15039 PMC4884077 27146894

[52] Haverkamp A, Bing J, Badeke E, Hansson BS, Knaden M (2016). Innate olfactory preferences for flowers matching proboscis length ensure optimal energy gain in a hawkmoth. Nat Commun.

[53] Matthews BJ, McBride CS, DeGennaro M, Despo O, Vosshall LB (2016). The neurotranscriptome of the *Aedes aegypti* mosquito. BMC Genomics.

[54] Xu W, Papanicolaou A, Zhang HJ, Anderson A (2016). Expansion of a bitter taste receptor family in a polyphagous insect herbivore. Sci Rep.

[55] Wanner KW, Robertson HM (2008). The gustatory receptor family in the silkworm moth *Bombyx mori* is characterized by a large expansion of a single lineage of putative bitter receptors. Insect Mol Biol.

[56] Thom C, Guerenstein PG, Mechaber WL, Hildebrand JG (2004). Floral CO2 reveals flower profitability to moths. J Chem Ecol.

[57] Bogner F, Boppre M, Ernst KD, Boeckh J (1986). CO2 sensitive receptors on labial palps of Rhodogastria moths (Lepidoptera: Arctiidae): physiology, fine structure and central projection. J Comp Physiol A.

[58] Du LX, Liu Y, Zhang J, Gao XW, Wang B, Wang GR (2018). Identification and characterization of chemosensory genes in the antennal transcriptome of Spodoptera exigua. Comp Biochem Phys D.

[59] Nishino H, Nishikawa M, Yokohari F, Mizunami M (2005). Dual, multilayered somatosensory maps formed by antennal tactile and contact chemosensory afferents in an insect brain. J Comp Neurol.

[60] Popescu A, Couton L, Almaas TJ, Rospars JP, Wright GA, Marion-Poll F, Anton S (2013). Function and central projections of gustatory receptor neurons on the antenna of the noctuid moth *Spodoptera littoralis*. J Comp Physiol A Neuroethol Sens Neural Behav Physiol.

[61] Sato K, Tanaka K, Touhara K (2011). Sugar-regulated cation channel formed by an insect gustatory receptor. Proc Natl Acad Sci U S A.

[62] Seada MA, Ignell R, Anderson P (2016). Morphology and distribution of ovipositor sensilla of female cotton leaf worm Spodoptera littoralis (Lepidoptera: Noctuidae), and evidence for gustatory function. Entomol Sci.

[63] Abuin L, Bargeton B, Ulbrich MH, Isacoff EY, Kellenberger S, Benton R (2011). Functional architecture of olfactory ionotropic glutamate receptors. Neuron.

[64] Croset V, Rytz R, Cummins SF, Budd A, Brawand D, Kaessmann H, Gibson TJ, Benton R (2010). Ancient protostome origin of chemosensory ionotropic glutamate receptors and the evolution of insect taste and olfaction. PLoS Genet.

[65] Gong DP, Zhang HJ, Zhao P, Xia QY, Xiang ZH (2009). The odorant binding protein gene family from the genome of silkworm, *Bombyx mori*. BMC Genomics.

[66] Gong DP, Zhang HJ, Zhao P, Lin Y, Xia QY, Xiang ZH (2007). Identification and expression pattern of the chemosensory protein gene family in the silkworm, *Bombyx mori*. Insect Biochem Mol Biol.

[67] Zhang S, Zhang Z, Wang H, Kong X (2014). Antennal transcriptome analysis and comparison of olfactory genes in two sympatric defoliators, *Dendrolimus houi* and *Dendrolimus kikuchii* (Lepidoptera: Lasiocampidae). Insect Biochem Mol Biol.

[68] Iovinella I, Bozza F, Caputo B, Della Torre A, Pelosi P (2013). Ligand-binding study of *Anopheles gambiae* chemosensory proteins. Chem Senses.

[69] Pitts RJ, Rinker DC, Jones PL, Rokas A, Zwiebel LJ (2011). Transcriptome profiling of chemosensory appendages in the malaria vector *Anopheles gambiae* reveals tissue- and sex-specific signatures of odor coding. BMC Genomics.

[70] Sun M, Liu Y, Walker WB, Liu C, Lin K, Gu S, Zhang Y, Zhou J, Wang G (2013). Identification and characterization of pheromone receptors and interplay between receptors and pheromone binding proteins in the diamondback moth, *Plutella xyllostella*. PloS One.

[71] Grosse-Wilde E, Svatos A, Krieger J (2006). A pheromone-binding protein mediates the bombykol-induced activation of a pheromone receptor in vitro. Chem Senses.

[72] Poivet E, Rharrabe K, Monsempes C, Glaser N, Rochat D, Renou M, Marion-Poll F, Jacquin-Joly E (2012). The use of the sex pheromone as an evolutionary solution to food source selection in caterpillars. Nat Commun.

[73] Lautenschlager C, Leal WS, Clardy J (2007). *Bombyx mori* pheromone-binding protein binding nonpheromone ligands: implications for pheromone recognition. Structure.

[74] Celorio-Mancera Mde L, Sundmalm SM, Vogel H, Rutishauser D, Ytterberg AJ, Zubarev RA, Janz N (2012). Chemosensory proteins, major salivary factors in caterpillar mandibular glands. Insect Biochem Mol Biol.

[75] Durand N, Carot-Sans G, Bozzolan F, Rosell G, Siaussat D, Debernard S, Chertemps T, Maibeche-Coisne M (2011). Degradation of pheromone and plant volatile components by a same odorant-degrading enzyme in the cotton leafworm, *Spodoptera littoralis*. PloS One.

[76] Durand N, Carot-Sans G, Chertemps T, Bozzolan F, Party V, Renou M, Debernard S, Rosell G, Maibeche-Coisne M (2010). Characterization of an antennal carboxylesterase from the pest moth Spodoptera littoralis degrading a host plant odorant. PloS One.

[77] Cock PJA, Fields CJ, Goto N, Heuer ML, Rice PM (2010). The Sanger FASTQ file format for sequences with quality scores, and the Solexa/Illumina FASTQ variants. Nucleic Acids Res.

[78] Ewing B, Hillier L, Wendl MC, Green P (1998). Base-calling of automated sequencer traces using phred. I. Accuracy assessment. Genome Res.

[79] Ewing B, Green P (1998). Base-calling of automated sequencer traces using phred. II. Error probabilities. Genome Res.

[80] Grabherr MG, Haas BJ, Yassour M, Levin JZ, Thompson DA, Amit I, Adiconis X, Fan L, Raychowdhury R, Zeng Q (2011). Full-length transcriptome assembly from RNA-Seq data without a reference genome. Nat Biotechnol.

[81] Li W, Godzik A (2006). Cd-hit: a fast program for clustering and comparing large sets of protein or nucleotide sequences. Bioinformatics.

[82] Simao FA, Waterhouse RM, Ioannidis P, Kriventseva EV, Zdobnov EM (2015). BUSCO: assessing genome assembly and annotation completeness with single-copy orthologs. Bioinformatics.

[83] Waterhouse Robert M, Seppey Mathieu, Simão Felipe A, Manni Mosè, Ioannidis Panagiotis, Klioutchnikov Guennadi, Kriventseva Evgenia V, Zdobnov Evgeny M (2017). BUSCO Applications from Quality Assessments to Gene Prediction and Phylogenomics. Molecular Biology and Evolution.

[84] Camacho C, Coulouris G, Avagyan V, Ma N, Papadopoulos J, Bealer K, Madden TL (2009). BLAST+: architecture and applications. BMC Bioinformatics.

[85] Artimo P, Jonnalagedda M, Arnold K, Baratin D, Csardi G, de Castro E, Duvaud S, Flegel V, Fortier A, Gasteiger E (2012). ExPASy: SIB bioinformatics resource portal. Nucleic Acids Res.

[86] Sievers F, Wilm A, Dineen D, Gibson TJ, Karplus K, Li W, Lopez R, McWilliam H, Remmert M, Soding J (2011). Fast, scalable generation of high-quality protein multiple sequence alignments using Clustal Omega. Mol Syst Biol.

[87] Haas BJ, Papanicolaou A, Yassour M, Grabherr M, Blood PD, Bowden J, Couger MB, Eccles D, Li B, Lieber M (2013). De novo transcript sequence reconstruction from RNA-seq using the Trinity platform for reference generation and analysis. Nat Protoc.

[88] Li B, Dewey CN (2011). RSEM: accurate transcript quantification from RNA-Seq data with or without a reference genome. BMC Bioinformatics.

[89] Langmead B, Trapnell C, Pop M, Salzberg SL (2009). Ultrafast and memory-efficient alignment of short DNA sequences to the human genome. Genome Biol.

[90] Li H, Handsaker B, Wysoker A, Fennell T, Ruan J, Homer N, Marth G, Abecasis G, Durbin R (2009). Genome Project Data Processing S: The Sequence Alignment/Map format and SAMtools. Bioinformatics.

[91] Bengtsson JM, Trona F, Montagné N, Anfora G, Ignell R, Witzgall P, Jacquin-Joly E (2012). Putative chemosensory receptors of the codling moth, *Cydia pomonella*, identified by antennal transcriptome analysis. PloS One.

[92] Yu QY, Lu C, Li WL, Xiang ZH, Zhang Z (2009). Annotation and expression of carboxylesterases in the silkworm, *Bombyx mori*. BMC Genomics.

[93] Ai J, Zhu Y, Duan J, Yu Q, Zhang G, Wan F, Xiang ZH (2011). Genome-wide analysis of cytochrome P450 monooxygenase genes in the silkworm, *Bombyx mori*. Gene.

[94] Tijet N, Helvig C, Feyereisen R (2001). The cytochrome P450 gene superfamily in *Drosophila melanogaster*: annotation, intron-exon organization and phylogeny. Gene.

[95] Katoh K, Toh H (2010). Parallelization of the MAFFT multiple sequence alignment program. Bioinformatics.

[96] Tamura K, Stecher G, Peterson D, Filipski A, Kumar S (2013). MEGA6: Molecular Evolutionary Genetics Analysis version 6.0. Mol Biol Evol.

