# Supplementary material for: Transcriptome Analysis of Gene Families Involved in Chemosensory Function in Spodoptera littoralis (Lepidoptera: Noctuidae)
Source: BMC Genomics. 2019 May 28;20:428. doi: 10.1186/s12864-019-5815-x (PMC6540431; doi:10.1186/s12864-019-5815-x)
Supplement: Supplementary file 3 — Novel Candidate Chemosensory Gene Informatics. Includes annotation name, ORF size, best blast hit and other parameters. (DOCX 178 kb) [file 12864_2019_5815_MOESM3_ESM.docx]

**Novel Candidate Chemosensory Gene Informatics**

| **Gene Name** | **ORF Status** | **ORF Size** | **Best Blastp Hit in NCBI nr database** | **E-value** | **Identity** |
| --- | --- | --- | --- | --- | --- |
| SlitOR47 | Complete | 398 | *Athetis lepigone* OR44 | 0 | 92% |
| SlitOR48 | Complete | 411 | *Athetis dissimilis* OR17 | 0 | 73% |
| SlitOR49 | Complete | 390 | *Helicoverpa armigera* OR57 | 0 | 67% |
| SlitOR50 | Complete | 396 | *A. dissimilis* OR47 | 2.00E-170 | 58% |
| SlitOR51 | Complete | 402 | *H. armigera* OR41 | 0 | 87% |
| SlitOR52 | Complete | 387 | *A. lepigone* OR16 | 0 | 77% |
| SlitOR53 | Complete | 404 | *A. lepigone* OR61 | 0 | 80% |
| SlitOR54 | Complete | 400 | *Eogystia hippophaecolus* OR3 | 4.00E-142 | 50% |
| SlitOR55 | Complete | 414 | *Operophtera brumata* OR50 | 0 | 65% |
| SlitOR56 | Complete | 432 | *Spodoptera exigua* OR6 | 0 | 75% |
| SlitOR57 | Complete | 397 | *H. armigera* OR8 | 0 | 63% |
| SlitOR58 | Complete | 422 | *A. lepigone* OR20 | 0 | 71% |
| SlitOR59 | Complete | 395 | *H. armigera* OR43 | 0 | 72% |
| SlitOR60 | Incomplete | 346 | *E. hippophaecolus* OR40 | 9.00E-78 | 39% |
| SlitGR1¶ | Incomplete | 406 | *H. armigera* GR1 | 0 | 87% |
| SlitGR6¶ | Incomplete | 471 | *H. armigera* GR1 | 0 | 90% |
| SlitGR9¶ | Complete | 465 | *H. armigera* GR9 | 0 | 97% |
| SlitGR11¶ | Incomplete | 328 | *B. mori* GR68 | 3.00E-83 | 43% |
| SlitGR13¶ | Complete | 431 | *H. armigera* GR5 | 0 | 76% |
| SlitGR14¶ | Complete | 476 | *H. armigera* GR5 | 0 | 78% |
| SlitGR69 | Incomplete | 276 | *B. mori* GR67 | 3.00E-93 | 75% |
| SlitGR70¶ | Complete | 413 | *A. dissimilis* GR10 | 6.00E-95 | 49% |
| SlitGR73¶ | Incomplete | 261 | *Bombyx mori* GR53 | 2.00E-78 | 50% |
| SlitGR158¶ | Incomplete | 278 | *B. mori* GR60 | 4.00E-21 | 30% |
| SlitGR206¶ | Incomplete | 252 | *B. mori* GR60 | 3.00E-21 | 34% |
| SlitGR230 | Complete | 276 | *Helicoverpa assulta* GR17 | 2.00E-25 | 38% |
| SlitIR7d | Complete | 597 | *A. lepigone* IR7d.2 | 0 | 68% |
| SlitIR60a | Complete | 661 | *O. brumata* IR | 0 | 54% |
| SlitPBP4 | Complete | 213 | *Orthaga achatina* PBP1 | 5.00E-20 | 33% |
| SlitOBP8 | Complete | 146 | *Spodoptera litura* OBP25 | 2.00E-35 | 46% |
| SlitOBP32 | Complete | 176 | *S. litura* OBP11 | 1.00E-91 | 91% |
| SlitOBP33 | Complete | 153 | *S. litura* OBP25 | 9.00E-86 | 85% |
| SlitOBP34 | Complete | 160 | *S. litura* OBP6 | 3.00E-98 | 89% |
| SlitOBP35 | Complete | 156 | *S. litura* OBP33 | 1.00E-110 | 100% |
| SlitOBP36 | Complete | 147 | *S. litura* OBP27 | 1.00E-105 | 99% |
| SlitOBP37 | Complete | 182 | *Danaus plexippus* OBP2 | 1.00E-106 | 85% |
| SlitOBP38 | Complete | 140 | *S. exigua* OBP2 | 1.00E-09 | 30% |
| SlitOBP39 | Incomplete | 210 | *H. assulta* OBP19 | 6.00E-49 | 48% |
| SlitOBP40 | Complete | 209 | *S. litura* OBP19 | 2.00E-148 | 98% |
| SlitOBP41 | Complete | 212 | *B. mori* OBP2 | 1.00E-107 | 69% |
| SlitOBP42 | Complete | 146 | *Chilo suppressalis* OBP5 | 3.00E-24 | 40% |
| SlitOBP43 | Incomplete | 133 | *H. assulta* OBP9 | 2.00E-29 | 41% |
| SlitOBP44 | Complete | 150 | *S. litura* OBP22 | 1.00E-101 | 100% |
| SlitOBP45 | Incomplete | 138 | *Sesamia inferens* OBP6 | 4.00E-60 | 74% |
| SlitCSP22 | Complete | 107 | *S. exigua* CSP8 | 3.00E-71 | 97% |
| SlitCXE8b | Complete | 546 | *S. inferens* CXE18 | 0 | 70% |
| SlitCCE001k | Complete | 549 | *S. exigua* CXE31 | 0 | 83% |
| SlitCCE016e | Incomplete | 435 | *Papilio polytes* CXEJHX2 | 5.00E-111 | 41% |
| SlitCCE003a | Complete | 669 | *O. brumata* alphaEsterase3 | 0 | 45% |
| SlitCCE014a | Complete | 749 | *H. armigera* CCE014a | 0 | 77% |
| SlitCCE001g | Complete | 558 | *B. mori* CXEv6l | 0 | 66% |
| SlitCCE019a | Complete | 531 | *Papolio machaon* EstFE4 | 0 | 55% |
| SlitCCE006j | Complete | 532 | *B. mori* JH-Esterase | 1.00E-160 | 47% |
| SlitCCE018c | Complete | 535 | *Bombyx mandarina* carboxylesterase | 0 | 54% |
| SlitCCE001j | Complete | 554 | *S. inferens* CXE14 | 0 | 68% |
| SlitCCE006g | Complete | 522 | *B. mori* Ch.Esterase2-X1 | 2.00E-113 | 42% |
| SlitCCE006a | Incomplete | 394 | *H. armigera* CCE006a | 0 | 67% |
| SlitCCE025a | Complete | 564 | *H. armigera* CCE025a | 0 | 79% |
| SlitCCE006n | Complete | 587 | *H. armigera* CCE006b | 9.00E-171 | 49% |
| SlitCCE021a | Complete | 583 | *H. armigera* CXE-like | 0 | 74% |
| SlitCCE016f | Complete | 560 | *Agrotis ipsilon* carboxylesterase | 0 | 54% |
| SlitCCE002a | Complete | 555 | *H. armigera* CCE002a | 0 | 69% |
| SlitCCE001q | Complete | 565 | *Papilio xuthus* Esterase FE4 | 1.00E-141 | 43% |
| SlitCCE016h | Incomplete | 541 | *S. inferens* CXE10 | 0 | 56% |
| SlitCCE022a | Complete | 571 | *O. brumata* CXE-like | 0 | 55% |
| SlitCCE012a | Incomplete | 540 | *O. brumata* alpha-esterase 40 | 0 | 53% |
| SlitCCE006k | Complete | 549 | *H. armigera* CCE006b | 0 | 59% |
| SlitCCE023a | Incomplete | 523 | *Amyelois transitella* JH-esterase-like | 0 | 66% |
| SlitCCE006m | Incomplete | 498 | *H. armigera* CCE006b | 0 | 54% |
| SlitCCE006c | Incomplete | 350 | *H. armigera* CCE006c | 1.00E-106 | 50% |
| SlitCCE001l | Incomplete | 304 | *H. armigera* CCE001c | 1.00E-116 | 62% |
| SlitCYP15C1 | Complete | 491 | *B. mori* CYP15C1 | 0 | 69% |
| SlitCYP301B1 | Complete | 544 | *P. xuthus* CYP49A1 | 0 | 83% |
| SlitCYP303A1 | Complete | 501 | *H. armigera* CYP303A1 | 0 | 92% |
| SlitCYP3097A1 | Complete | 501 | *A. transitella* CYP6B7-like | 7.00E-115 | 39% |
| SlitCYP321A15 | Complete | 499 | *Spodoptera frugiperda* CYP321A7 | 0 | 66% |
| SlitCYP321A8 | Incomplete | 345 | *S. frugiperda* CYP321A8 | 0 | 90% |
| SlitCYP321B1 | Complete | 495 | *S. litura* CYP321B1 | 0 | 99% |
| SlitCYP321B4 | Complete | 496 | *H. armigera* CYP321B1 | 0 | 69% |
| SlitCYP332A1 | Complete | 503 | *H. armigera* CYP332A1 | 0 | 77% |
| SlitCYP333B4 | Complete | 509 | *H. armigera* CYP333B3 | 0 | 76% |
| SlitCYP337B5 | Complete | 492 | *S. frugiperda* CYP337B5 | 0 | 93% |
| SlitCYP340AD4 | Incomplete | 451 | *H. armigera* CYP340G1 | 6.00E-170 | 52% |
| SlitCYP340AH2 | Complete | 493 | *Spodoptera littoralis* CYP340AB1 | 0 | 53% |
| SlitCYP340AQ1 | Complete | 485 | *S. littoralis* CYP340AB1 | 4.00E-155 | 45% |
| SlitCYP340G2 | Complete | 492 | *H. armigera* CYP340G1 | 0 | 68% |
| SlitCYP340L2 | Incomplete | 470 | *S. frugiperda* CYP340L1 | 0 | 55% |
| SlitCYP341B17 | Complete | 501 | *B.mori* CYP341B1 | 0 | 54% |
| SlitCYP341B19 | Incomplete | 508 | *H. armigera* CYP341D1 | 0 | 65% |
| SlitCYP49A1 | Complete | 531 | *P. xuthus* CYP49A1 | 0 | 72% |
| SlitCYP4AU15 | Complete | 485 | *H. armigera* CYP4AU1 | 0 | 80% |
| SlitCYP4AU18 | Complete | 494 | *B. mori* CYP4C3 | 0 | 78% |
| SlitCYP4CG16 | Incomplete | 423 | *P. polytes* CYP4C1-like | 5.00E-170 | 56% |
| SlitCYP4CG18 | Complete | 507 | *Manduca sexta* CYP4CG1 | 0 | 55% |
| SlitCYP4G109 | Complete | 566 | *H. armigera* CYP4G9 | 0 | 86% |
| SlitCYP4G74 | Complete | 562 | *S. frugiperda* CYP4G74 | 0 | 94% |
| SlitCYP4M14 | Complete | 503 | *S. litura* CYP4M14v1 | 0 | 97% |
| SlitCYP4M18 | Complete | 499 | *Helicoverpa zea* CYP4M6 | 0 | 76% |
| SlitCYP4S9 | Complete | 490 | *S. litura* CYP4S9v1 | 0 | 98% |
| SlitCYP6AB60 | Incomplete | 513 | *O. brumata* CYP | 0 | 72% |
| SlitCYP6AB61 | Incomplete | 285 | *A. ipsilon* CYP6AB | 0 | 89% |
| SlitCYP6AE70 | Complete | 519 | *S. littoralis* CYP6AE47 | 0 | 73% |
| SlitCYP6AE77 | Incomplete | 296 | *S. littoralis* CYP6AE47 | 0 | 67% |
| SlitCYP6AW1 | Complete | 495 | *P. machaon* CYP6J1-like | 0 | 76% |
| SlitCYP6B58 | Incomplete | 514 | *S. litura* CYP6B58 | 0 | 98% |
| SlitCYP9A27 | Incomplete | 529 | *S. frugiperda* CYP9A58 | 0 | 66% |
| SlitCYP9A39 | Incomplete | 590 | *S. litura* CYP9A39 | 0 | 91% |
| SlitCYP9A40 | Complete | 529 | *S. litura* CYP9A40 | 0 | 97% |
| SlitCYP9A59 | Incomplete | 545 | *S. frugiperda* CYP9A59 | 0 | 81% |
| SlitCYP9A60 | Incomplete | 317 | *S. frugiperda* CYP9A60 | 0 | 95% |
| SlitCYP9A81 | Complete | 530 | *S. exigua* CYP9A11 | 0 | 87% |
| SlitCYP9AJ1 | Incomplete | 367 | *H. armigera* CYP9AJ3 | 0 | 85% |
| SlitCYP9BS1 | Incomplete | 494 | *P. polytes* probable CYP9F2 | 0 | 48% |
| SlitCYP9G17 | Complete | 492 | *H. armigera* | 0 | 73% |
|  |  |  |  |  |  |

¶ indicates presence of conserved insect GR C terminus motif MYhhhhhQF
