# Supplementary material for: Transcriptome Analysis of Gene Families Involved in Chemosensory Function in Spodoptera littoralis (Lepidoptera: Noctuidae)
Source: BMC Genomics. 2019 May 28;20:428. doi: 10.1186/s12864-019-5815-x (PMC6540431; doi:10.1186/s12864-019-5815-x)
Supplement: Supplementary file 4 — Estimated Transcript Abundance Peak Values by Gene Family, Tissue and Sex, Fragments per Kilobase per Million (FPKM). (DOCX 15 kb) [file 12864_2019_5815_MOESM4_ESM.docx]

**Estimated Transcript Abundance Peak Values by Gene Family, Tissue and Sex, Fragments per Kilobase per Million reads (FPKM).**

|  | Antennae | | Brain | | Body | | Proboscis | |
| --- | --- | --- | --- | --- | --- | --- | --- | --- |
|  | Male | Female | Male | Female | Male | Female | Male | Female |
| OR | 955.3 | 348.2 | 6.40 | 3.75 | 1.98 | 1.92 | 1.87 | 2.22 |
| GR | 8.70 | 10.97 | 9.35 | 7.96 | 11.58 | 1.72 | 24.01 | 61.54 |
| IR | 166.6 | 136.9 | 3.86 | 3.44 | 6.38 | 1.62 | 22.06 | 22.73 |
| OBP | 7.20x10^4^ | 2.00x10^4^ | 1.71x10^3^ | 6.10x10^3^ | 588.9 | 71.1 | 3.34x10^3^ | 2.11x10^3^ |
| CSP | 6.27x10^3^ | 6.26x10^3^ | 977.0 | 2.25x10^3^ | 5.60x10^3^ | 876.1 | 3.40x10^4^ | 5.42x10^4^ |
| CXE/CCE | 735.9 | 451.1 | 52.9 | 132.1 | 574.1 | 374.3 | 326.0 | 1.11x10^3^ |
| CYP | 1.88x10^3^ | 9.01x10^3^ | 913.8 | 234.4 | 852.7 | 283.2 | 2.44x10^3^ | 3.13x10^3^ |
