# Supplementary figures and images for: Transcriptome Analysis of Gene Families Involved in Chemosensory Function in Spodoptera littoralis (Lepidoptera: Noctuidae)
Source: BMC Genomics. 2019 May 28;20:428. doi: 10.1186/s12864-019-5815-x (PMC6540431; doi:10.1186/s12864-019-5815-x)

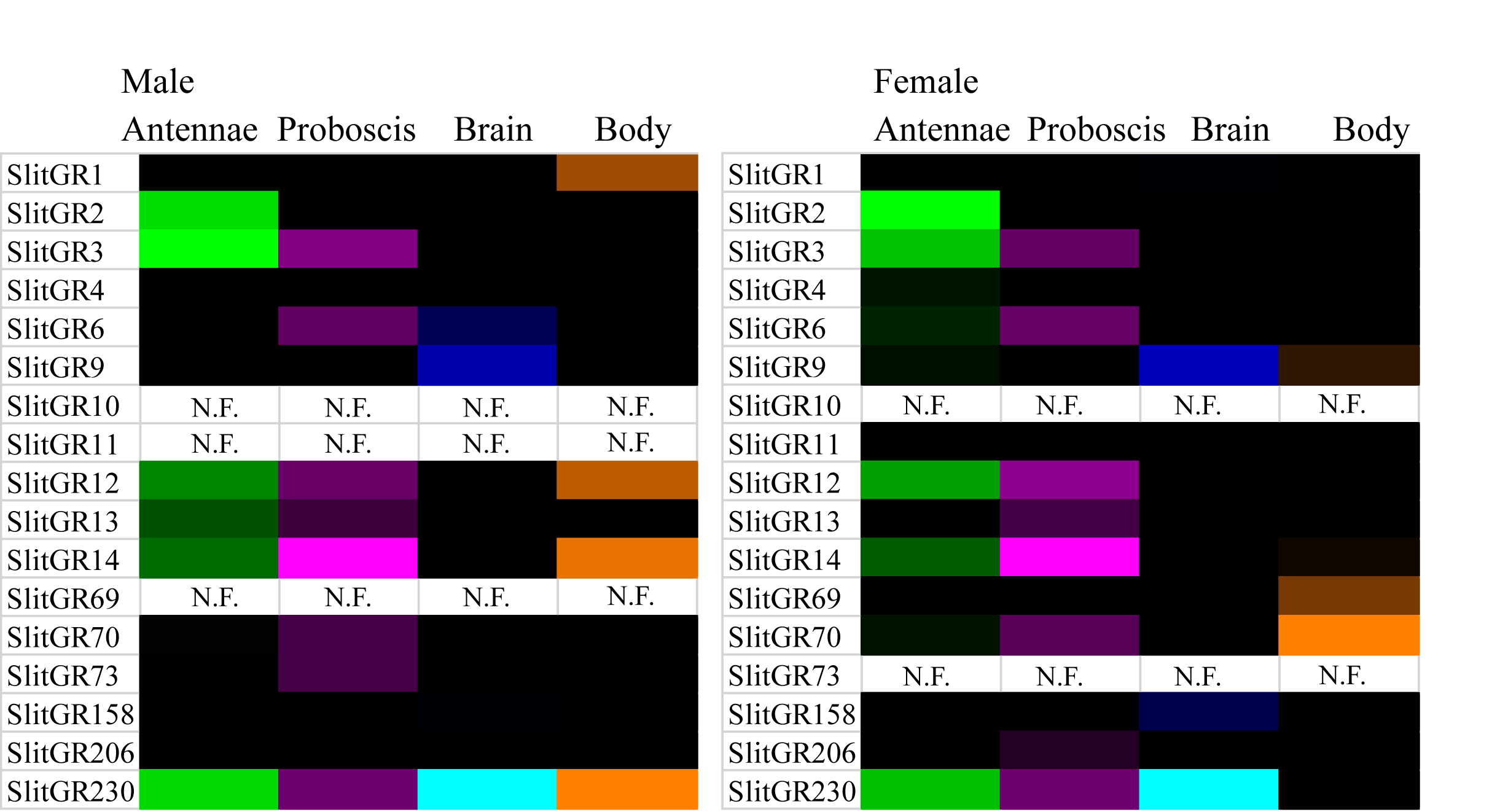

Supplement: Supplementary file 8 — Heat-plot of relative expression values for S. littoralis GRs. Estimation of abundance values determined by read mapping. Black indicates low/no expression, dark colors indicate low/moderate expression, bright colors indicate moderate/high expression. Color plots represent binary log of FPKM plus one for each gene (See Additional file 6 for raw data). Color scales for each tissue type are independent of other tissue types. Range of values for Male Antenna: 0 – 3.28; Male Brain: 0 – 3.37; Male Body: 0 – 3.65; Male Proboscis: 0 – 4.64; Female Antennae: 0 – 3.58; Female Brain: 0 – 3.16; Female Body: 0 – 1.44; Female Proboscis: 0 – 5.97. (TIF 599 kb) [file 12864_2019_5815_MOESM8_ESM.tif]

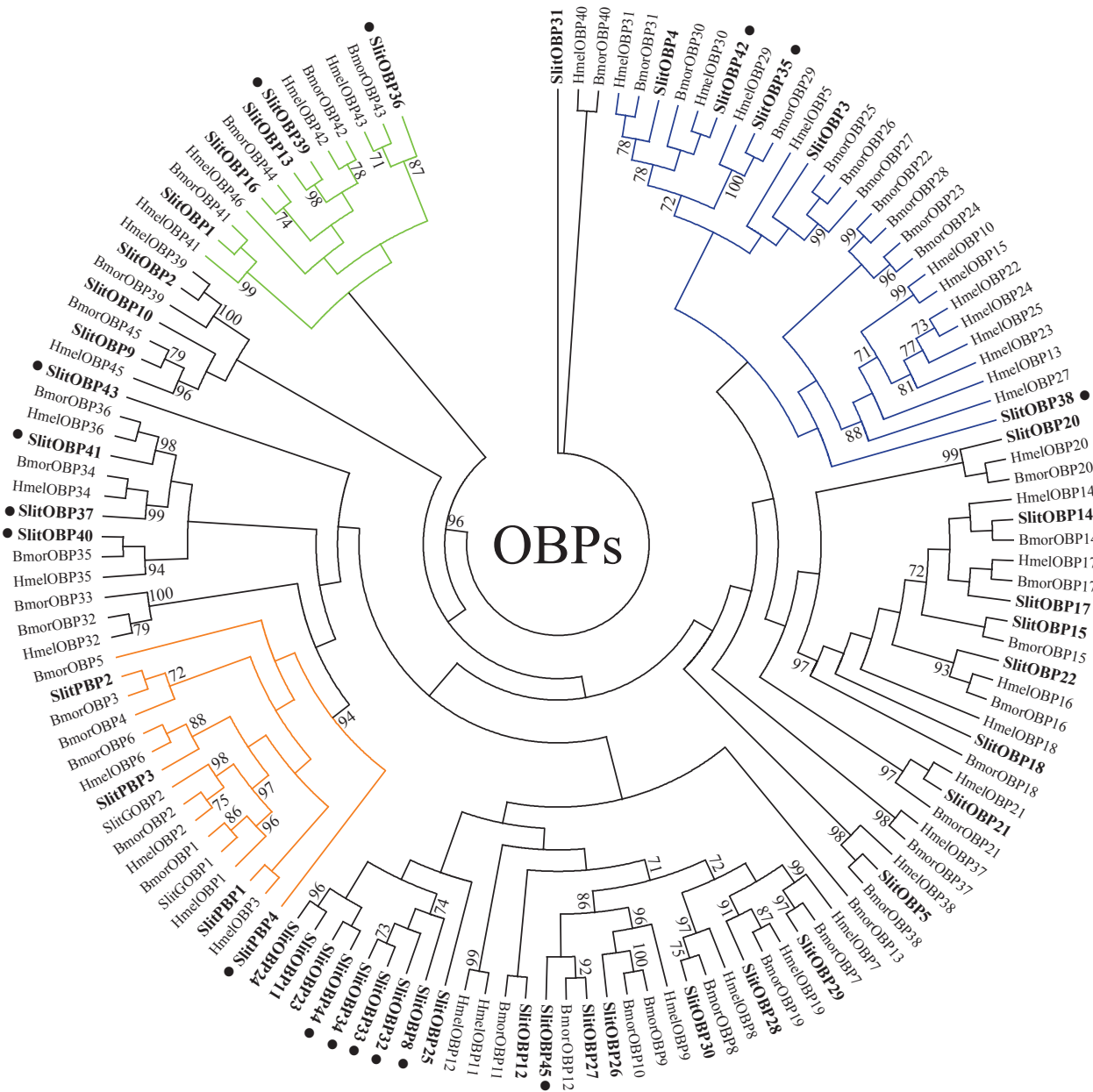

Supplement: Supplementary file 11 — Maximum likelihood cladogram of candidate SlitOBP sequences with other lepidopteran OBP sequences. Unrooted. Includes sequences from S. littoralis (Slit), Heliconius melpomene (Hmel) and Bombyx mori (Bmor). Branches containing “Plus-C” subfamily OBPs are colored green; branches containing “Minus-C” subfamily OBPs are colored blue; branches containing putative pheromone binding proteins (PBPs) and general odorant binding proteins (GOBPs) are colored orange; S. littoralis OBPs are indicated with a larger bold font, and novel S. littoralis OBPs are marked with a “•”. Node support was assessed with 600 bootstrap replicates and values greater than 70% are shown. (PDF 368 kb) [file 12864_2019_5815_MOESM11_ESM.pdf]

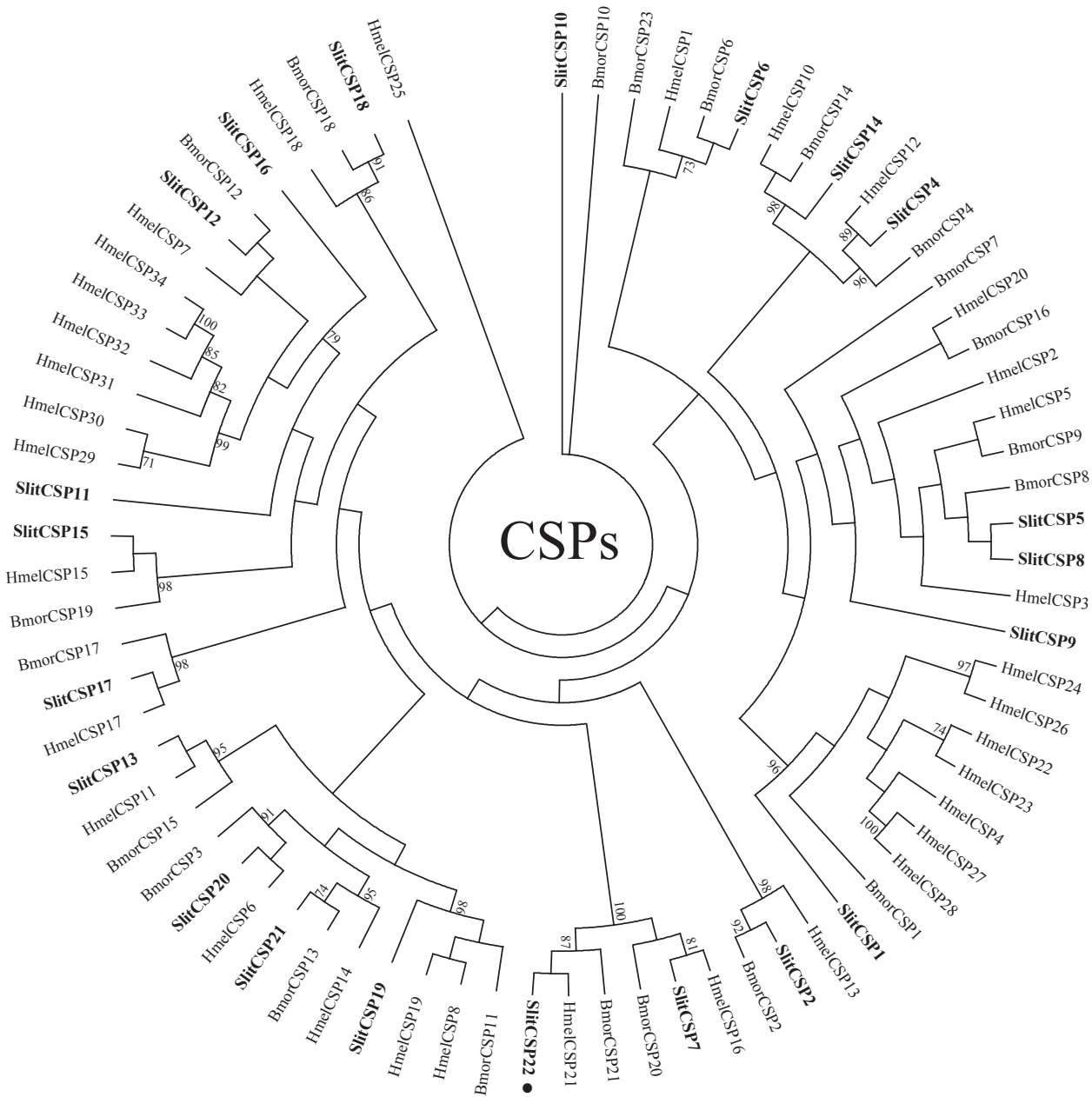

Supplement: Supplementary file 13 — Maximum likelihood cladogram of candidate SlitCSP sequences with other lepidopteran CSP sequences. Unrooted. Includes sequences from S. littoralis (Slit), Heliconius melpomene (Hmel) and Bombyx mori (Bmor). S. littoralis CSPs are indicated with a larger bold font, and novel S. littoralis CSP is marked with a “•”. Node support was assessed with 600 bootstrap replicates and values greater than 70% are shown. (PDF 283 kb) [file 12864_2019_5815_MOESM13_ESM.pdf]

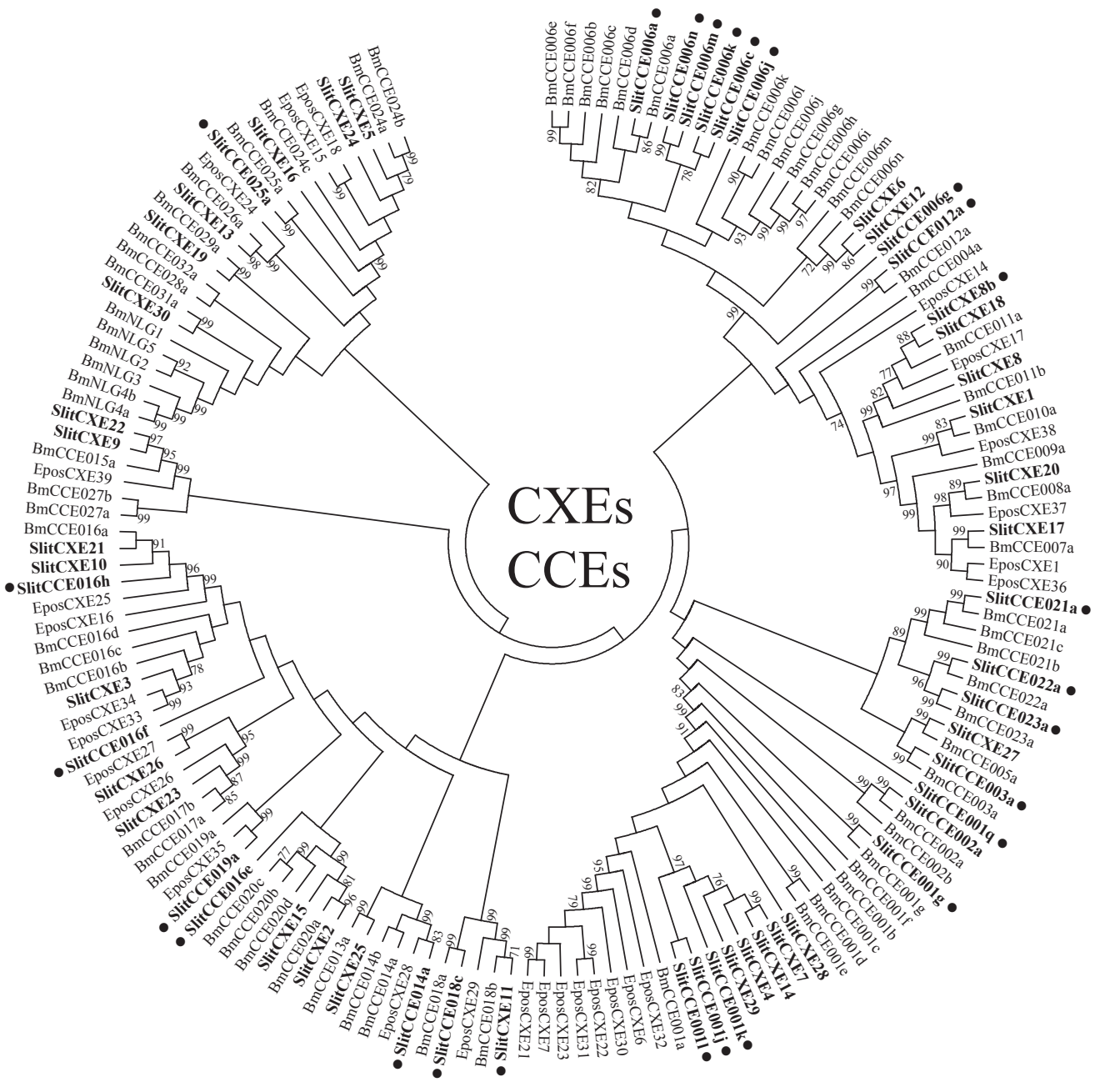

Supplement: Supplementary file 15 — Maximum likelihood cladogram of candidate SlitCXE sequences with other lepidopteran CXE/CCE sequences. Unrooted. Includes sequences from S. littoralis (Sl), E. postvittana (Epos) and Bombyx mori (Bm). S. littoralis CXE/CCEs are indicated with a larger bold font, and novel S. littoralis CXE/CCEs is marked with a “•”. Node support was assessed with 600 bootstrap replicates and values greater than 70% are shown. (PDF 386 kb) [file 12864_2019_5815_MOESM15_ESM.pdf]

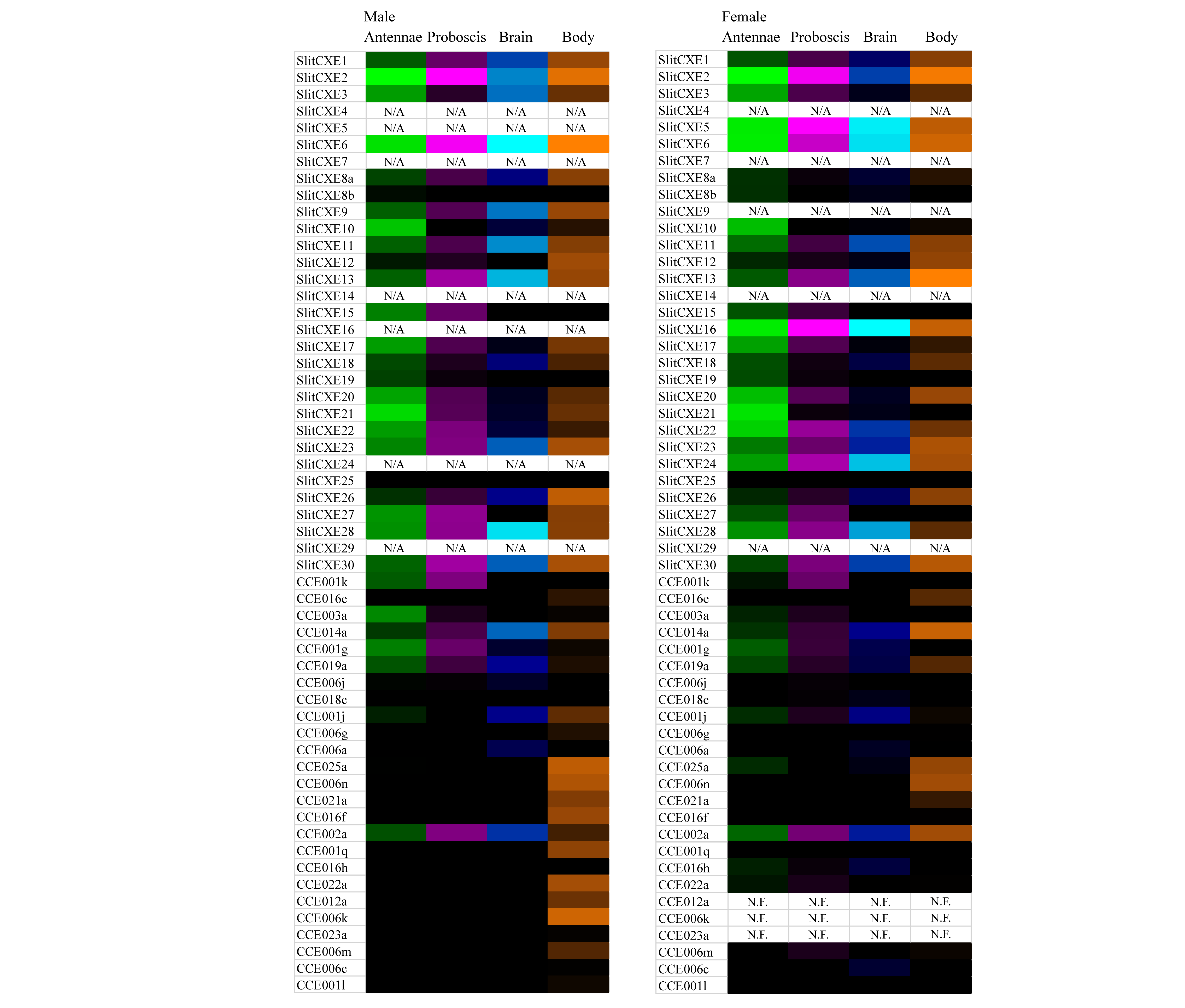

Supplement: Supplementary file 16 — Heat-plot of relative expression values for S. littoralis CXE/CCEs. Estimation of abundance values determined by read mapping. Black indicates low/no expression, dark colors indicate low/moderate expression, bright colors indicate moderate/high expression. Color plots represent binary log of FPKM plus one for each gene (See Additional file 6 for raw data). Color scales for each tissue type are independent of other tissue types. “N.F.” indicates that gene transcripts were not found in respective transcriptome. “N/A” indicates that unique gene model could not be resolved for gene transcripts in respective transcriptome due to co-assembly of highly similar gene models. Range of values for Male Antenna: 0 – 9.53; Male Brain: 0 – 5.75; Male Body: 0 – 9.17; Male Proboscis: 0 – 8.71; Female Antennae: 0 – 8.82; Female Brain: 0 – 7.06; Female Body: 0 – 8.55; Female Proboscis: 0 – 10.11. (TIF 2410 kb) [file 12864_2019_5815_MOESM16_ESM.tif]

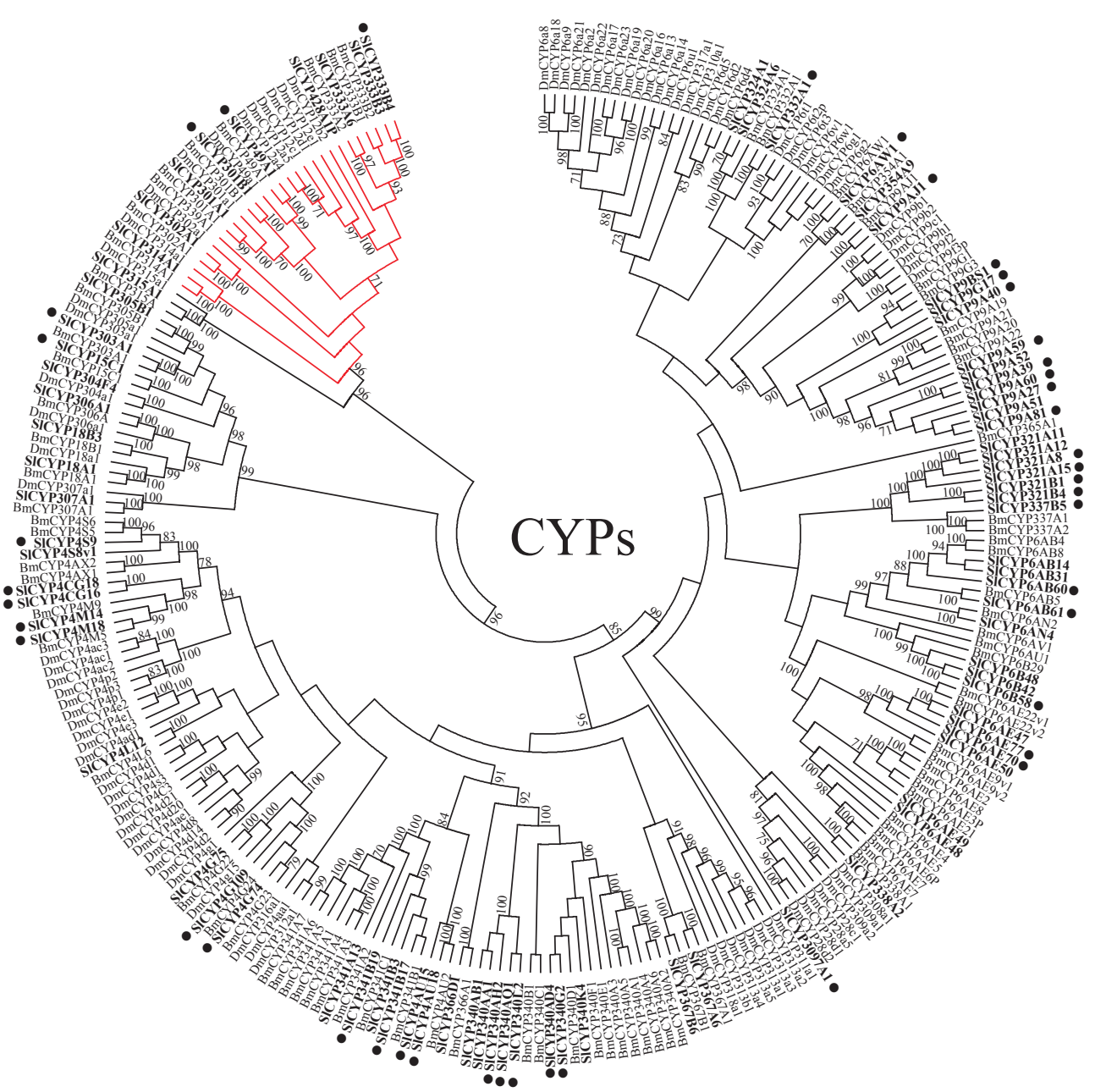

Supplement: Supplementary file 18 — Maximum likelihood phylogenetic tree of candidate SlitCYPs sequences with other insect CYP sequences. Unrooted. Includes sequences from S. littoralis (Sl), D. melanogaster (Dmel) and B. mori (Bmor). Branches containing mitochondrial clan CYPs are colored red. S. littoralis CYPs are indicated with a larger bold font, and novel S. littoralis CYPs are marked with a “•”. Node support was assessed with 600 bootstrap replicates and values greater than 70% are shown. (PDF 532 kb) [file 12864_2019_5815_MOESM18_ESM.pdf]

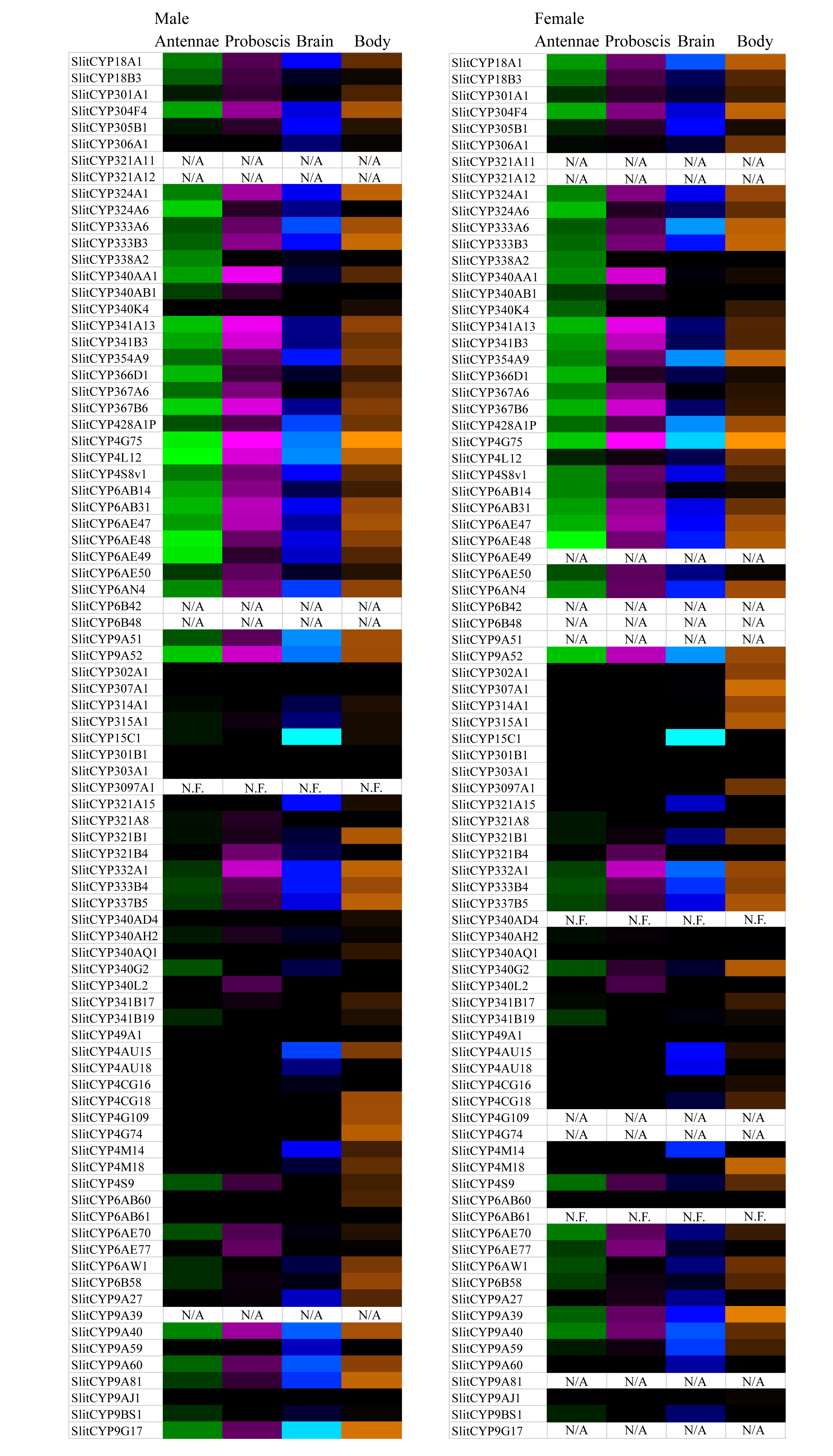

Supplement: Supplementary file 19 — Heat-plot of relative expression values for SlitCYPs. Estimation of abundance values determined by read mapping. Black indicates low/no expression, dark colors indicate low/moderate expression, bright colors indicate moderate/high expression. Color plots represent binary log of FPKM plus one for each gene (See Additional file 6 for raw data). Color scales for each tissue type are independent of other tissue types. “N.F.” indicates that gene transcripts were not found in respective transcriptome. “N/A” indicates that unique gene model could not be resolved for gene transcripts in respective transcriptome due to co-assembly of highly similar gene models. Range of values for Male Antenna: 0 – 11.28; Male Brain: 0 – 9.83; Male Body: 0 – 9.73; Male Proboscis: 0 – 11.61; Female Antennae: 0 – 13.14; Female Brain: 0 – 7.87; Female Body: 0 – 8.15; Female Proboscis: 0 – 12.11. (TIF 3197 kb) [file 12864_2019_5815_MOESM19_ESM.tif]
