# Supplementary material for: Transcriptome Analysis of Gene Families Involved in Chemosensory Function in Spodoptera littoralis (Lepidoptera: Noctuidae)
Source: BMC Genomics. 2019 May 28;20:428. doi: 10.1186/s12864-019-5815-x (PMC6540431; doi:10.1186/s12864-019-5815-x)
Supplement: Supplementary file 21 — supplemental materials and methods. (DOCX 22 kb) [file 12864_2019_5815_MOESM21_ESM.docx]

**Supplemental Materials and Methods**

**Transcriptome Analysis of Gene Families Involved in Chemosensory Function in *Spodoptera littoralis* (Lepidoptera: Noctuidae)**

**Authors –** Walker III, William B.,^1*^, Roy, Amit^1,2^, Anderson, Peter^1^, Schlyter, Fredrik^1,2^, Hansson, Bill S.^3^, Larsson, Mattias C.^1^

**Authors Affiliations –**

1 - Department of Plant Protection Biology, Swedish University of Agricultural Sciences, Sundsvägen 14, 230 53 Alnarp, Sweden

2 - Faculty of Forestry and Wood Sciences, EXTEMIT-K, Czech University of Life Sciences, Kamýcká 1176, Prague 6, 165 21 Suchdol, Czech Republic.

3 - Department of Evolutionary Neuroethology, Max Planck Institute for Chemical Ecology, 07745 Jena, Germany.

* - corresponding author: william.b.walker.iii@slu.se

Swedish University of Agricultural Sciences

Box 102

23053, Alnarp

Sweden

1. **cDNA Library Preparation**

According to BGI protocol, total RNA was converted to a cDNA library for sequencing as follows: first, mRNA was enriched by using the oligo (dT) magnetic beads. Then the mRNA was fragmented into short fragments (200~500 bp) with proprietary fragment buffer treatment. First-strand cDNA was synthesized by random hexamer-primer with the mRNA fragments as templates. Buffer, dNTPs, RNase H and DNA polymerase I were used to synthesize the second strand. The double stranded cDNAs were purified with the QiaQuick PCR extraction kit (Qiagen), are used for end repair and base A addition. Finally, sequencing adaptors were ligated to the fragments. The fragments were purified by Agarose gel electrophoresis and amplified using PCR to produce libraries for RNA sequencing.

1. **Quality Control (QC) of Raw Sequenced Reads**

First, Trimmomatic software (version 0.30) was utilized to trim low quality bases from the 3’ end of each read, with the TRAILING:20 command [1]. Next, an attempt was made to remove contaminant reads from other species as well as genes containing mitochondrial genome sequence. For this, reads in the trimmed files were separately mapped to the human genome (Build Number 37), *E. coli* genome (C strain) or a *Spodoptera exigua* mitochondrial genome [2]*.* Read mapping was carried out with Bowtie software (version 1.0.0)[3]. For each read mapping process, an output file was generated containing all reads that mapped to the source genome with at most three base mismatches. For each sample, the three bowtie output files were concatenated, and an in-house perl script was executed to remove all read pairs for which at least one of the reads mapped to one of the source genomes.

1. **Manual Editing of Chemosensory Gene Sequence Information for Downstream Quantitative Analyses**

Each sequence in the Trinity.fasta file is given a unique sequence identification name, as follows: compX_cY_seqZ, with “comp” level clusters typically consisting of several related sequences. After transcriptome assembly and chemosensory gene annotations, it was apparent that it would be necessary to manually edit transcriptome sequence clusters to facilitate appropriate quantitative analysis of annotated chemosensory genes. In some cases specific gene sequences were scattered across different trinity components (“comp” level sequences) or subcomponents (“c” level sequences). Alternatively, in some cases, sequence subcomponents contained transcripts that encoded proteins unrelated to the chemosensory gene in question. Additionally, in other cases, ORFs of two unrelated genes were merged together into the same component sequence, producing a chimeric fusion transcript [4]. In order for accurate quantitative analysis of gene expression, it is necessary that each subcomponent contain sequence information for one and only one putative gene.

In order to accomplish this, chimeric sequences were first strictly defined as those where multiple ORFs were present in one sequence and there were distinct patterns of read mapping of the input sequence reads to the different ORFs in the assembled sequence transcripts. In such cases, the read mapping patterns were typically identified by the presence of few (e.g. less than 10) reads with minimal overlap (e.g. less than 40 basepairs) connecting two unrelated genes, as identified with the visualization of reads mapped to the transcriptomes, with Integrated Genomics Viewer software [5], see section 8 below) For these chimeric sequences, ORFs were manually split in the Trinity98.fasta files and given arbitrary unique sequence identification tags appropriate to their location within the transcriptome. Additionally, for genes scattered across multiple components or subcomponents, a gene_trans_map file was generated with RSEM software [6] using the “extract-transcript-to-gene-map-from-trinity” perl script, for editing in order to manually define the gene subcomponent models for downstream quantitative analyses. For these, subcomponents were redefined to reflect the splitting of chimeric sequences, as well of the incorporation of all sequences relevant to one gene under the same subcomponent.

**References**

1. Bolger AM, Lohse M, Usadel B. Trimmomatic: a flexible trimmer for Illumina sequence data. Bioinformatics. 2014; 30(15):2114-2120.

2. Wu QL, Gong YJ, Gu Y, Wei SJ. The complete mitochondrial genome of the beet armyworm Spodoptera exigua (Hubner) (Lepodiptera: Noctuidae). Mitochondrial DNA. 2013; 24(1):31-33.

3. Langmead B, Trapnell C, Pop M, Salzberg SL. Ultrafast and memory-efficient alignment of short DNA sequences to the human genome. Genome Biol. 2009; 10(3):R25.

4. Yang Y, Smith SA. Optimizing de novo assembly of short-read RNA-seq data for phylogenomics. BMC Genomics. 2013; 14:328.

5. Thorvaldsdottir H, Robinson JT, Mesirov JP. Integrative Genomics Viewer (IGV): high-performance genomics data visualization and exploration. Brief Bioinform. 2013; 14(2):178-192.

6. Li B, Dewey CN. RSEM: accurate transcript quantification from RNA-Seq data with or without a reference genome. BMC Bioinform. 2011; 12:323.
