# Supplementary material for: Transcriptome Analysis of Gene Families Involved in Chemosensory Function in Spodoptera littoralis (Lepidoptera: Noctuidae)
Source: BMC Genomics. 2019 May 28;20:428. doi: 10.1186/s12864-019-5815-x (PMC6540431; doi:10.1186/s12864-019-5815-x)
Supplement: Supplementary file 23 — uncropped PCR gels, including all experimental assays and no template-controls. For all gels, 1kb Gene Ruler ladder (Thermo Fisher Scientific) was used . MVA – male virgin antennae, FVA – female virgin antennae, MVPR – male virgin proboscis, FVPR, female virgin proboscis, MVBR – male virgin brain, FVBR – female virgin brain. On some gels, part of the gel space was used for experiments unrelated to this manuscript; those sections are indicated as “not applicable.” (PDF 5006 kb) [file 12864_2019_5815_MOESM23_ESM.pdf]

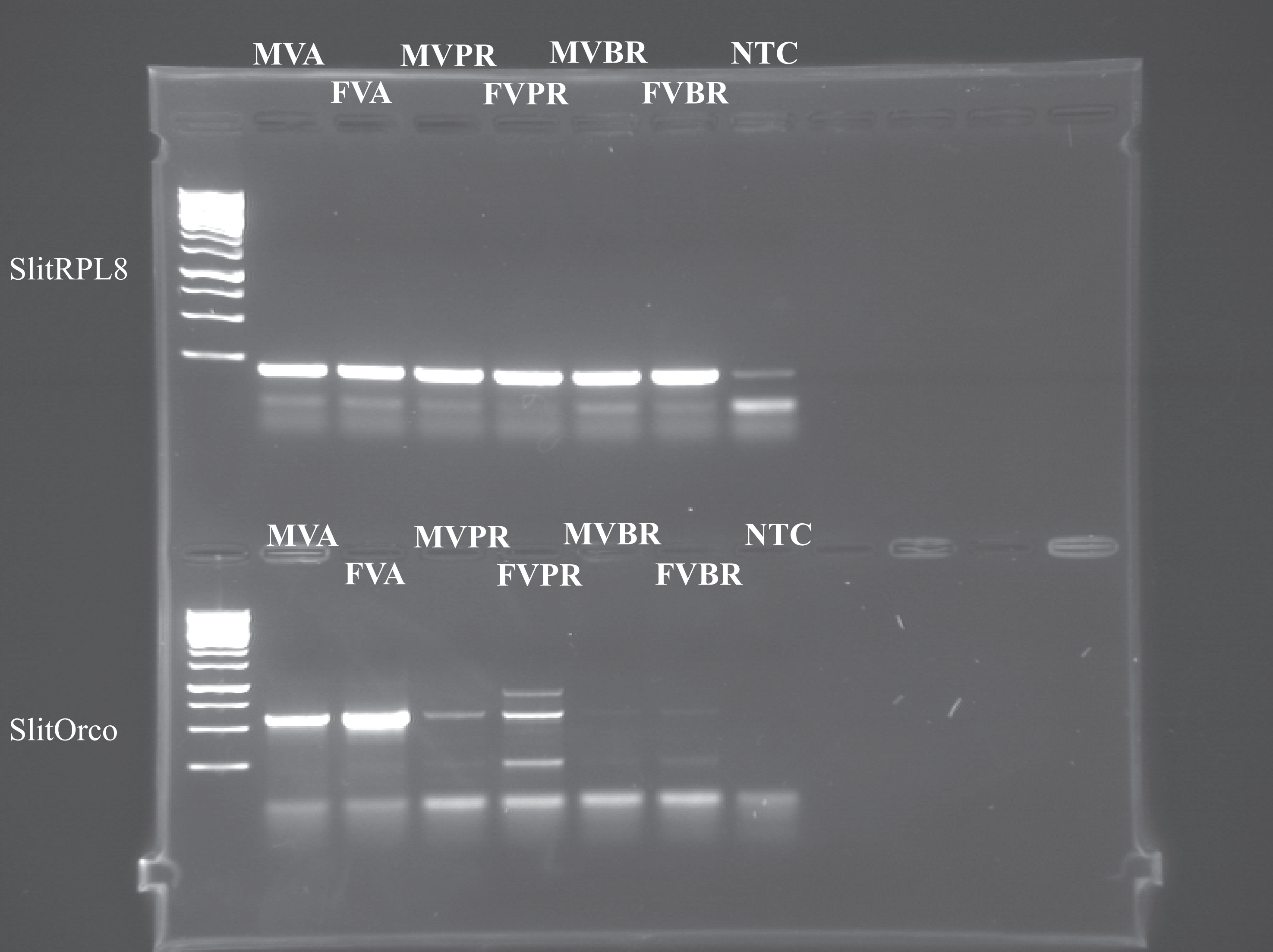
MVA
FVA
MVPR
FVPR
MVBR
FVBR
NTC
SlitRPL8
MVA
FVA
MVPR
FVPR
MVBR
FVBR
NTC
SlitOrco

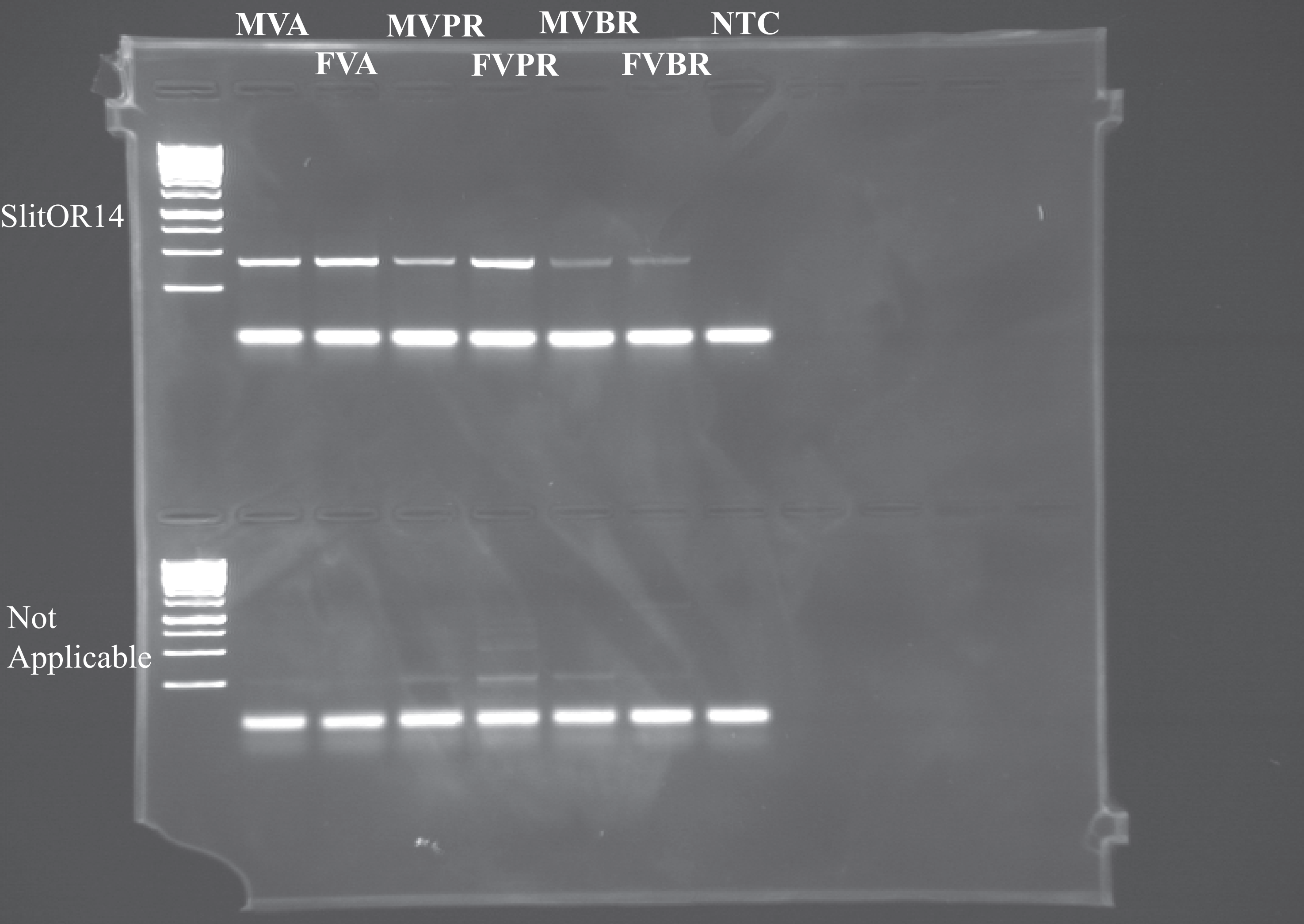
MVA
FVA
MVPR
FVPR
MVBR
FVBR
NTC
SlitOR14
Not
Applicable

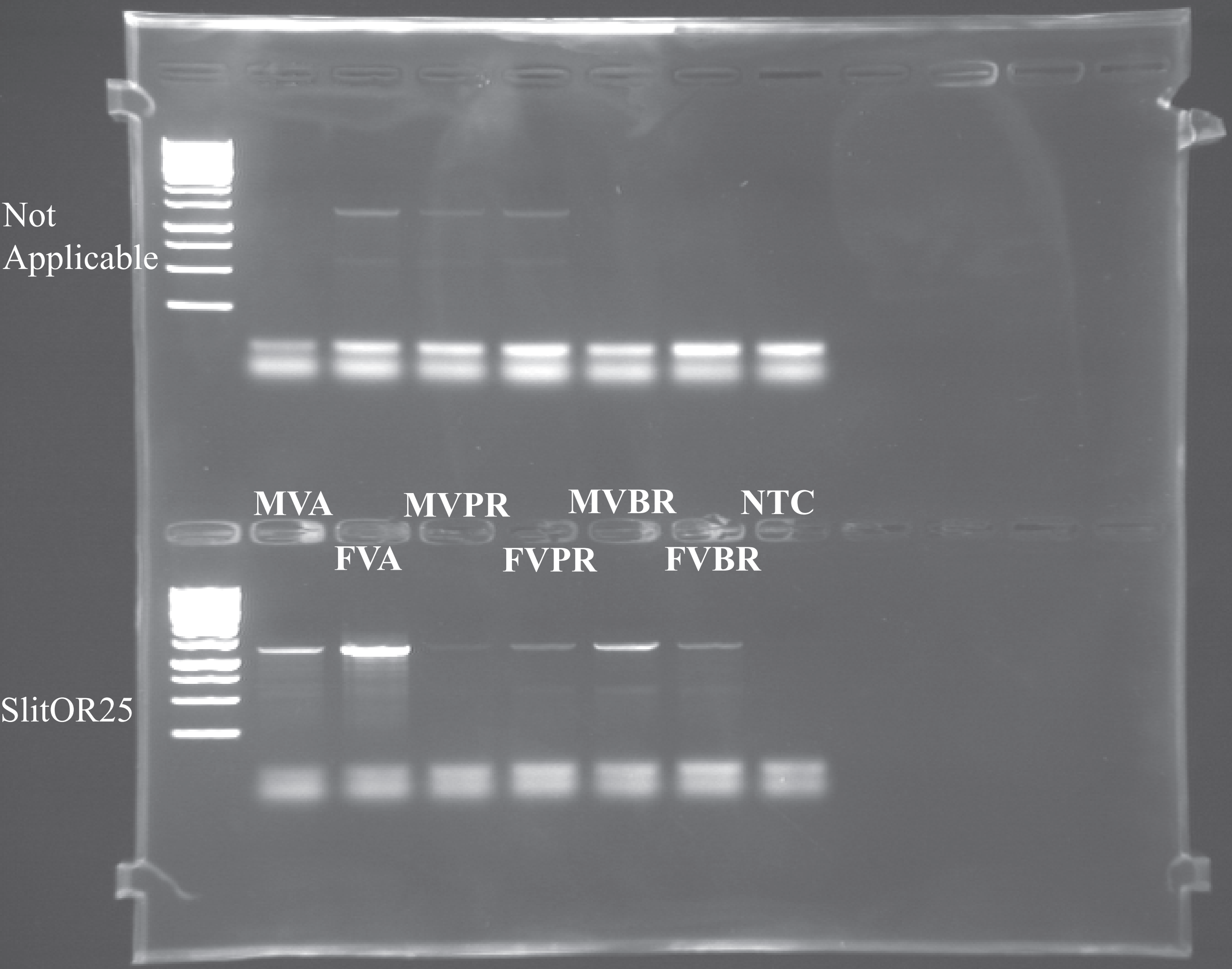
Not Applicable
MVA
FVA
MVPR
FVPR
MVBR
FVBR
NTC
SlitOR25

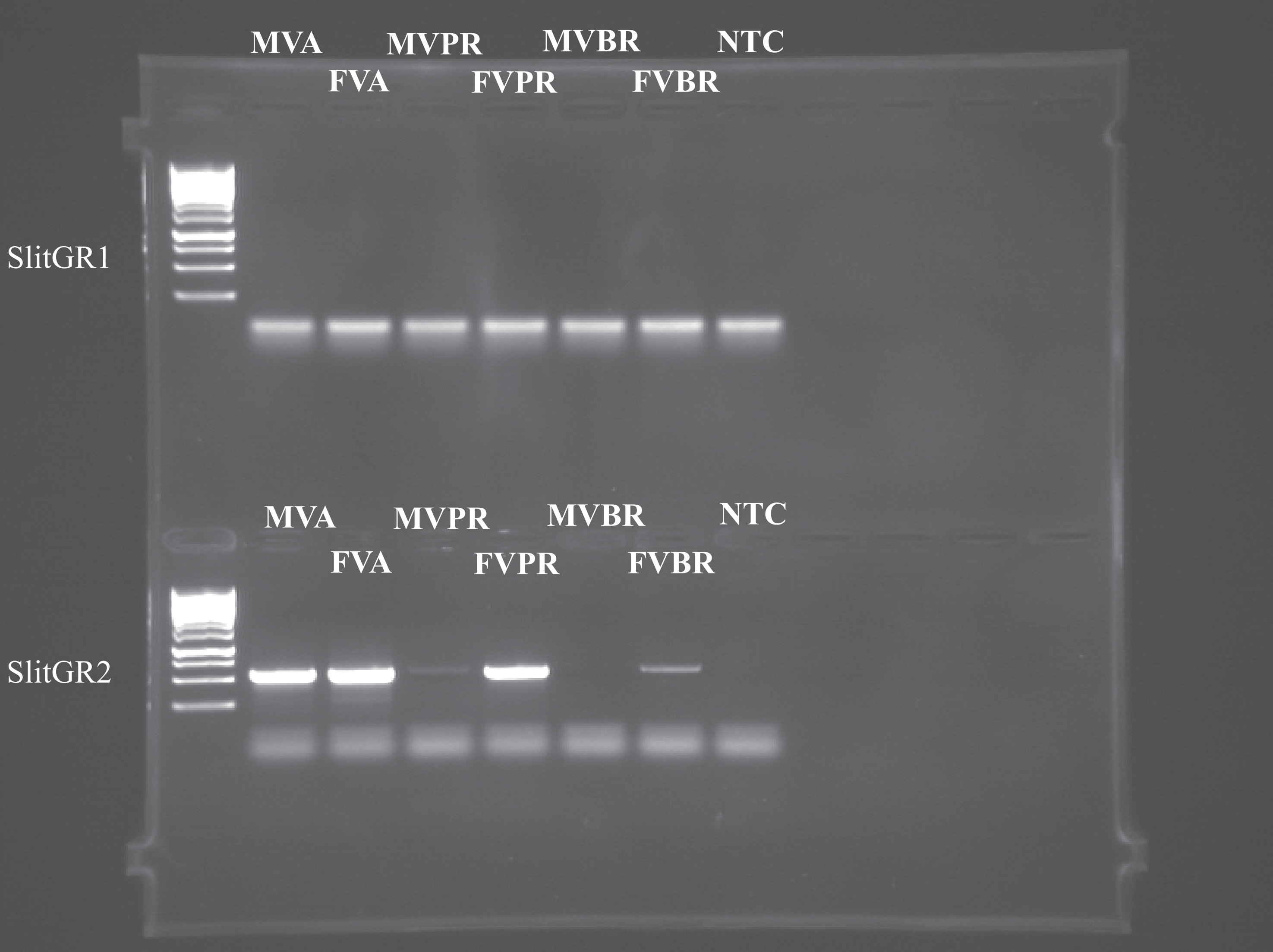
MVA
MVPR
MVBR
NTC
FVA
FVPR
FVBR
SlitGR1
MVA
MVPR
MVBR
NTC
FVA
FVPR
FVBR
SlitGR2

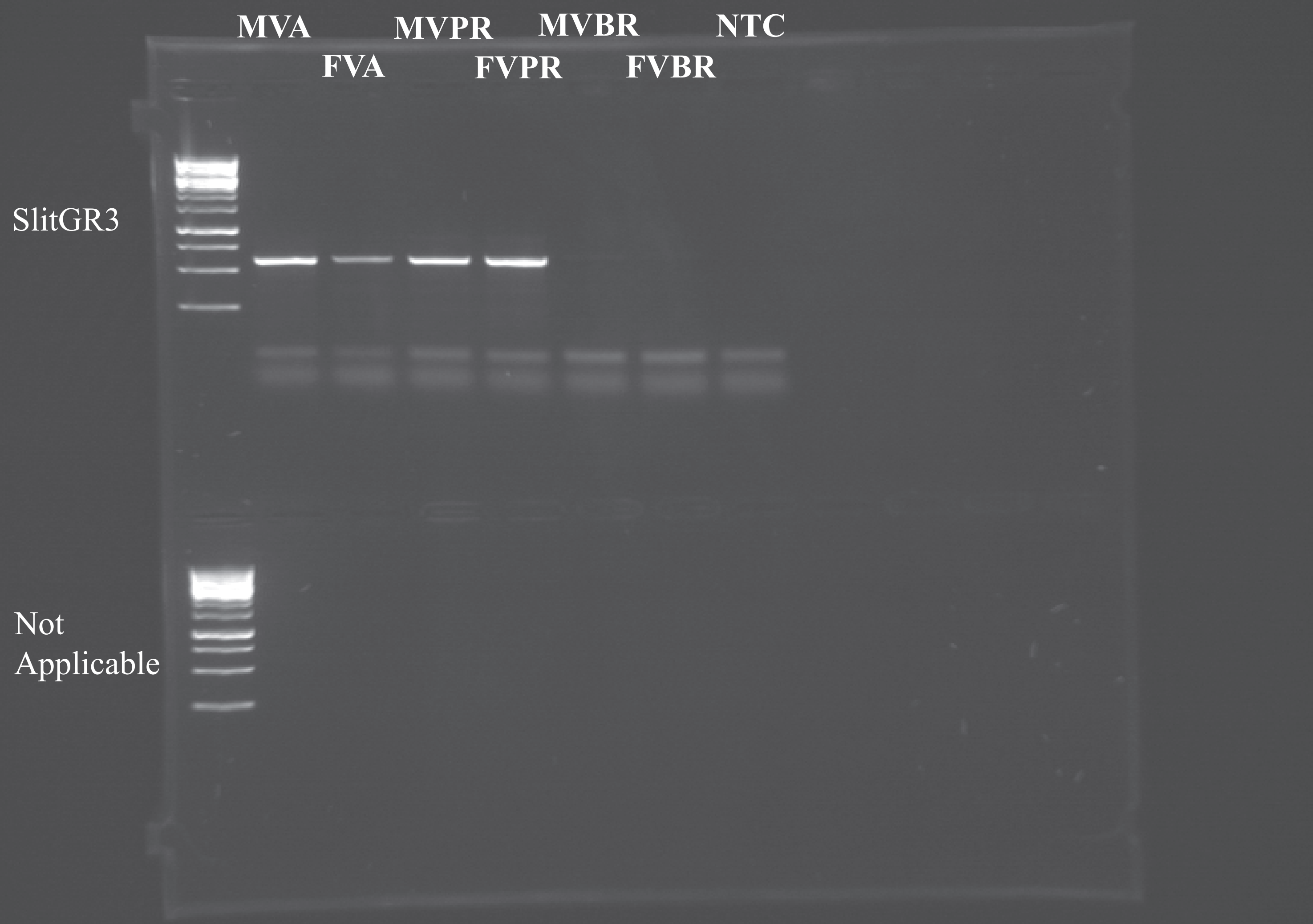
MVA
FVA
MVPR
FVPR
MVBR
FVBR
NTC
SlitGR3
Not Applicable

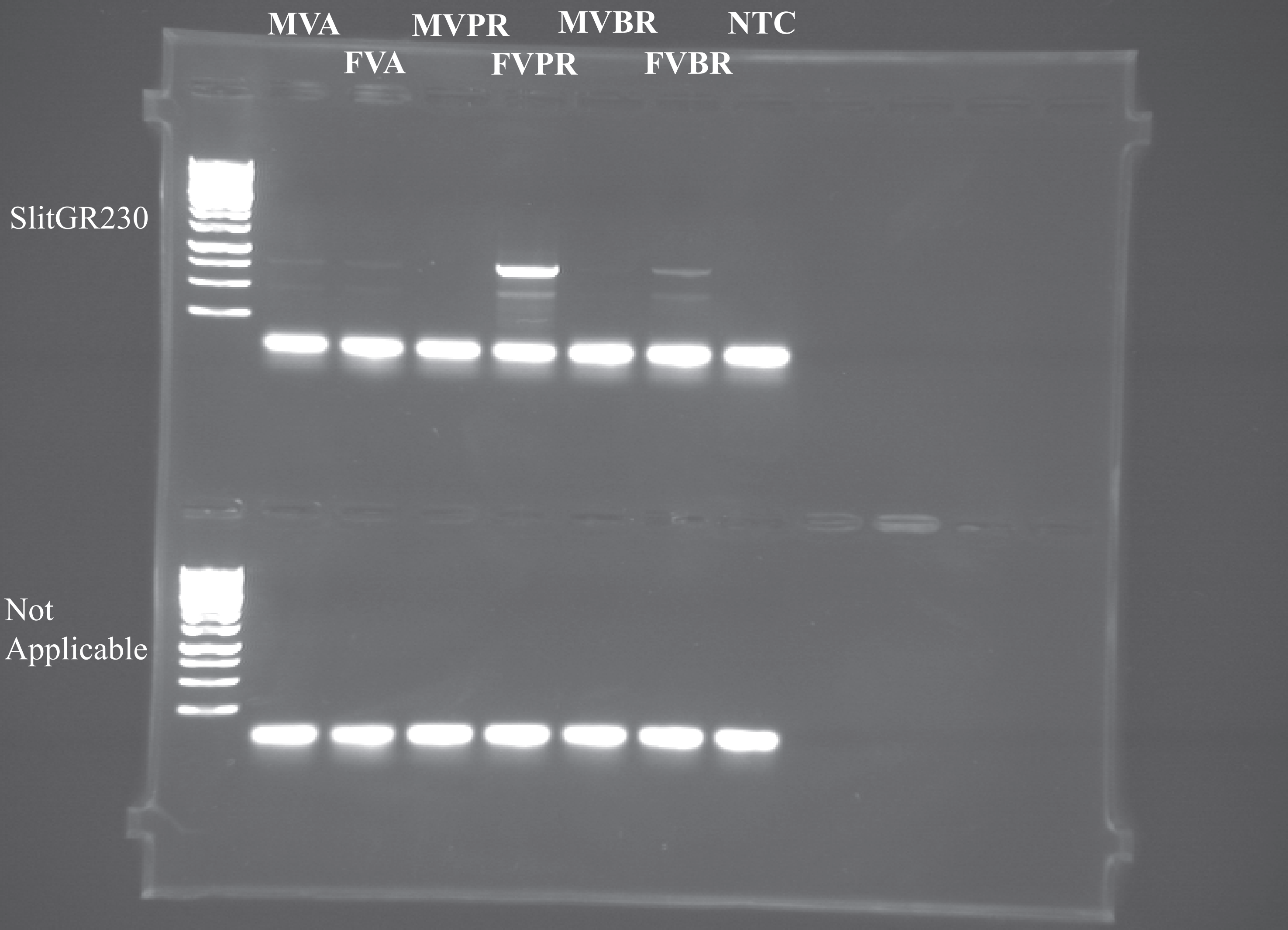
MVA
FVA
MVPR
FVPR
MVBR
FVBR
NTC
SlitGR230
Not Applicable

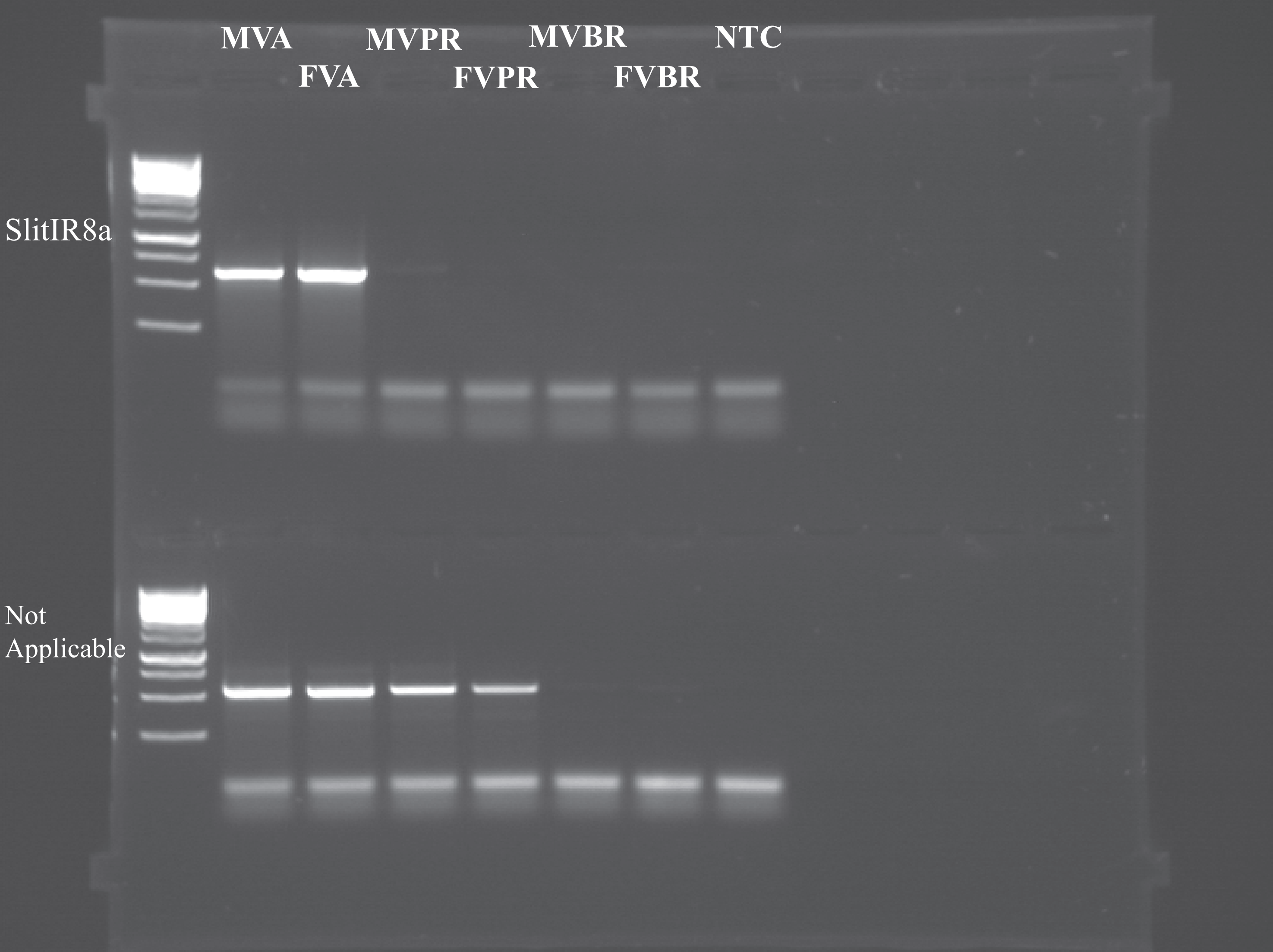
MVA
FVA
MVPR
FVPR
MVBR
FVBR
NTC
SlitIR8a
Not Applicable

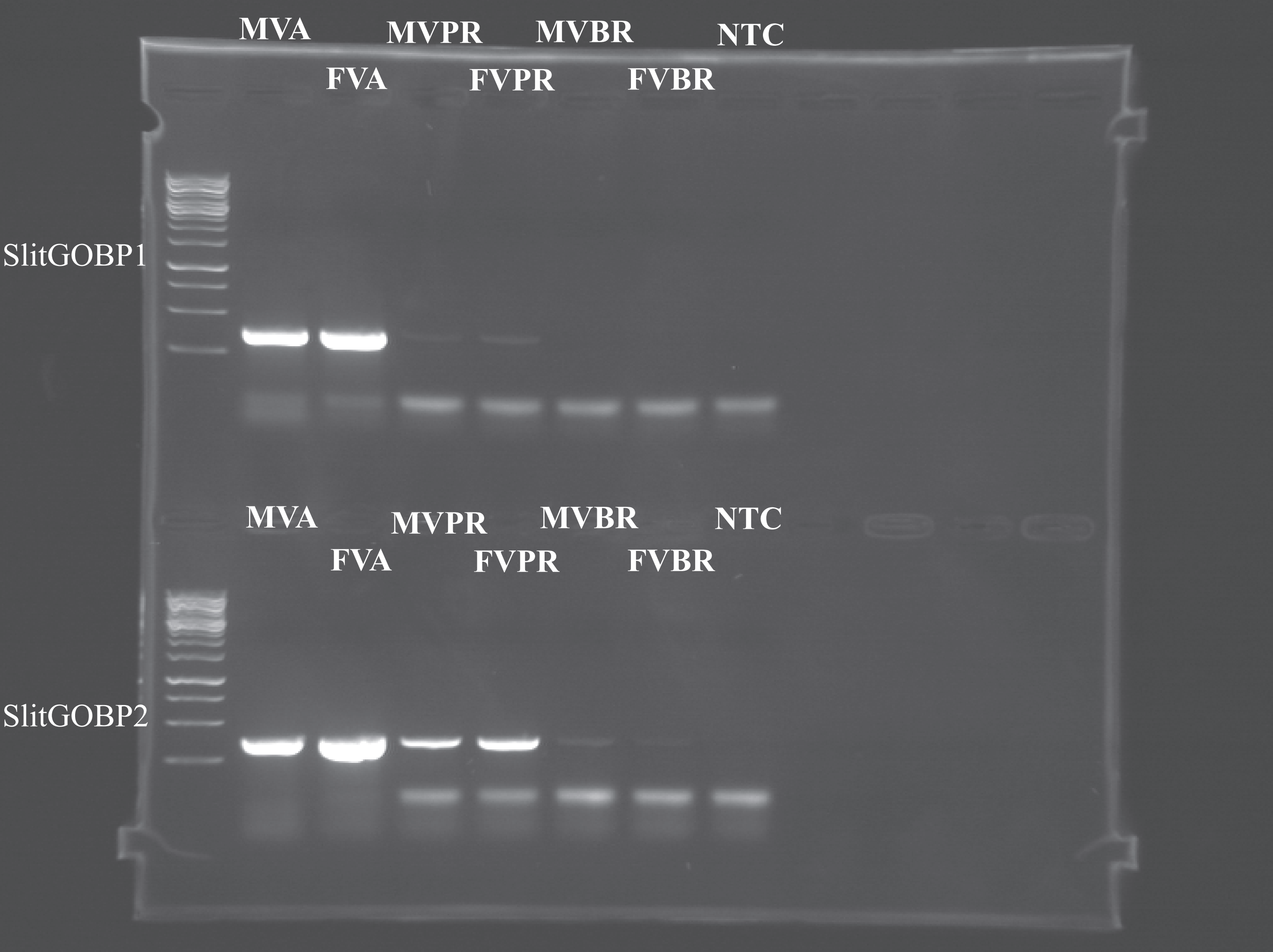
MVA
FVA
MVPR
FVPR
MVBR
FVBR
NTC
SlitGOBP1
MVA
FVA
MVPR
FVPR
MVBR
FVBR
NTC
SlitGOBP2

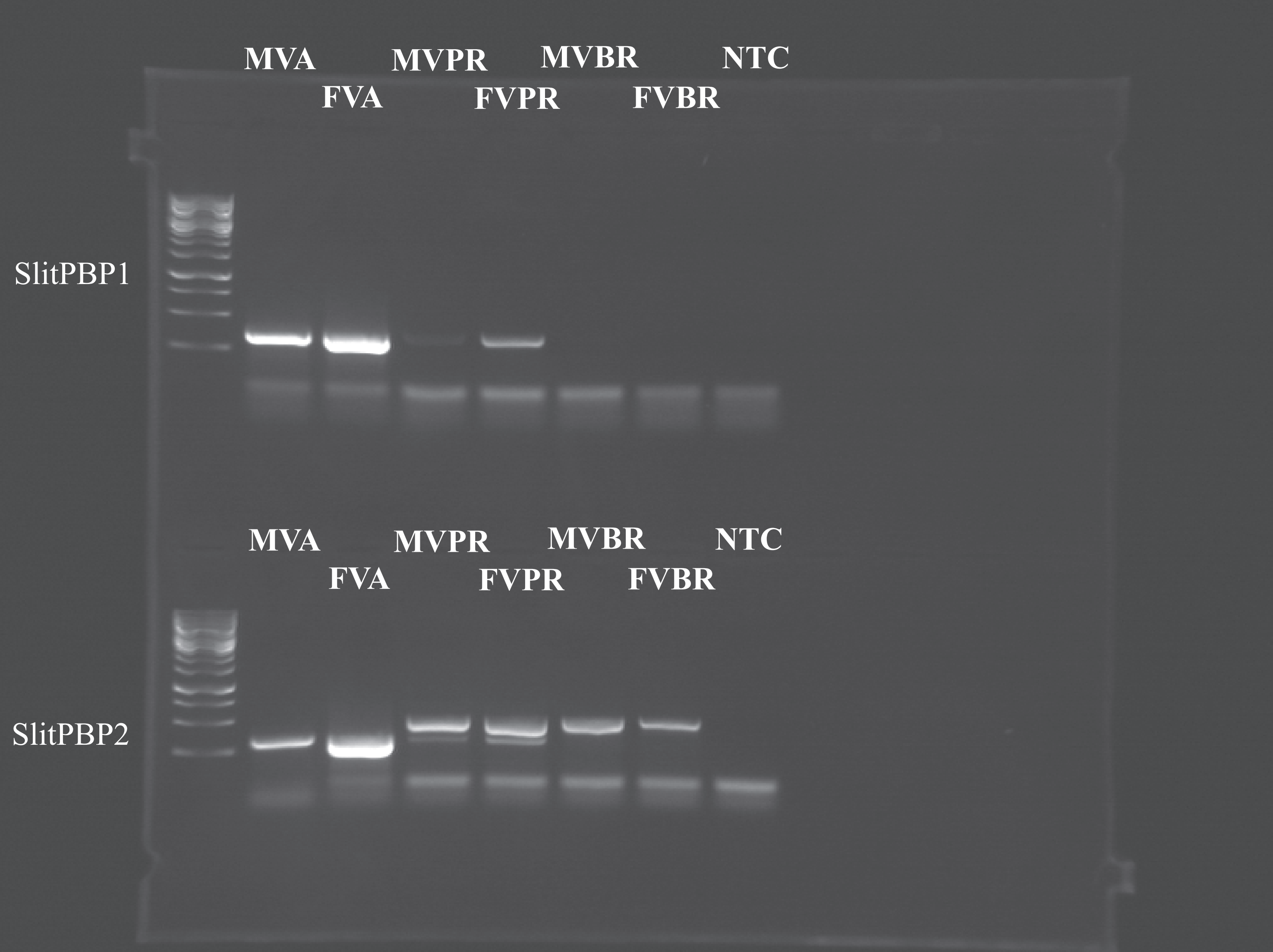
MVA
FVA
MVPR
FVPR
MVBR
FVBR
NTC
SlitPBP1
MVA
FVA
MVPR
FVPR
MVBR
FVBR
NTC
SlitPBP2

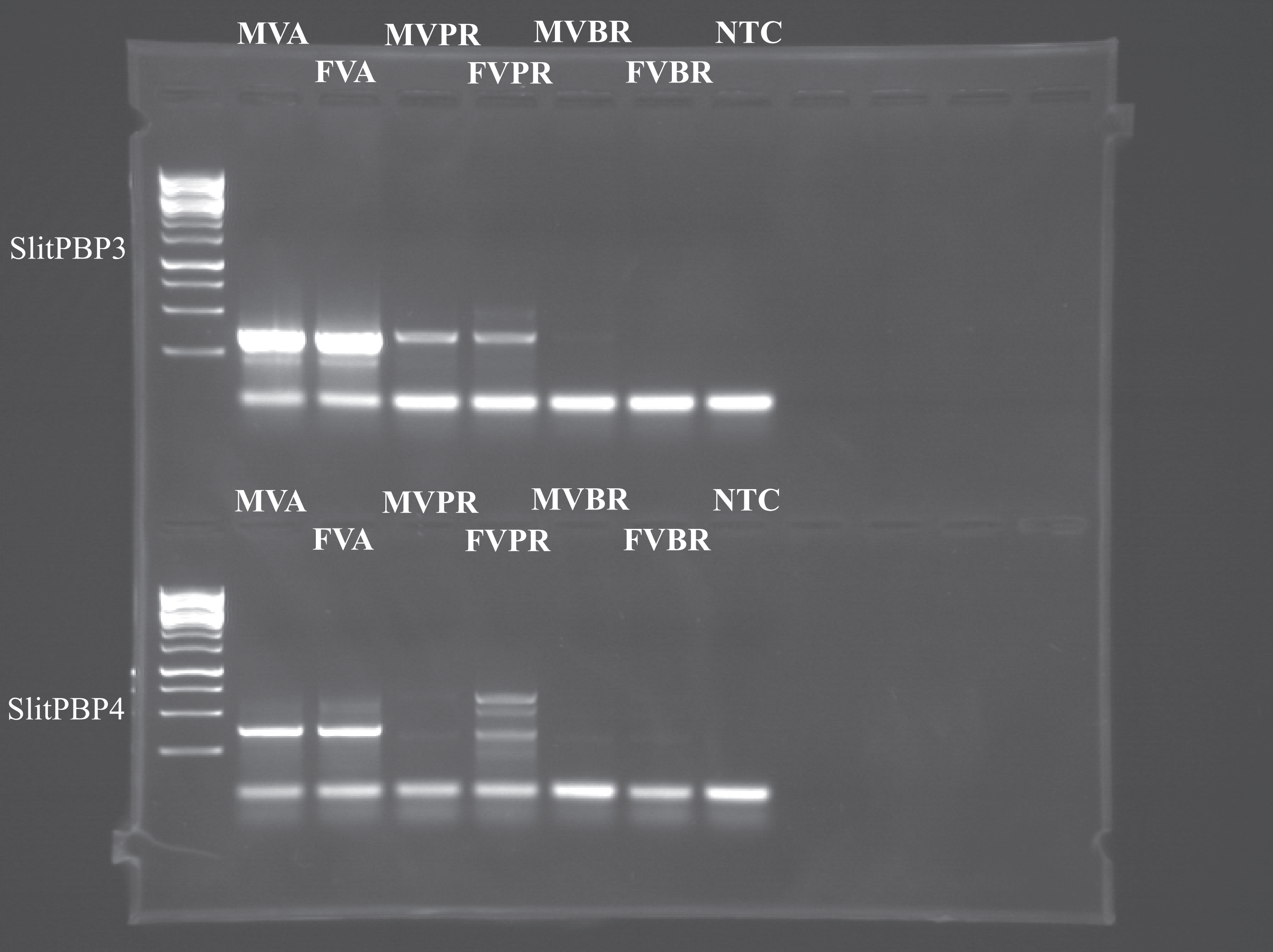
MVA
FVA
MVPR
FVPR
MVBR
FVBR
NTC
SlitPBP3
MVA
FVA
MVPR
FVPR
MVBR
FVBR
NTC
SlitPBP4

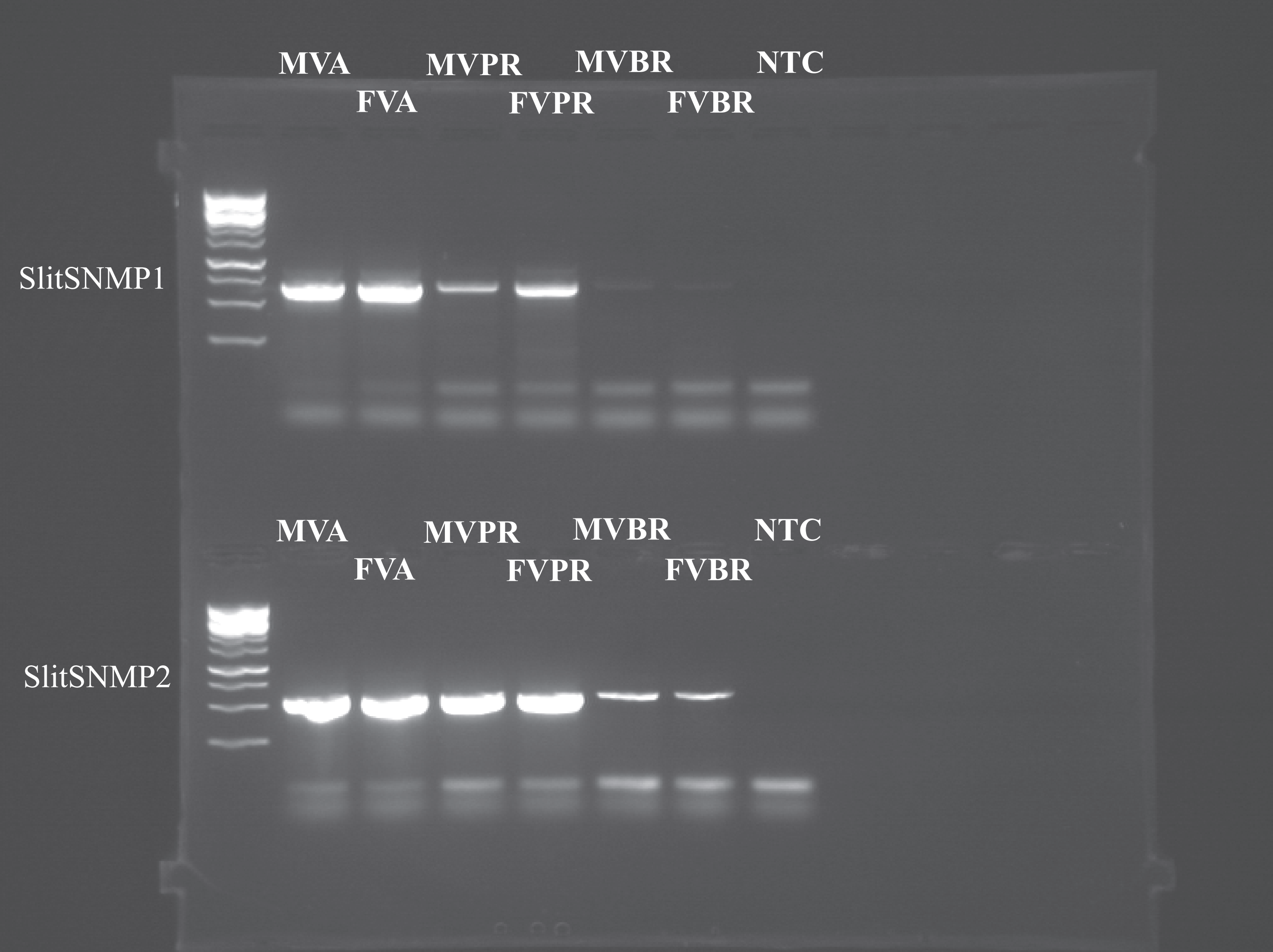
MVA
FVA
MVPR
FVPR
MVBR
FVBR
NTC
SlitSNMP1
MVA
FVA
MVPR
FVPR
MVBR
FVBR
NTC
SlitSNMP2
