# Supplementary material for: Transcriptome Analysis of Gene Families Involved in Chemosensory Function in Spodoptera littoralis (Lepidoptera: Noctuidae)
Source: BMC Genomics. 2019 May 28;20:428. doi: 10.1186/s12864-019-5815-x (PMC6540431; doi:10.1186/s12864-019-5815-x)
Supplement: Supplementary file 24 — uncropped PCR gels, including all no-RT controls. For all gels, 1kb Gene Ruler ladder (Thermo Fisher Scientific) was used. MVA – male virgin antennae, FVA – female virgin antennae, MVPR – male virgin proboscis, FVPR, female virgin proboscis, MVBR – male virgin brain, FVBR – female virgin brain, N/A – not applicable. On some gels, part of the gel space was used for experiments unrelated to this manuscript; those sections are indicated as “not applicable.” (PDF 3777 kb) [file 12864_2019_5815_MOESM24_ESM.pdf]

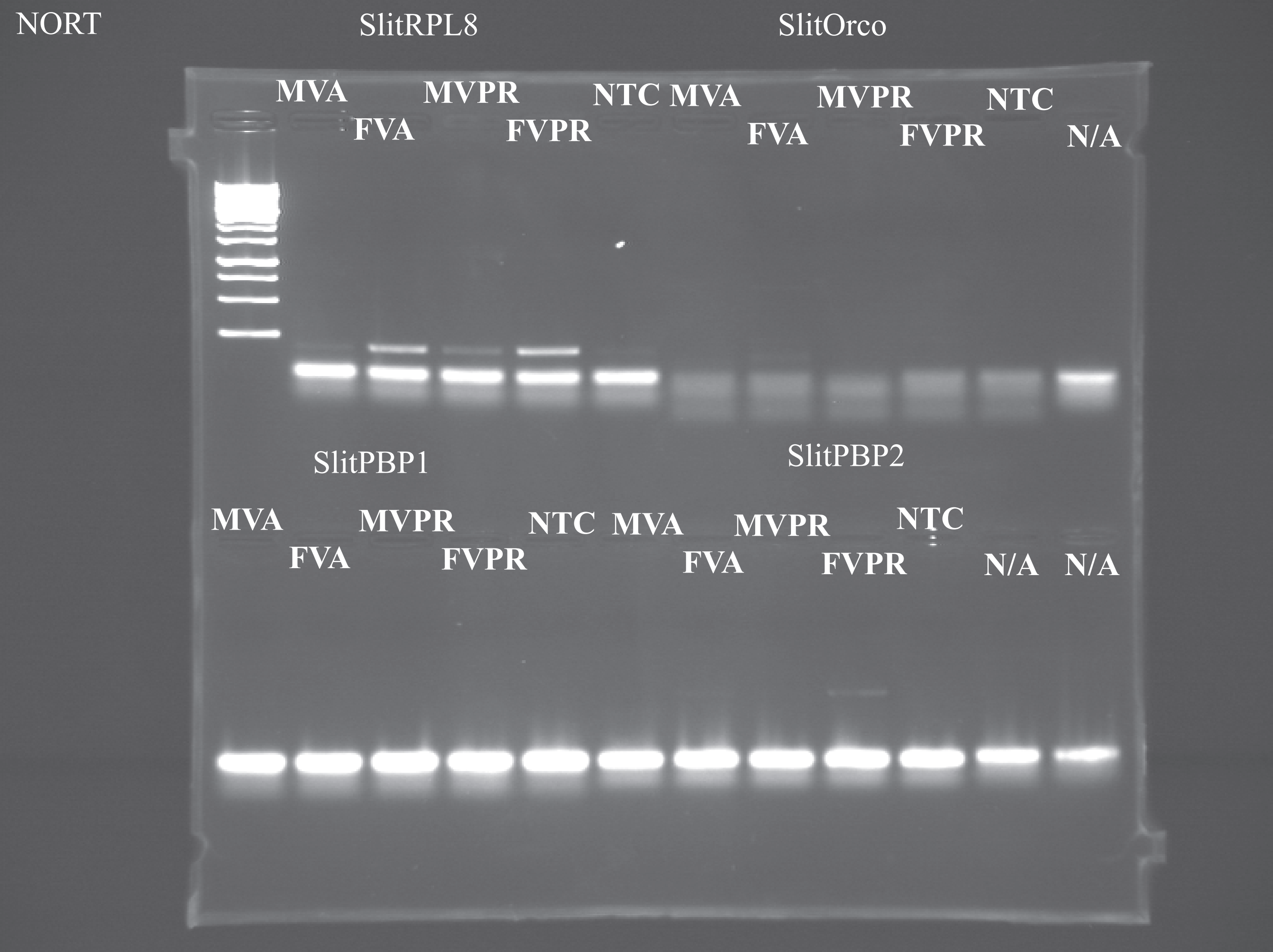
NORT
SlitRPL8
SlitOrco
MVA
FVA
MVPR
FVPR
NTC
MVA
FVA
MVPR
FVPR
NTC
N/A
SlitPBP1
SlitPBP2
MVA
FVA
MVPR
FVPR
NTC
MVA
FVA
MVPR
FVPR
NTC
N/A
N/A

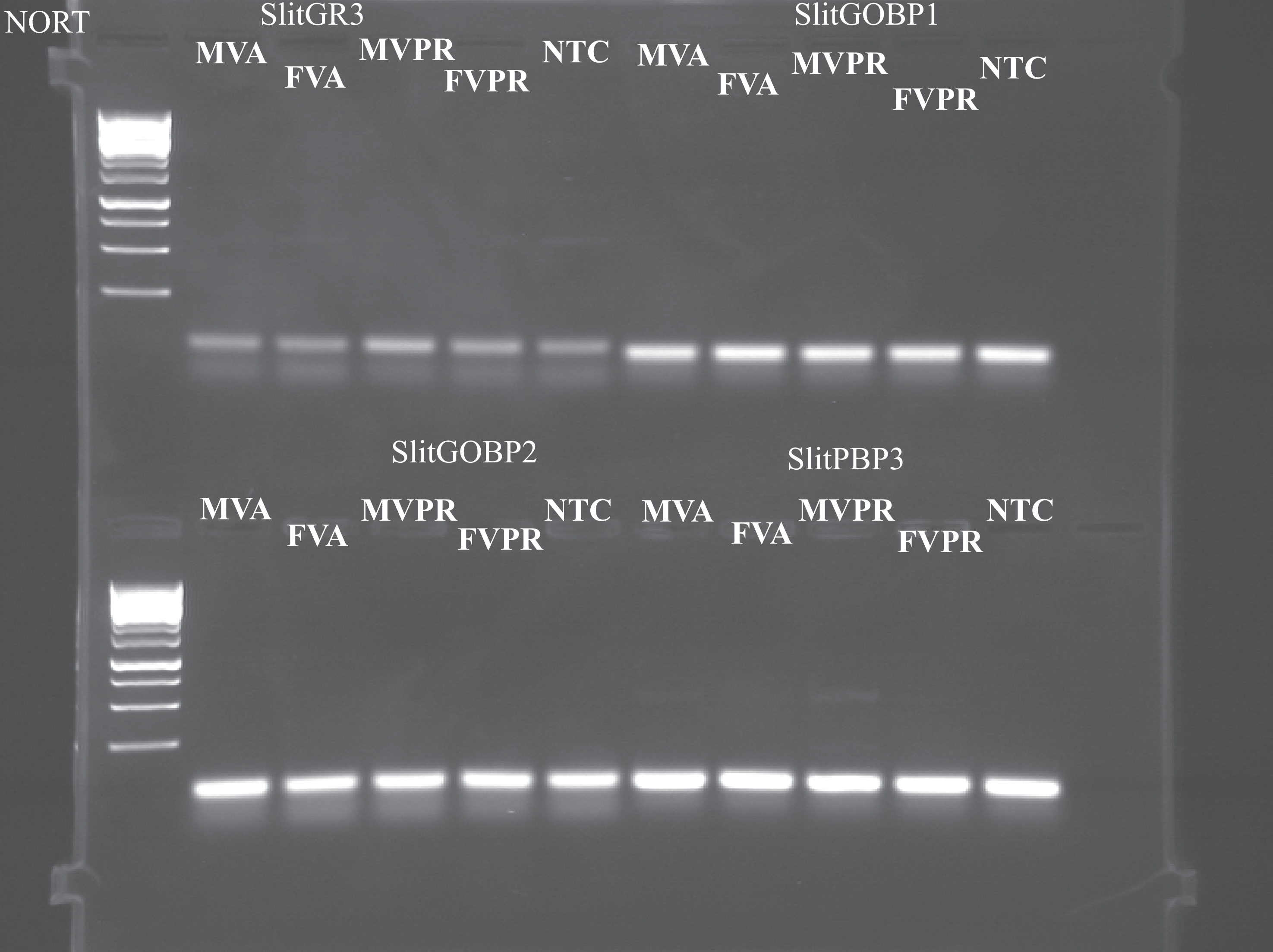
NORT
SlitGR3
MVA
FVA
MVPR
FVPR
NTC
SlitGOBP1
MVA
FVA
MVPR
FVPR
NTC
SlitGOBP2
MVA
FVA
MVPR
FVPR
NTC
SlitPBP3
MVA
FVA
MVPR
FVPR
NTC

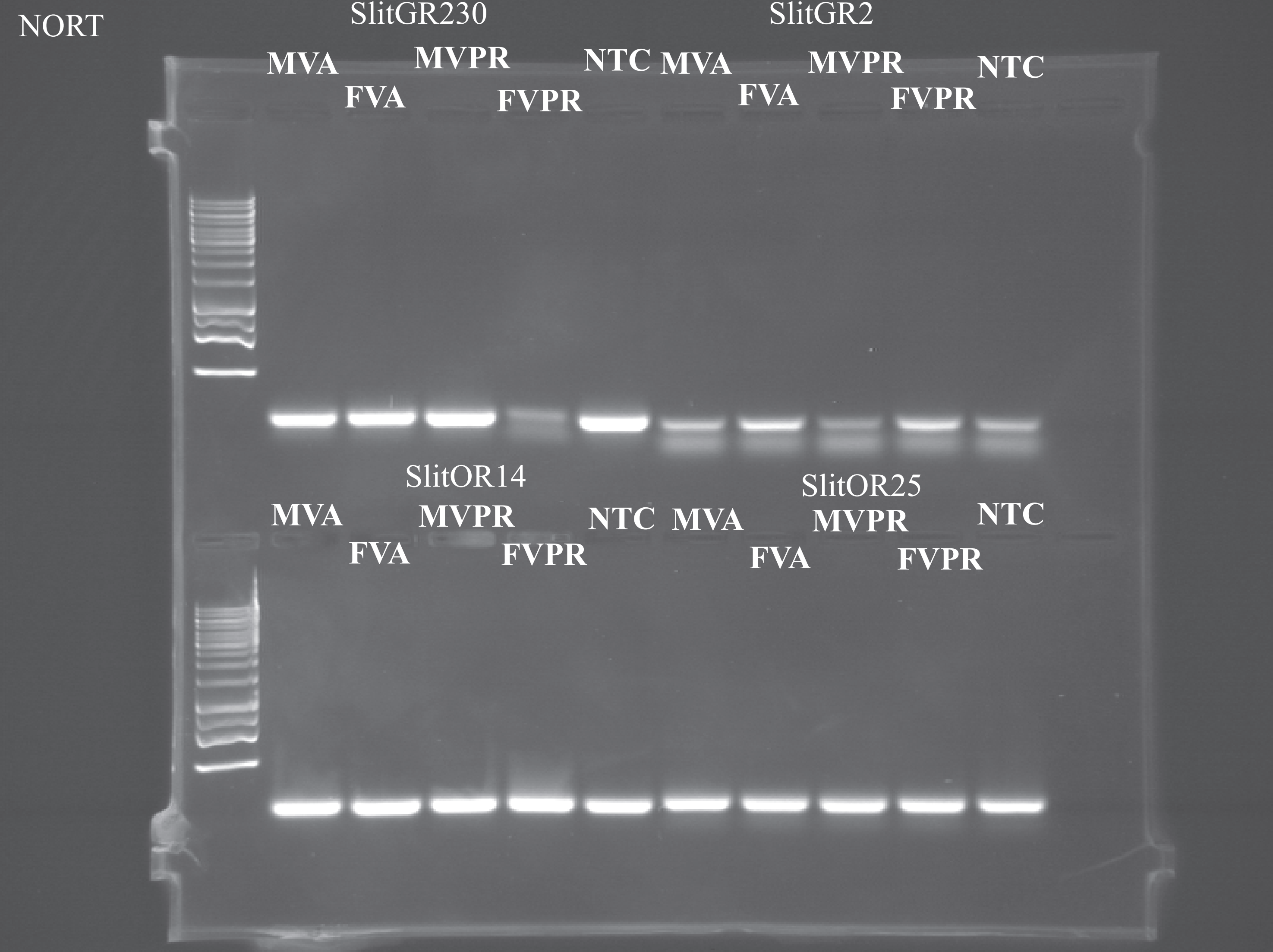
NORT
SlitGR230
SlitGR2
MVA
FVA
MVPR
FVPR
NTC
MVA
FVA
MVPR
FVPR
NTC
SlitOR14
SlitOR25
MVA
FVA
MVPR
FVPR
NTC
MVA
FVA
MVPR
FVPR
NTC

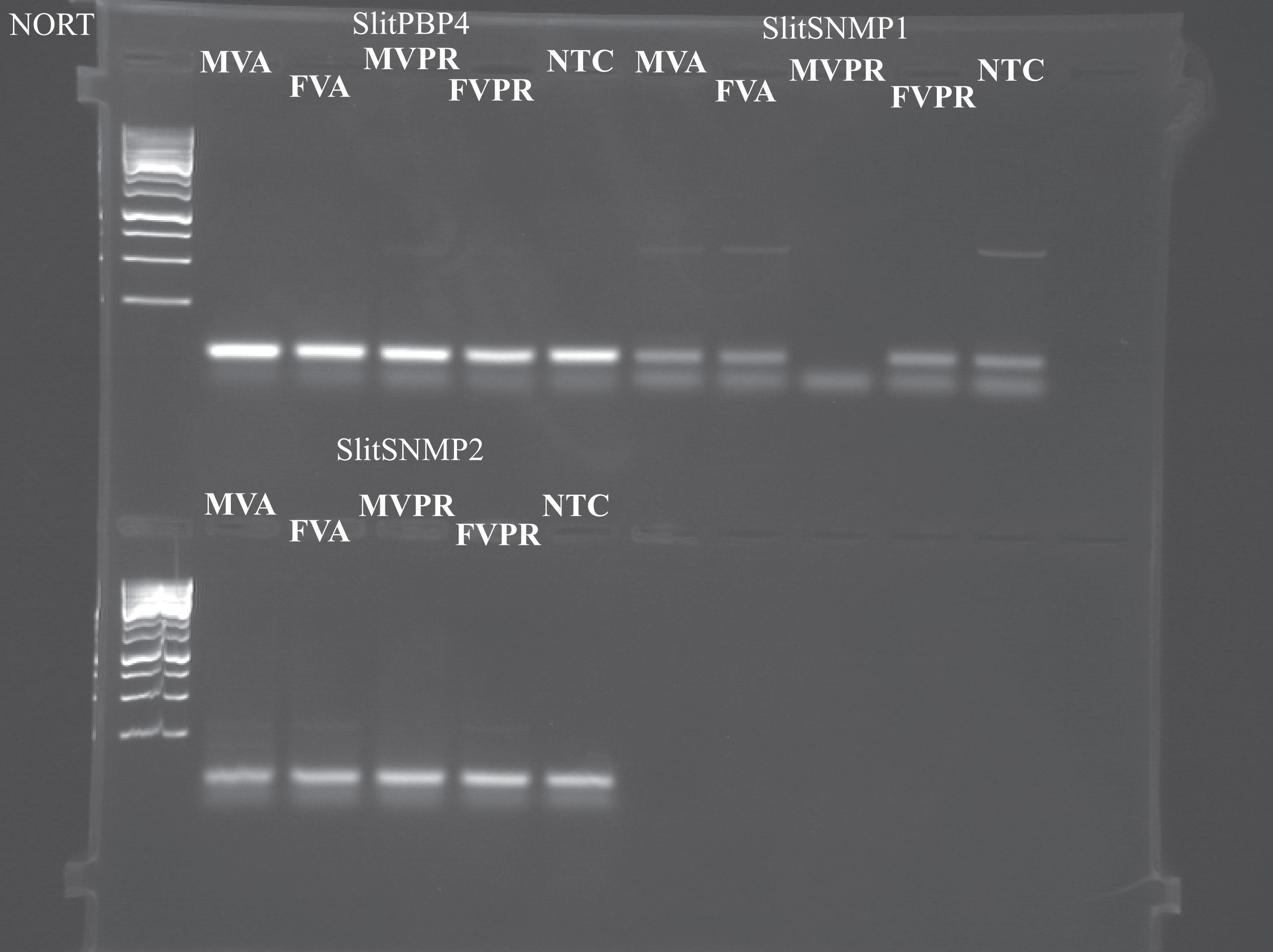
NORT
SlitPBP4
MVA
FVA
MVPR
FVPR
NTC
SlitSNMP1
MVA
FVA
MVPR
FVPR
NTC
SlitSNMP2
MVA
FVA
MVPR
FVPR
NTC

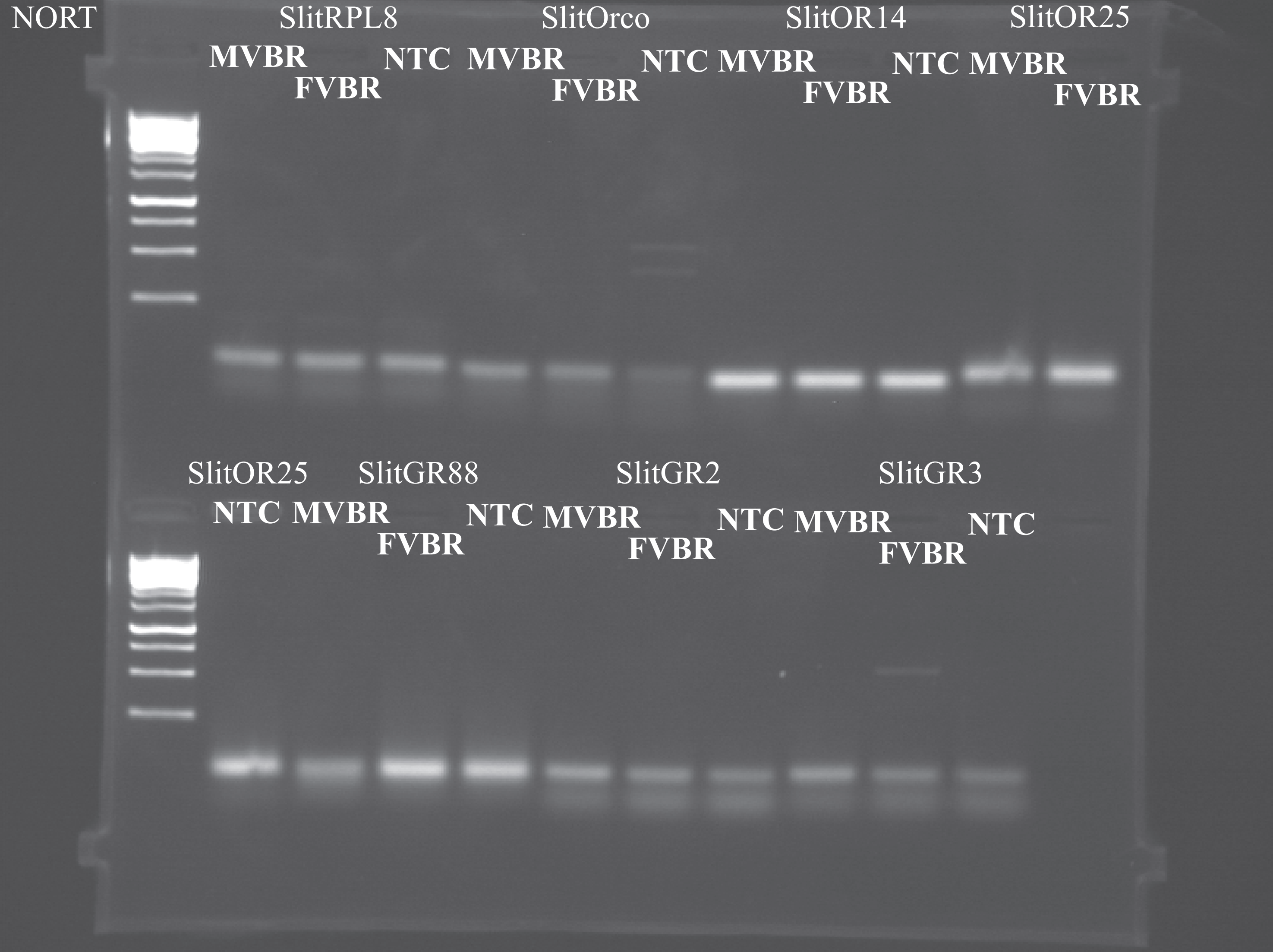
NORT
SlitRPL8
SlitOrco
SlitOR14
SlitOR25
MVBR
FVBR
NTC
MVBR
FVBR
NTC
MVBR
FVBR
NTC
MVBR
FVBR
SlitOR25
SlitGR88
SlitGR2
SlitGR3
NTC
MVBR
FVBR
NTC
MVBR
FVBR
NTC
MVBR
FVBR
NTC

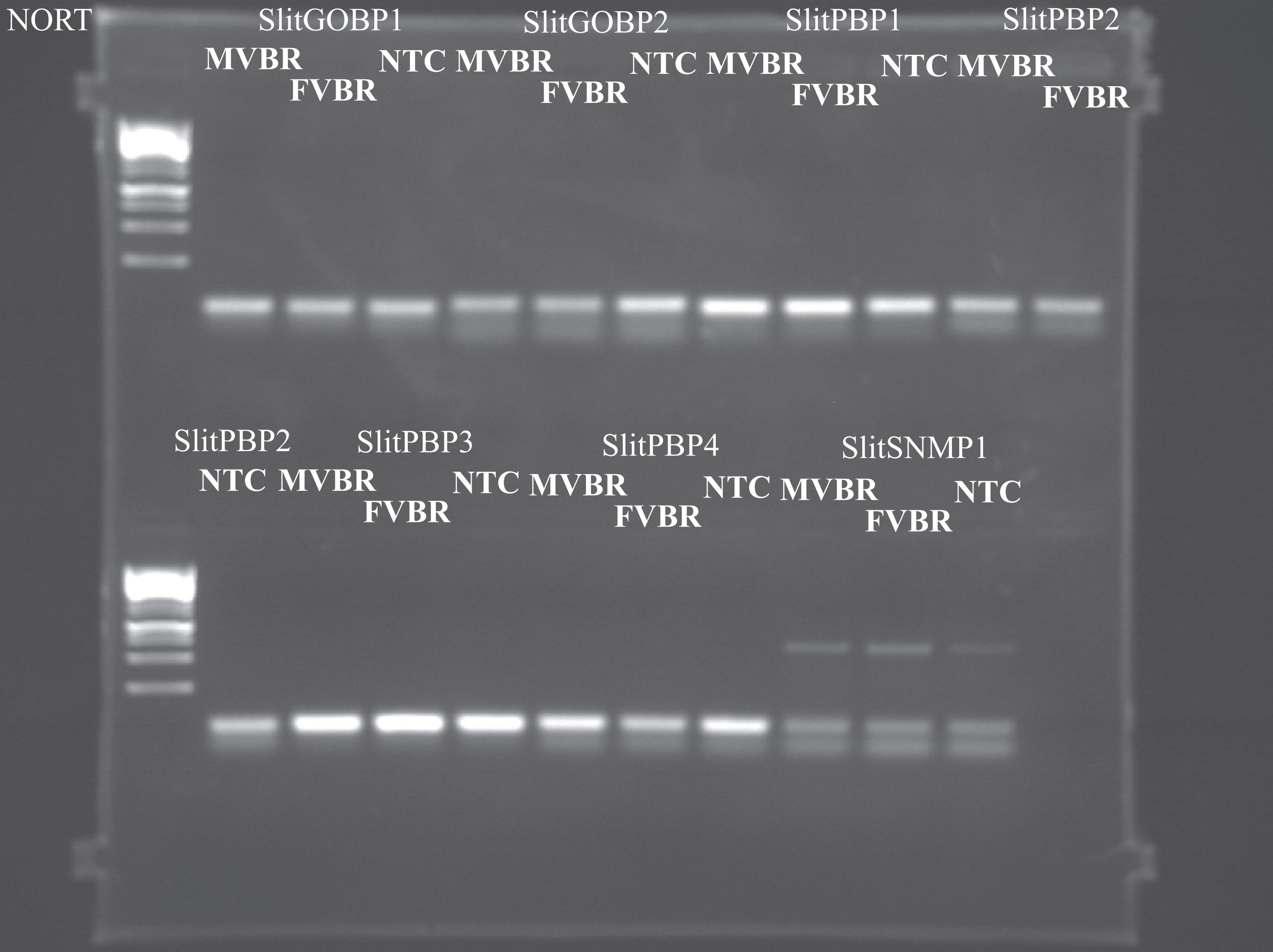
NORT
SlitGOBP1
MVBR
FVBR
NTC
SlitGOBP2
MVBR
FVBR
NTC
SlitPBP1
MVBR
FVBR
NTC
SlitPBP2
MVBR
FVBR
SlitPBP2
NTC
SlitPBP3
MVBR
FVBR
NTC
SlitPBP4
MVBR
FVBR
NTC
SlitSNMP1
MVBR
FVBR
NTC

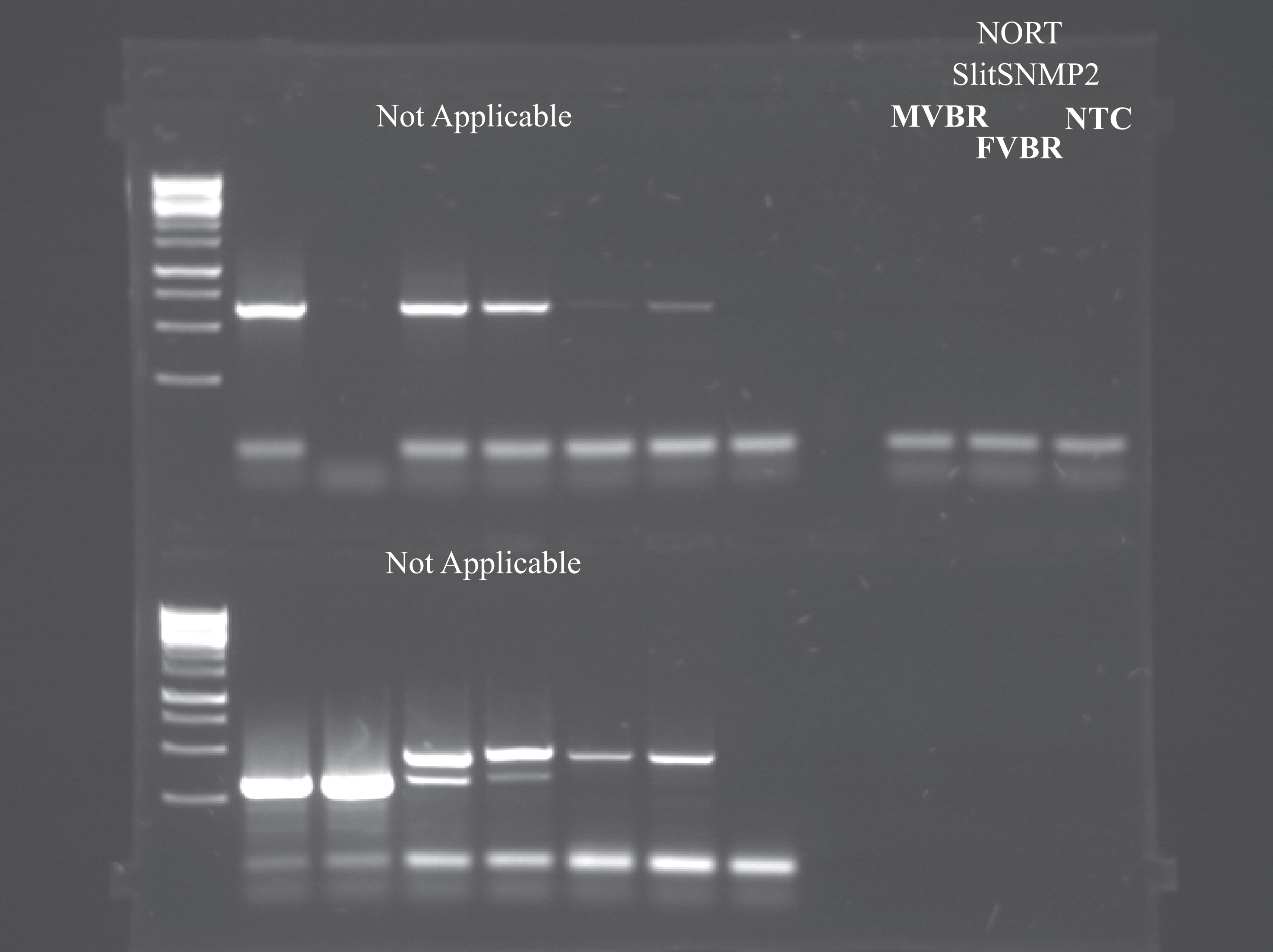
NORT
SlitSNMP2
Not Applicable
MVBR
FVBR
NTC
Not Applicable

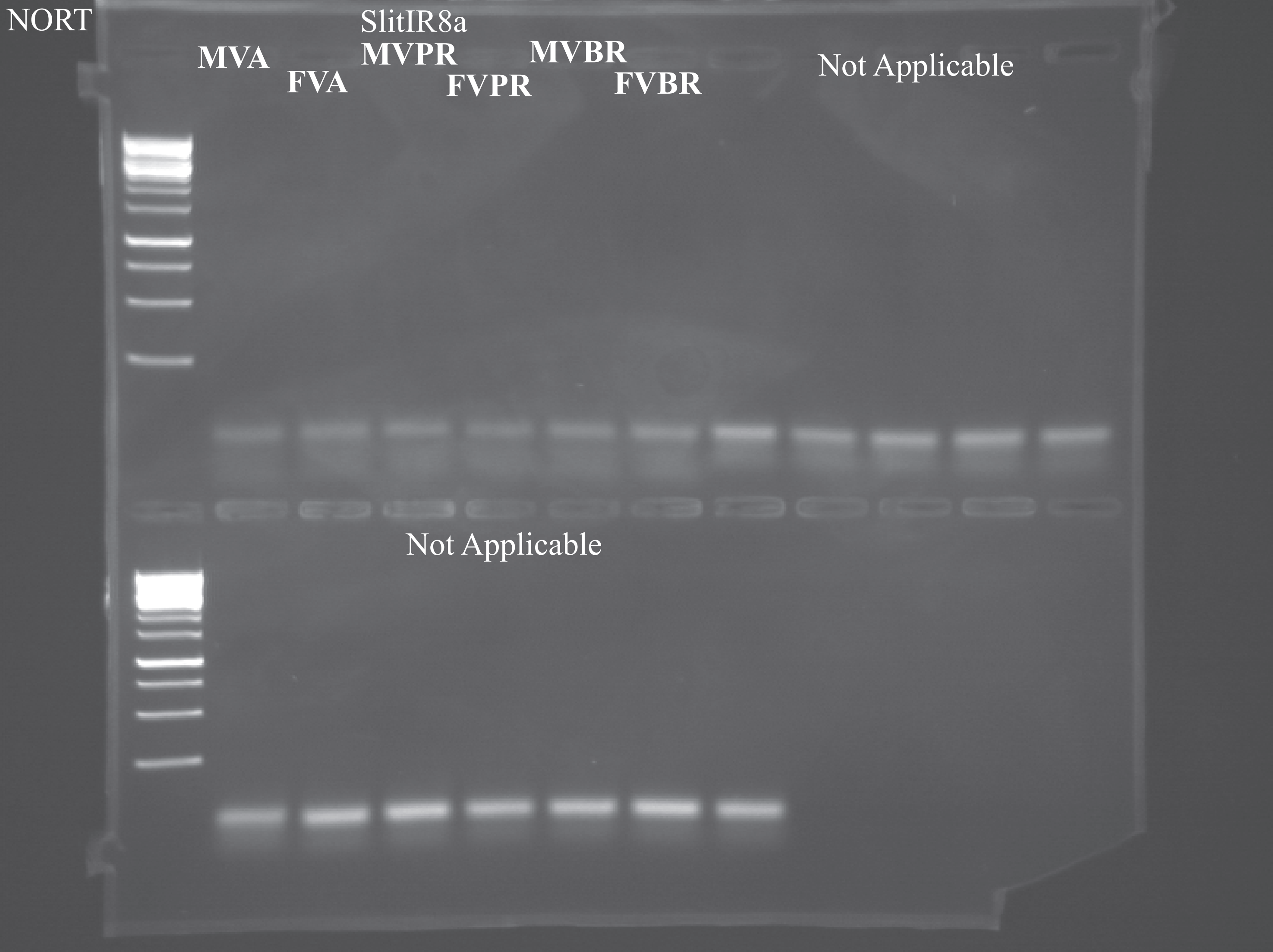
NORT
SlitIR8a
MVA
FVA
MVPR
FVPR
MVBR
FVBR
Not Applicable
Not Applicable
